# Supplementary material for: Genomic bases of short-term evolution in wild barn owls revealed by long-term monitoring and population-scale sequencing
Source: Nat Commun. 2026 Oct 12;17:11545. doi: 10.1038/s41467-026-78632-4 (PMC13748587; doi:10.1038/s41467-026-78632-4)
Supplement: Supplementary file 1 [file 41467_2026_78632_MOESM1_ESM.pdf]

Genomic bases of short-term evolution in wild barn owls revealed by long-term monitoring and population-scale sequencing

Supplementary Materials

Phenotypic changes in the two melanic traits 2

Color and Spottiness variation in the Swiss barn owl 2

Evolution of two melanin-based traits.. 5

Population-wide whole genome sequencing of the Swiss population 14

Genetic diversity in reference panel.. 14

Phasing of the reference panel 15

Low coverage sequencing of 2778 owls... 17

Heritability of the coloration and the Spottiness 18

Genome Wide Association Study 20

GWAS – Plumage coloration 20

GWAS – ventral spottiness 22

Differential gene expression links genotype to phenotype 23

Genotype, gene expression and phenotype.. 23

Sexual dimorphism and sex specific genetic architecture... 28

Genetic architecture of coloration 28

Genetic architecture of spottiness 30

Characterizing evolutionary process driving allelic and phenotypic changes 32

Observed allelic frequency changes.. 32

Evolutionary forces driving the frequency changes 34

Supplementary Note 1: Acknowledgments for Sample Collection..................................... 40

Phenotypic changes in the two melanic traits

Color and Spottiness variation in the Swiss barn owl

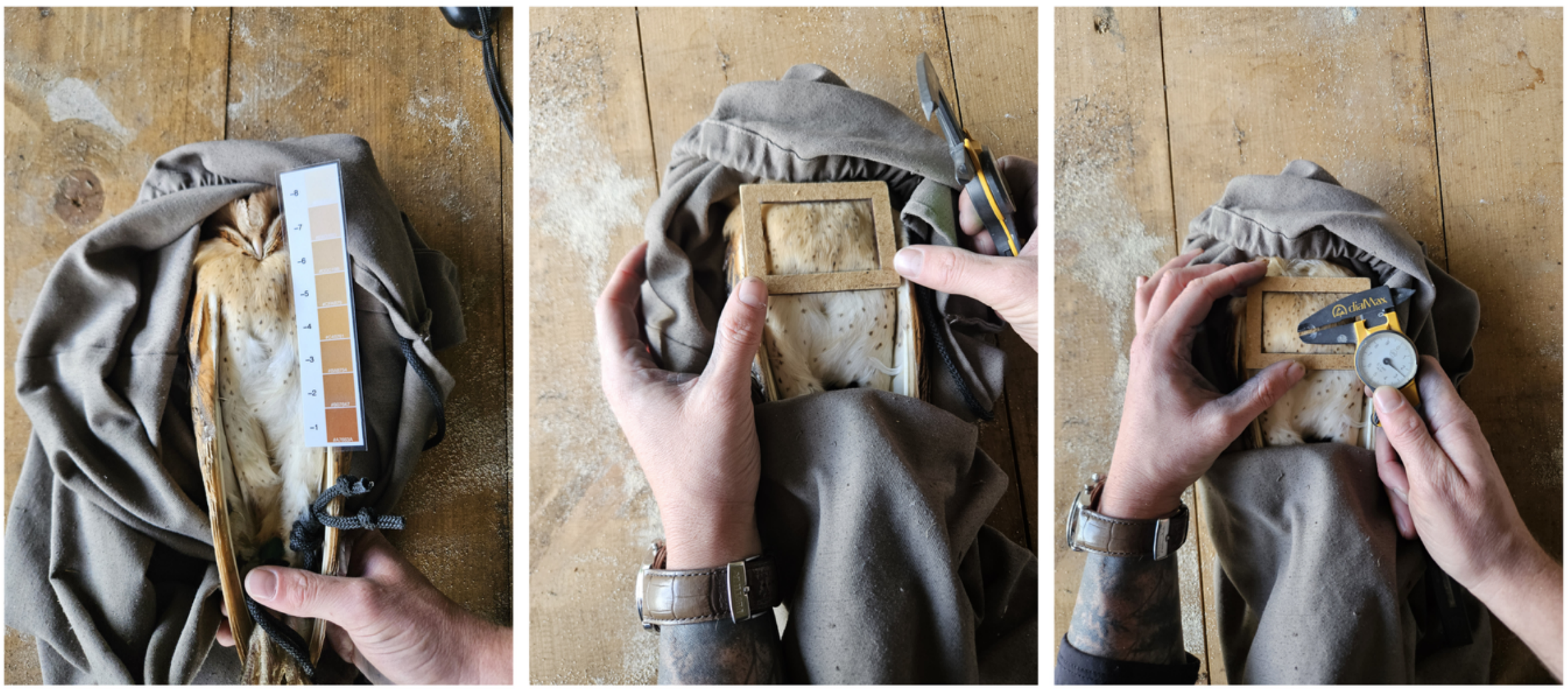

Figure S1 – Field phenotyping of an owl. Captured owls are scored using a color chip (left). Spottiness is quantified by counting the number (N) of eumelanic black spots and measuring their mean diameter (D ± 0.1 mm) within a 60 × 40 mm (2400 mm²) frame (middle and right, respectively).

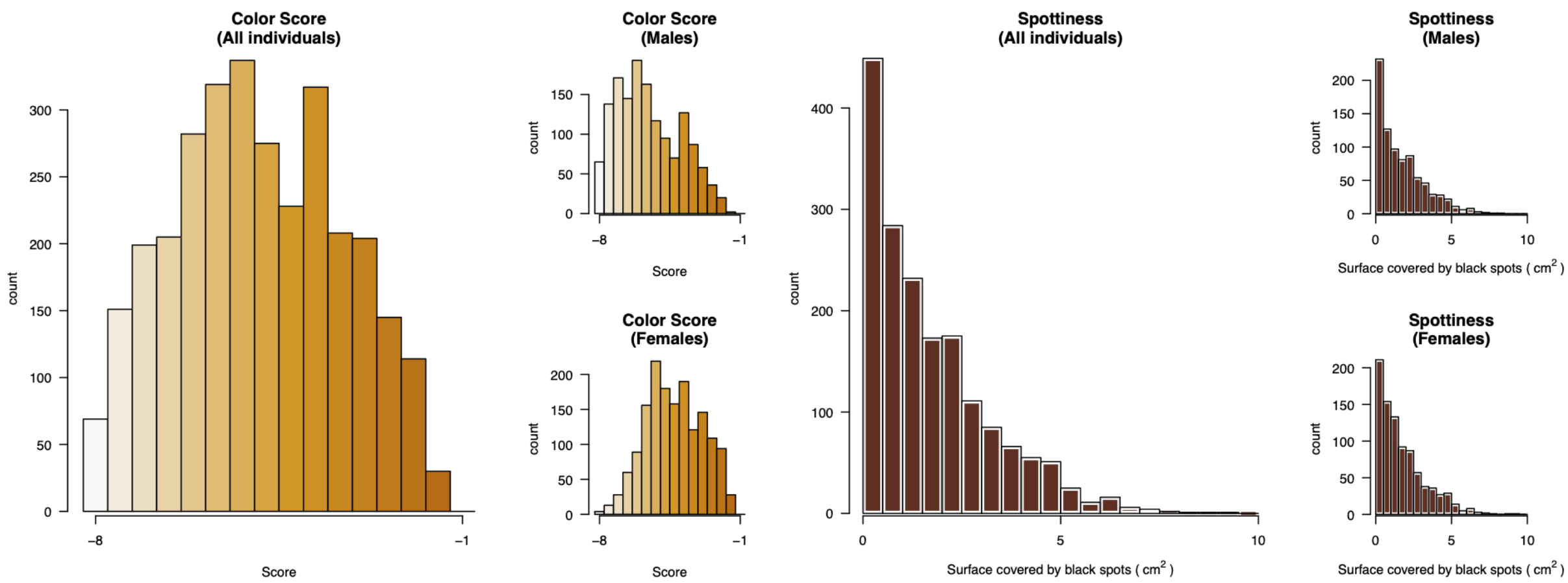

Figure S2 – Distribution of the color (left) and spottiness (right) in all individuals (n = 1916) and within each sex (top panel for males (n = 864), bottom panel for females (n = 1052)).

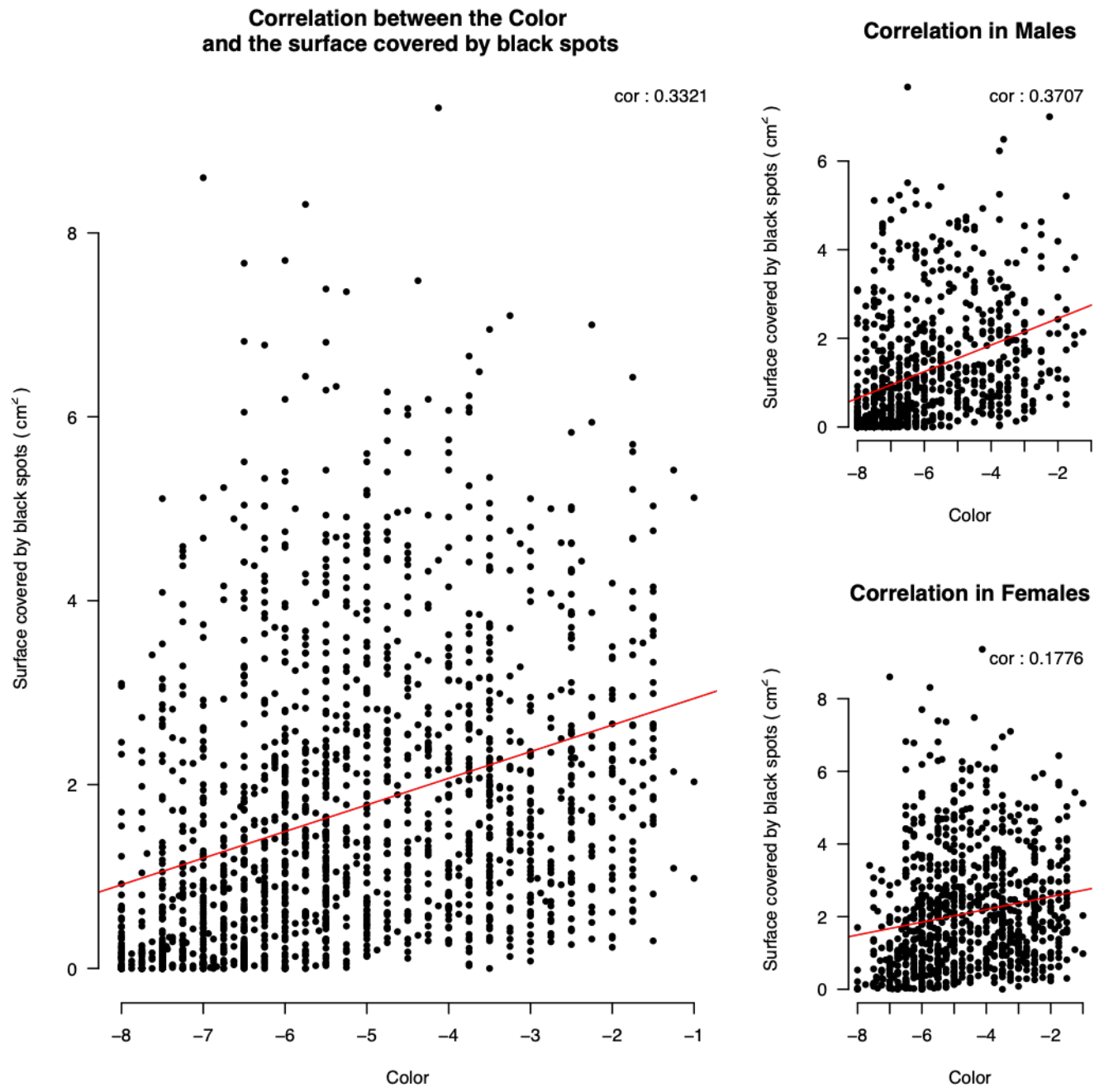

Figure S3 – Correlation between the color of all the individuals and their spottiness (left panel, n = 1916), as well as within each sex (upper right for Males (n = 864) and bottom right for Females (n = 1052)).

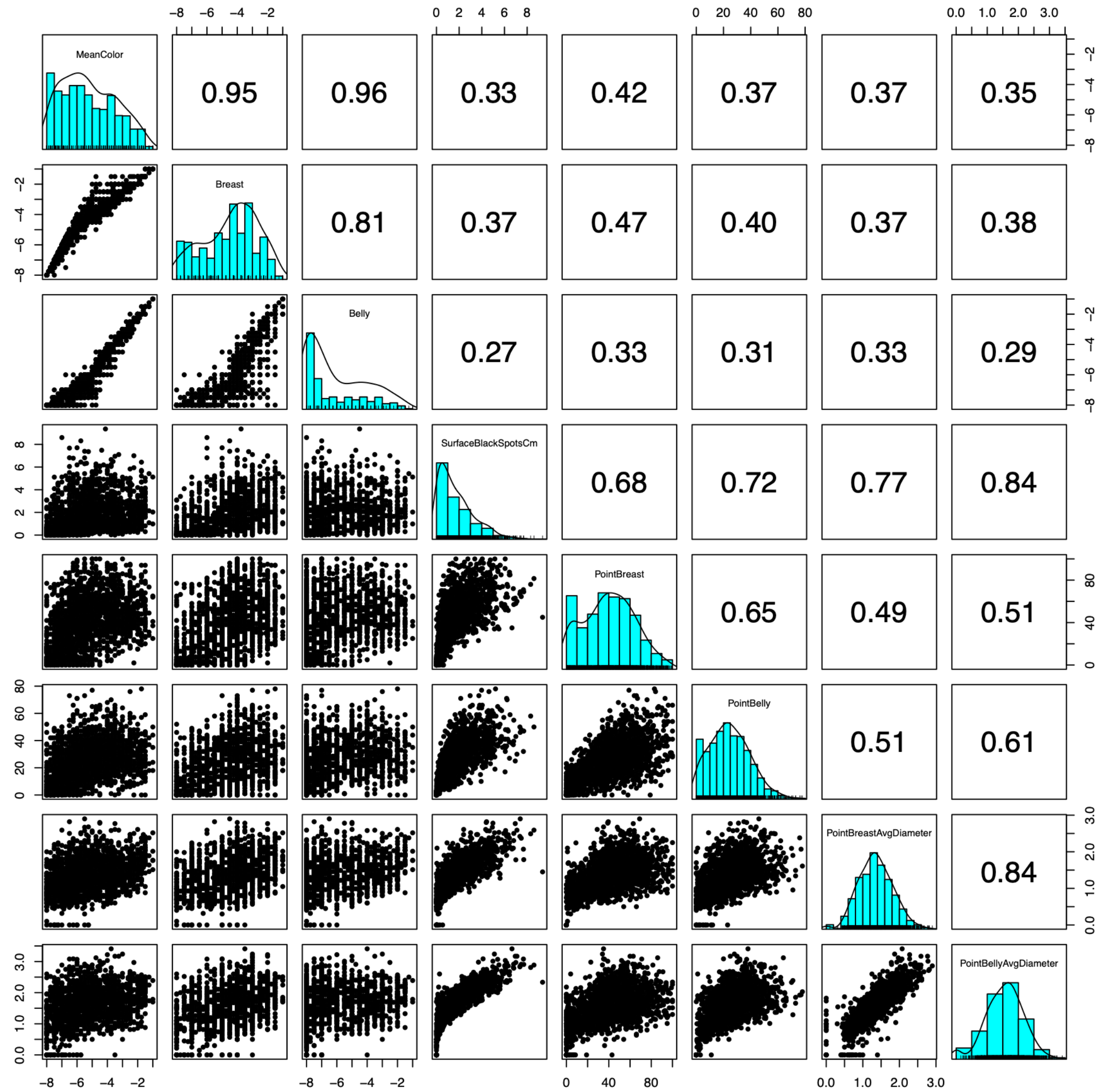

Figure S4 – Distribution and correlation between all the color and spottiness variables in all individuals (n = 8250). The three first rows and columns present the color variables (namely the "*MeanColor*" used in the main text, the "*Breast*" referring to the color of the breast of the individual and the "*belly*", referring to the color of the belly of the individuals). The next five columns and rows resent the spottiness variables (namely "*SurfaceBackSpotsCm*" used as an overall measure of the spottiness in the main text, the "*PointBreast*" and "*PointBelly*" variables describing the number of Spots counted in each part of the body, and "*PointBreastAvgDiameter*" and "*PointBellyAvgDiameter*" presenting the average diameter of the spots in each body part).

Evolution of two melanin-based traits

Table S1 – Summary of the best model explaining the evolution of the color. P-values were obtained for fixed effects using Wald tests. All tests were two-sided.

	(MeanColor)		
Predictors	*Estimates*	*CI*	*p*
(Intercept)	-87.74	-111.70 – -63.78	**7.720e-13**
HatchYear	0.04	0.03 – 0.05	**2.499e-11**
Stage [Fledgling]	0.51	0.43 – 0.60	**3.841e-35**
Sex [2]	1.37	1.30 – 1.44	**9.200e-314**
**Random Effects**			
σ^2	2.29		
$\tau_{00\ YearFirstObs}$	0.07		
ICC	0.03		
$N_{YearFirstObs}$	30		
Observations	7713		
Marginal R^2 / Conditional R^2	0.212 / 0.236		

Table S2 – Summary of the best model explaining the evolution of the spottiness. P-values were obtained for fixed effects using Wald tests. All tests were two-sided.

	**(SurfaceBlackSpotsCm)**		
Predictors	*Estimates*	*CI*	*p*
(Intercept)	-96.58	-120.56 – -72.61	**3.350e-15**
HatchYear	0.05	0.04 – 0.06	**1.595e-15**
Stage [Fledgling]	0.28	0.18 – 0.37	**6.498e-09**
Sex [2]	0.91	0.84 – 0.99	**1.925e-111**
**Random Effects**			
σ^2	2.59		
$\tau_{00\ YearFirstObs}$	0.07		
ICC	0.03		
$N_{YearFirstObs}$	30		
Observations	6542		
Marginal R^2 / Conditional R^2	0.134 / 0.156		

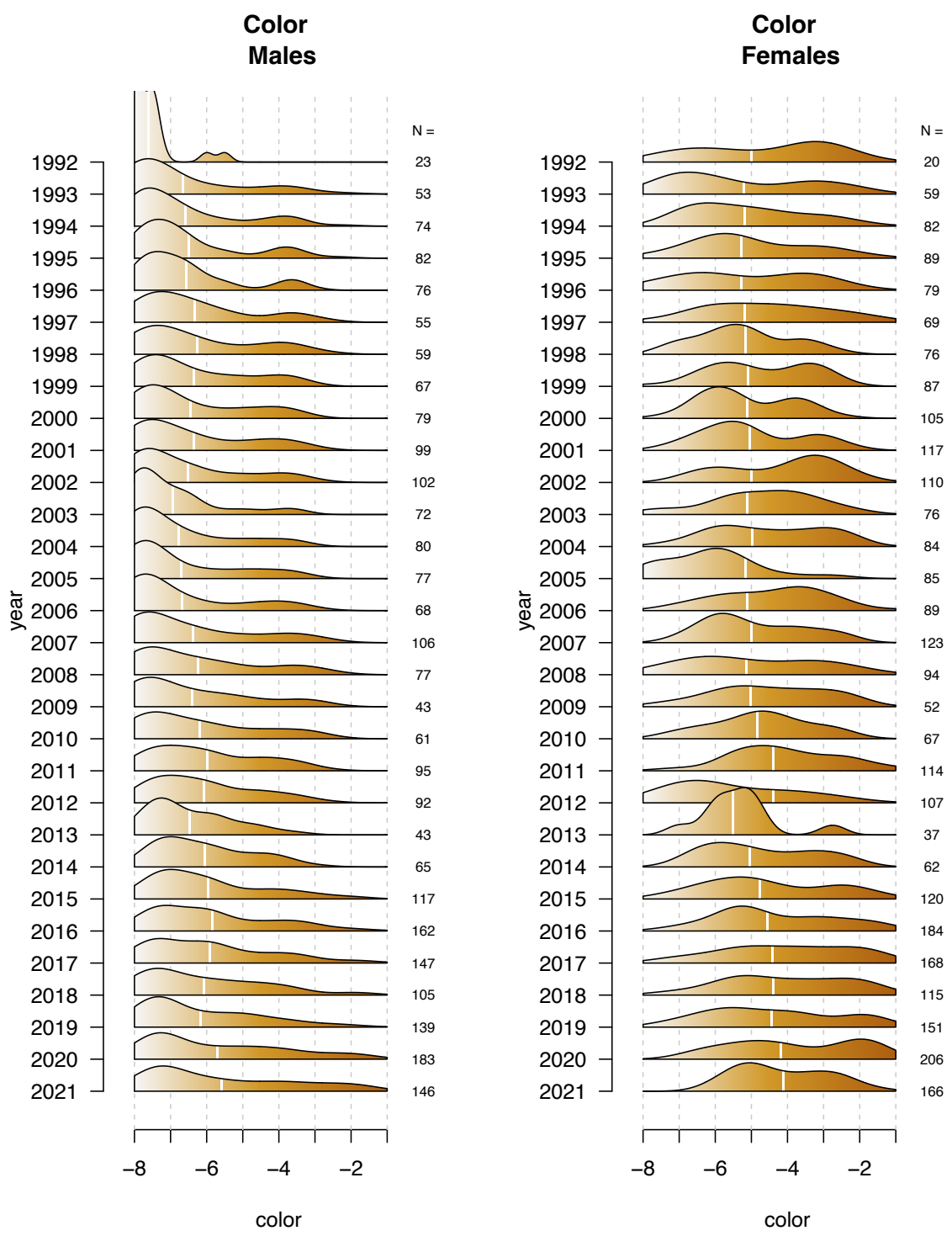

Figure S5 - Evolution of the color of the individuals along the year for males (left, n = 864) and females (right, n = 1052) based on adults alive that year. For each year, the white bar presents the mean of the distribution. Numbers present sample size for each year.

Table S3 – Summary of the model explaining the evolution of the color in males only. P-values were obtained for fixed effects using Wald tests. All tests were two-sided.

		(MeanColor)	
Predictors	*Estimates*	*CI*	*p*
(Intercept)	-92.24	-123.33 – -61.15	**6.584e-09**
HatchYear	0.04	0.03 – 0.06	**5.902e-08**
Stage [Fledgling]	0.60	0.47 – 0.74	**1.094e-17**
**Random Effects**			
σ^2	2.62		
$\tau_{00\ YearFirstObs}$	0.11		
ICC	0.04		
$N_{YearFirstObs}$	30		
Observations	3106		
Marginal R^2 / Conditional R^2	0.076 / 0.112		

Table S4 – Summary of the model explaining the evolution of the color in females only. P-values were obtained for fixed effects using Wald tests. All tests were two-sided.

		(MeanColor)	
Predictors	*Estimates*	*CI*	*p*
(Intercept)	-86.95	-111.80 – -62.10	**8.123e-12**
HatchYear	0.04	0.03 – 0.05	**9.918e-11**
Stage [Fledgling]	0.51	0.40 – 0.62	**2.792e-19**
**Random Effects**			
σ^2	2.00		
$\tau_{00\ YearFirstObs}$	0.07		
ICC	0.03		
$N_{YearFirstObs}$	30		
Observations	3432		
Marginal R^2 / Conditional R^2	0.088 / 0.117		

Table S5 – Summary of the model explaining the evolution of the spottiness in males only. P-values were obtained for fixed effects using Wald tests. All tests were two-sided.

	(SurfaceBlackSpotsCm)		
Predictors	*Estimates*	*CI*	*p*
(Intercept)	-111.01	-137.18 – -84.84	**1.319e-16**
HatchYear	0.06	0.04 – 0.07	**6.284e-17**
Stage [Fledgling]	0.23	0.10 – 0.35	**2.605e-04**
**Random Effects**			
σ^2	2.07		
$\tau_{00\ YearFirstObs}$	0.07		
ICC	0.03		
$N_{YearFirstObs}$	30		
Observations	3107		
Marginal R^2 / Conditional R^2	0.105 / 0.135		

Table S6 – Summary of the model explaining the evolution of the spottiness in females only. P-values were obtained for fixed effects using Wald tests. All tests were two-sided.

	(SurfaceBlackSpotsCm)		
Predictors	*Estimates*	*CI*	*p*
(Intercept)	-87.93	-116.78 – -59.09	**2.501e-09**
HatchYear	0.04	0.03 – 0.06	**1.085e-09**
Stage [Fledgling]	0.32	0.19 – 0.46	**2.638e-06**
**Random Effects**			
σ^2	3.03		
$\tau_{00\ YearFirstObs}$	0.09		
ICC	0.03		
$N_{YearFirstObs}$	30		
Observations	3435		
Marginal R^2 / Conditional R^2	0.057 / 0.083		

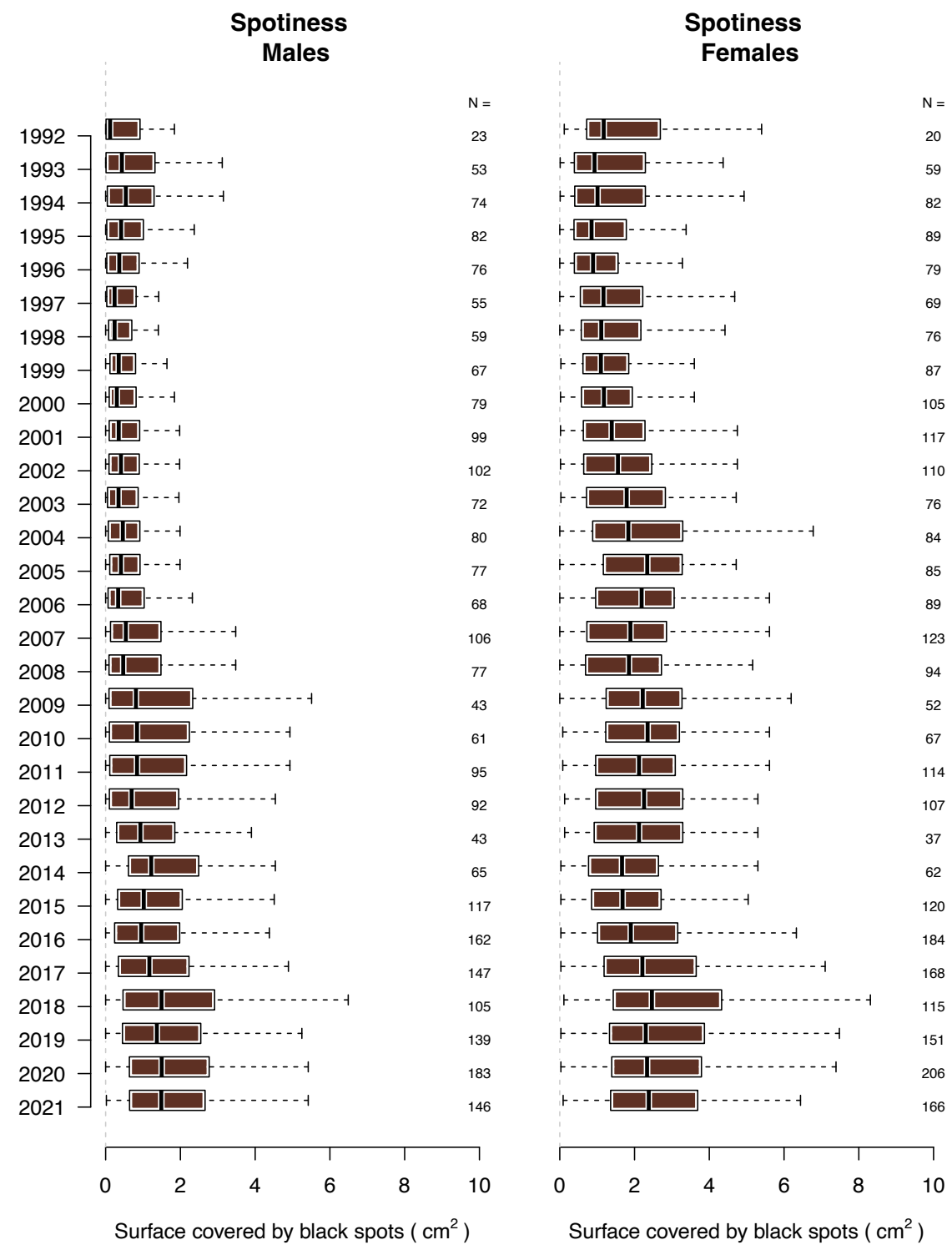

Figure S6 - Evolution of the spottiness of the individuals along the year for males ((left, n = 864) and females (right, n = 1052) based on adults alive that year. Box plots show the median (centre line), the first and third quartiles (box bounds), and whiskers extending to the most extreme data points within 1.5 × the interquartile range (IQR) from the box. Points beyond the whiskers are shown as outliers.

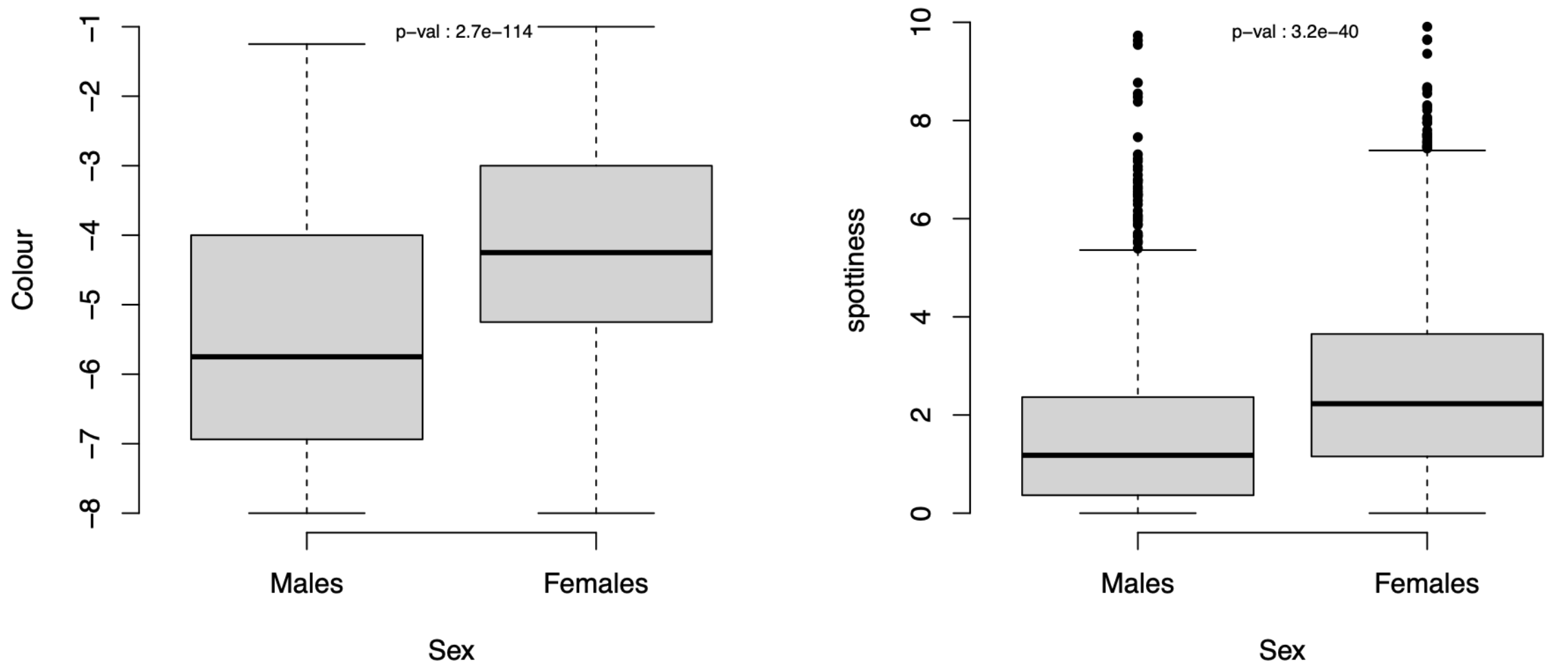

Figure S7 – Sexual dimorphism in the color (left panel) and number of spots (right panel) between males (n = 3722) and females (n = 4059) in the barn owl. Box plots show the median (centre line), the first and third quartiles (box bounds), and whiskers extending to the most extreme data points within 1.5 × the interquartile range (IQR) from the box. Points beyond the whiskers are shown as outliers. P-values on top of each plot present the result of the Wilcoxon Rank Sum test comparing both distributions.

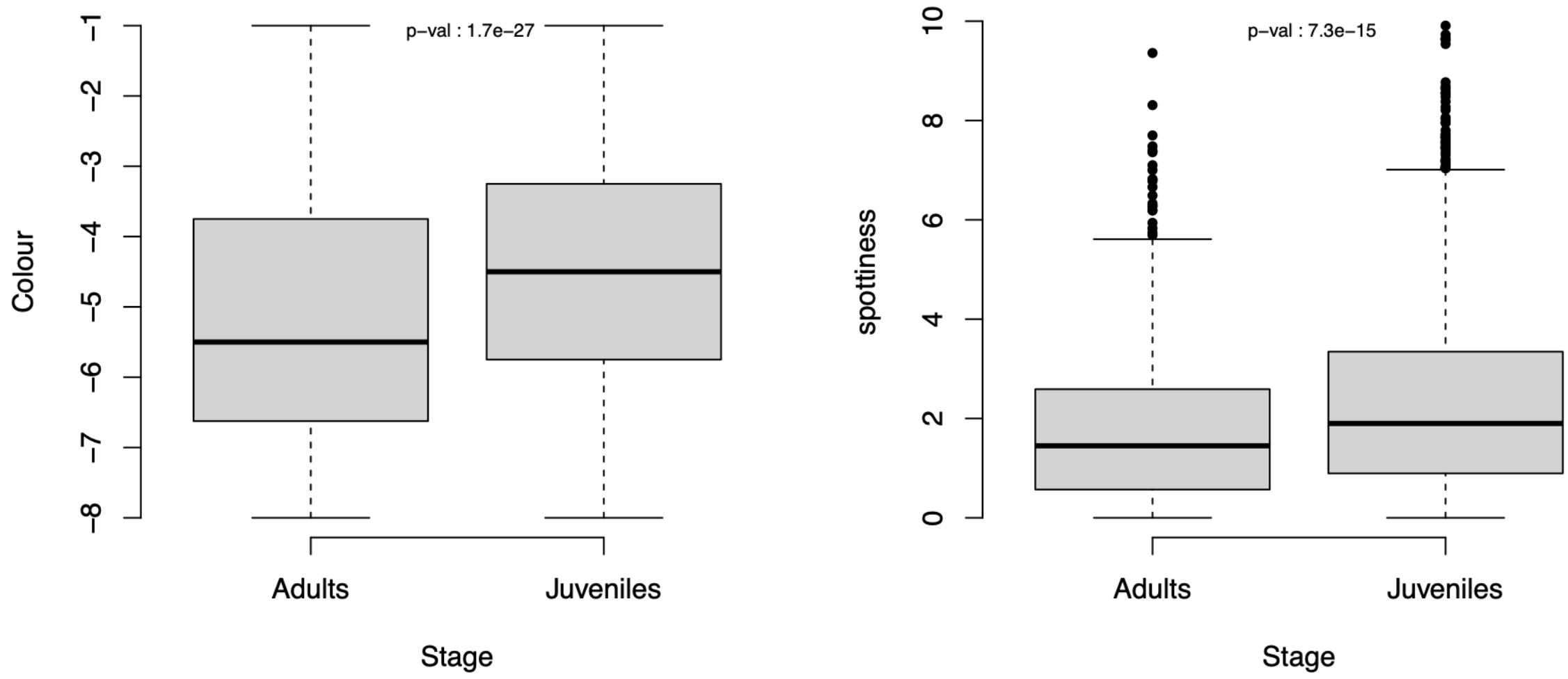

Figure S8 – Difference between juveniles (n = 6160) and adults (n = 2090) in the color (left panel) and spottiness (right panel) in the barn owl. Box plots show the median (centre line), the first and third quartiles (box bounds), and whiskers extending to the most extreme data points within 1.5 × the interquartile range (IQR) from the box. Points beyond the whiskers are shown as outliers. P-values on top of each plot present the result of the Wilcoxon Rank Sum test comparing both distributions.

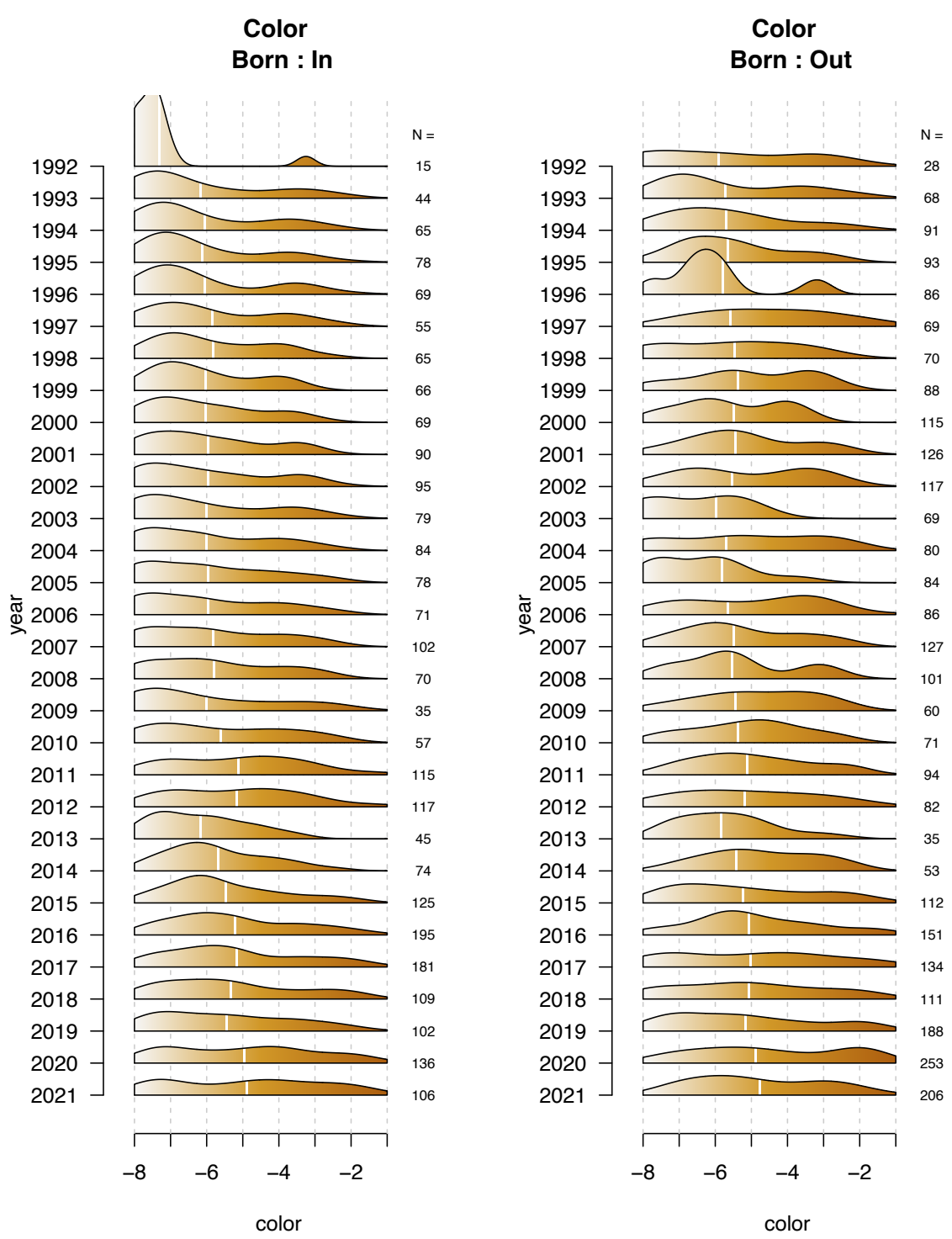

Figure S9 – Evolution of the color of the individuals along the year for individuals born in the system (i.e. ringed as fledglings - left panel, n = 837) or outside the system (i.e. ringed as adults - left panel, n = 1079). Distributions are based on individuals alive that year.

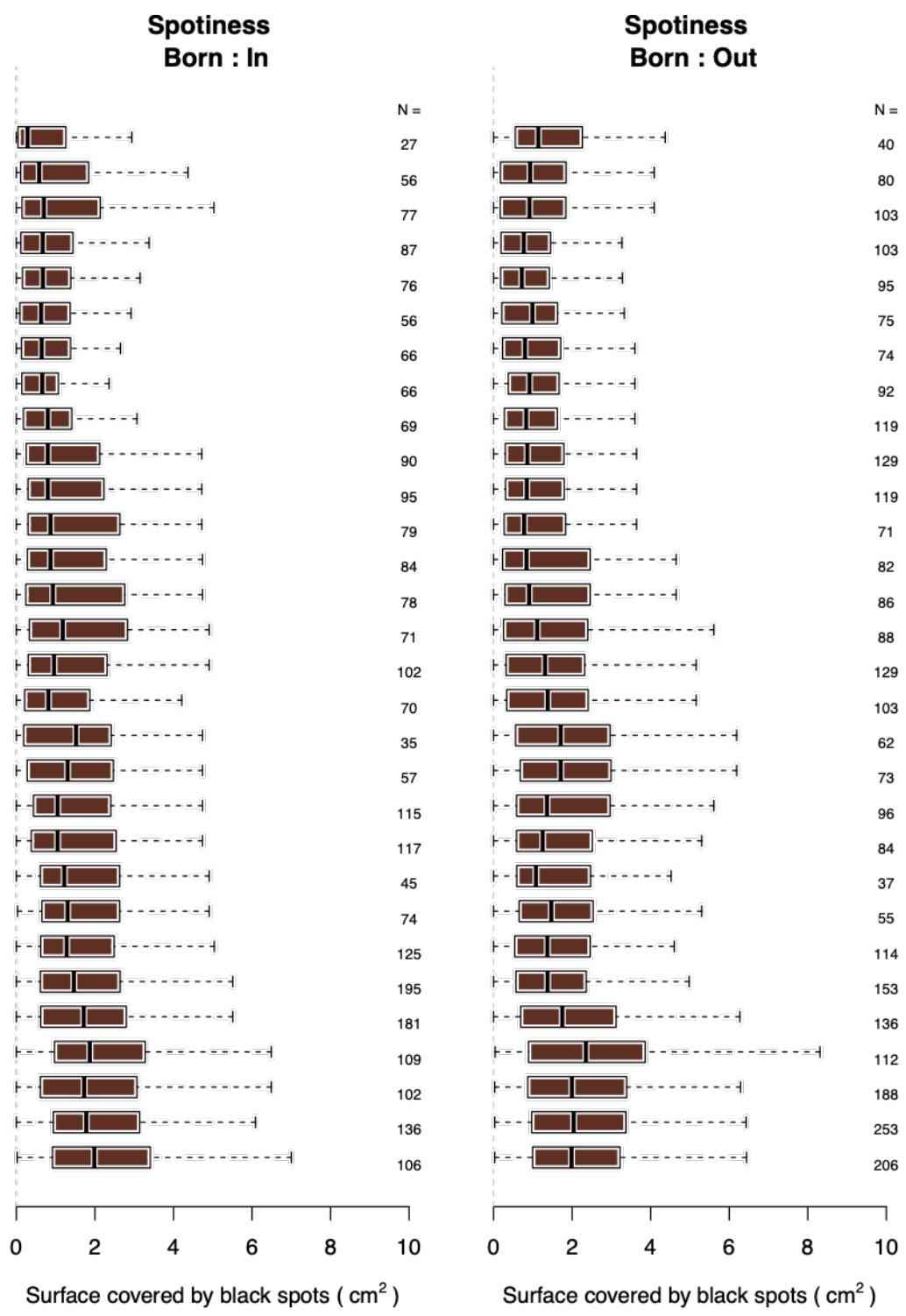

Figure S10 - Evolution of the Spottiness of the individuals along the year for individuals born in the system (i.e. ringed as fledgelings - left panel, n = 837) or outside the system (i.e. ringed as adults - left panel, n = 1079). Distributions are based on individuals alive that year.

Table S7 – Summary of the best model explaining the evolution of the color, excluding the first five years of the study period to avoid potential bias due to low sample size (from 1998). P-values were obtained for fixed effects using Wald tests. All tests were two-sided.

	(MeanColor)		
Predictors	*Estimates*	*CI*	*p*
(Intercept)	-84.17	-109.58 – -58.76	**8.906e-11**
HatchYear	0.04	0.03 – 0.05	**1.710e-09**
Stage [Fledgling]	0.51	0.42 – 0.59	**2.995e-32**
Sex [2]	1.39	1.32 – 1.46	**6.036e-296**
**Random Effects**			
σ^2	2.29		
$\tau_{00\ YearFirstObs}$	0.07		
ICC	0.03		
$N_{YearFirstObs}$	26		
Observations	7148		
Marginal R^2 / Conditional R^2	0.202 / 0.225		

Table S8 – Summary of the best model explaining the evolution of the spottiness, excluding the first five years of the study period to avoid potential bias due to low sample size (from 1998). P-values were obtained for fixed effects using Wald tests. All tests were two-sided.

	(SurfaceBlackSpotsCm)		
Predictors	*Estimates*	*CI*	*p*
(Intercept)	-105.29	-131.98 – -78.60	**1.212e-14**
HatchYear	0.05	0.04 – 0.07	**6.026e-15**
Stage [Fledgling]	0.27	0.17 – 0.36	**1.103e-07**
Sex [2]	0.92	0.84 – 1.01	**1.417e-99**
**Random Effects**			
σ^2	2.69		
$\tau_{00\ YearFirstObs}$	0.07		
ICC	0.02		
$N_{YearFirstObs}$	26		
Observations	5982		
Marginal R^2 / Conditional R^2	0.125 / 0.146		

Population-wide whole genome sequencing of the Swiss population

Genetic diversity in reference panel

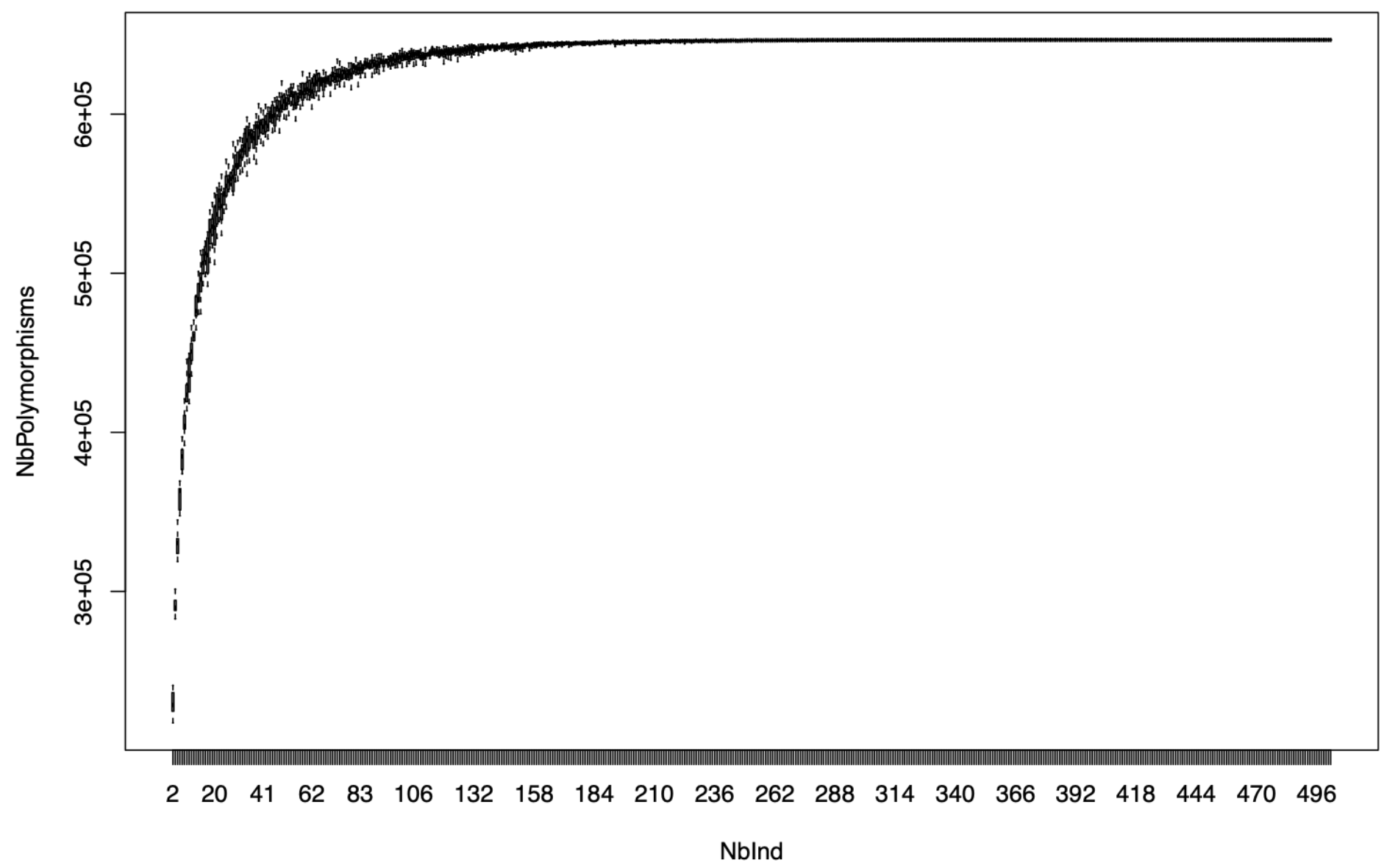

Figure S11 – Relation between the number of individuals composing the reference panel and the number of variants identified. Each boxplot presents the results for ten random draws of *x* individuals, for which we recorded the number of polymorphic variant (each allele was present at least one time). With a sample size of ~180 and more individuals, the number of variants does not increase anymore, thus implying that the reference panel capture well the diversity of frequent alleles (see M&M section for filtering details).

Phasing of the reference panel

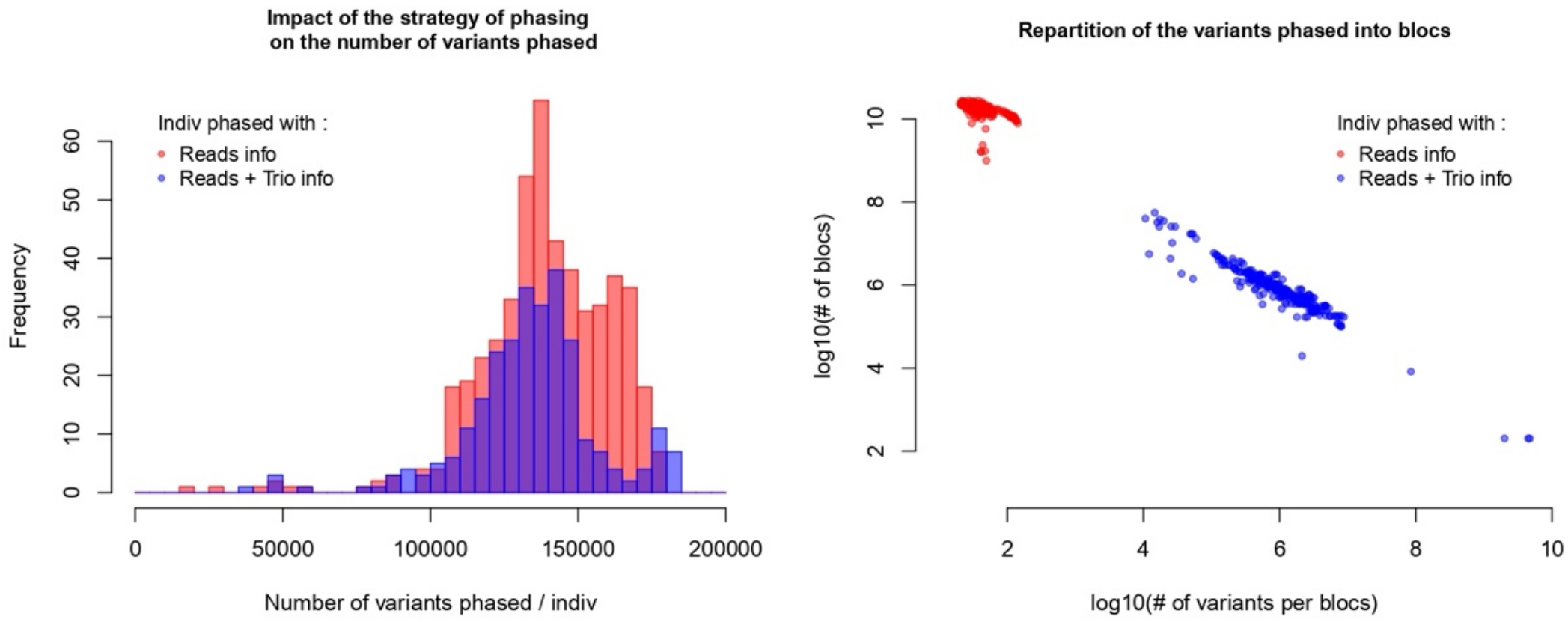

Figure S12 – results of the read-based phasing (using WhatsHap) of the reference panel. The left panel depict the number of variants phased per individuals, with in red individuals phased only with their read information and in blue the individuals with a phasing done using both reads and trio information (see M&M section for details). Both strategies perform equally, phasing a high number of variants per individuals. The right panel present the relation between the number of blocs of variants phased in the same individuals and the size of the number of variants par blocs. The Color code is the same as before, based on the phasing data available. Individuals phased only with the reads have a higher number of blocs with less variants per blocs relative to the individuals phased taking advantage of the trio information on top of the reads.

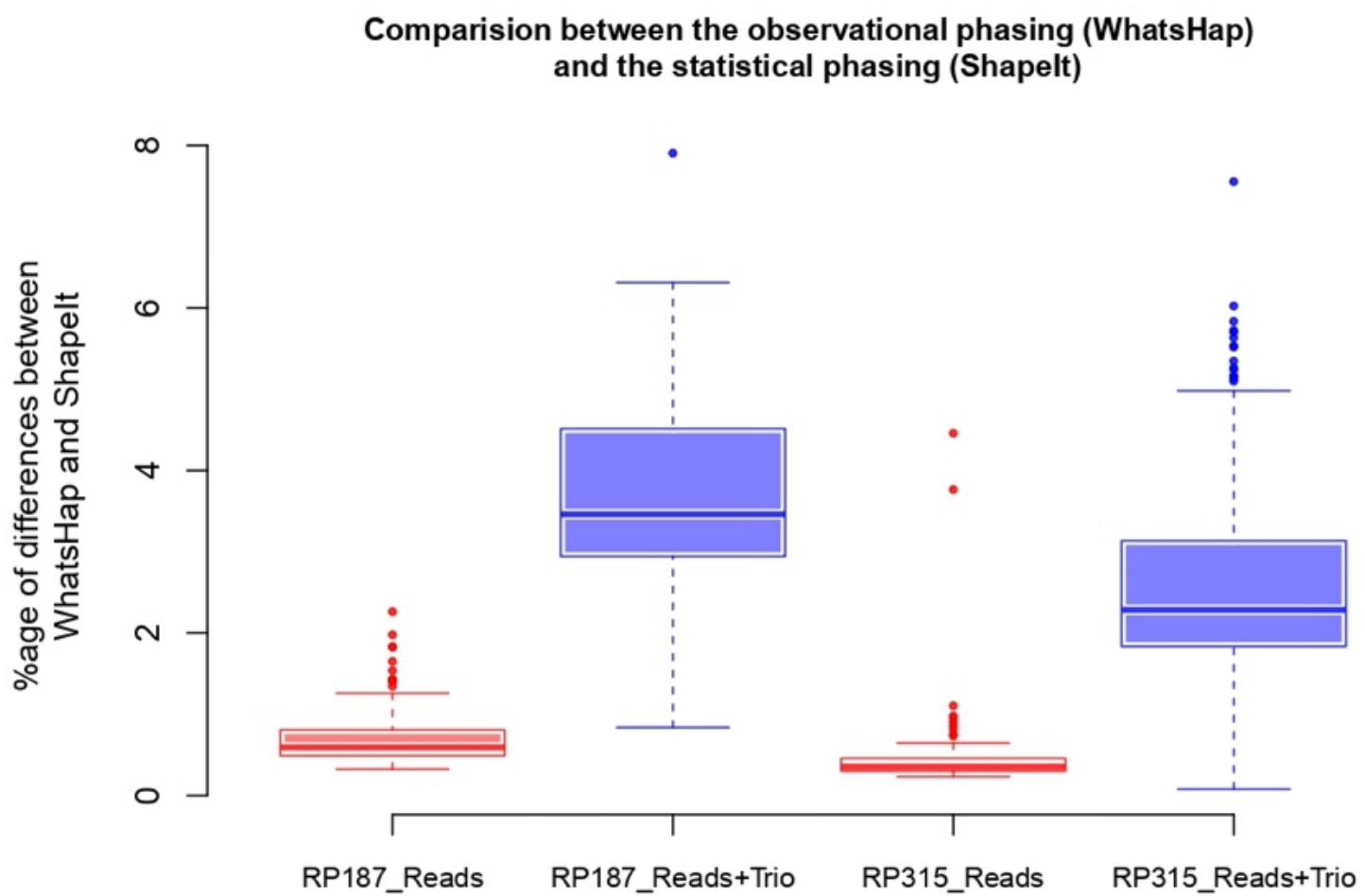

Figure S13 – Boxplots presenting the percentage of differences between the phasing based on the reads (and trio) information and the statistical phasing. Boxplots show the median (centre line), the first and third quartiles (box bounds), and whiskers extending to the most extreme data points within 1.5 × the interquartile range (IQR) from the box. Points beyond the whiskers are shown as outliers. Each value of the boxplot represents the differences between two independent phasing of the same individual, one with the read based approach only and with the statistical approach only. Individuals

phased with the trio information have longer blocs with more variants, thus increasing the proportion of blocs with errors compare to individuals phased only with reads.

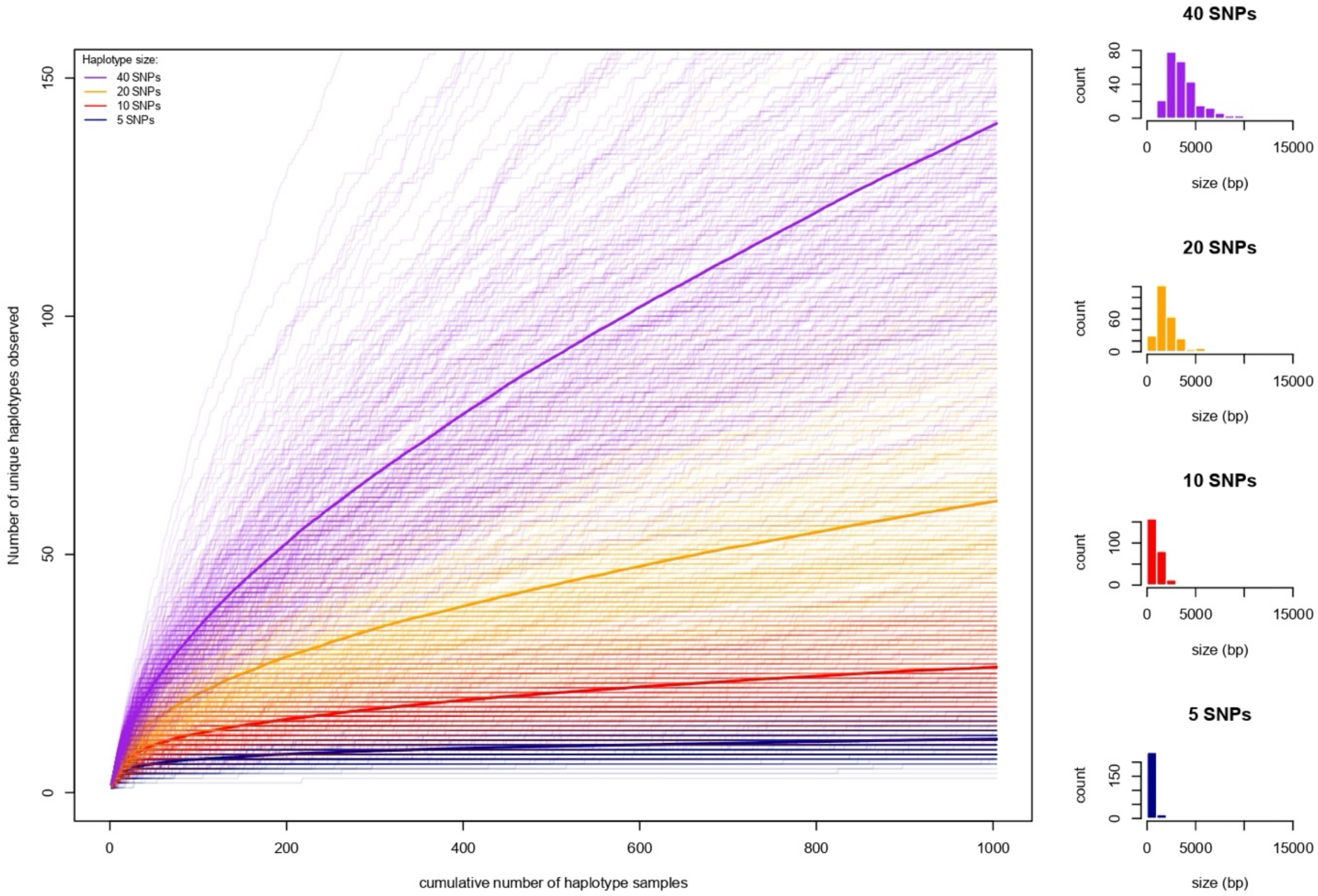

Figure S14 - Relation between the number of local haplotypes composing the reference panel and the number of unique haplotypes for four different haplotype size (5 SNPs, 10 SNPs, 20 SNPs and 40 SNPs). Each line represents the cumulative number of unique haplotypes within one random subsampling of all haplotypes present in each region. Regions were selected randomly along the genome. Solid lines represent the mean cumulative number of haplotypes over 200 random regions. The right panel present the distribution of the sizes of the regions randomly selected and presented in the left panel.

Low coverage sequencing of 2778 owls

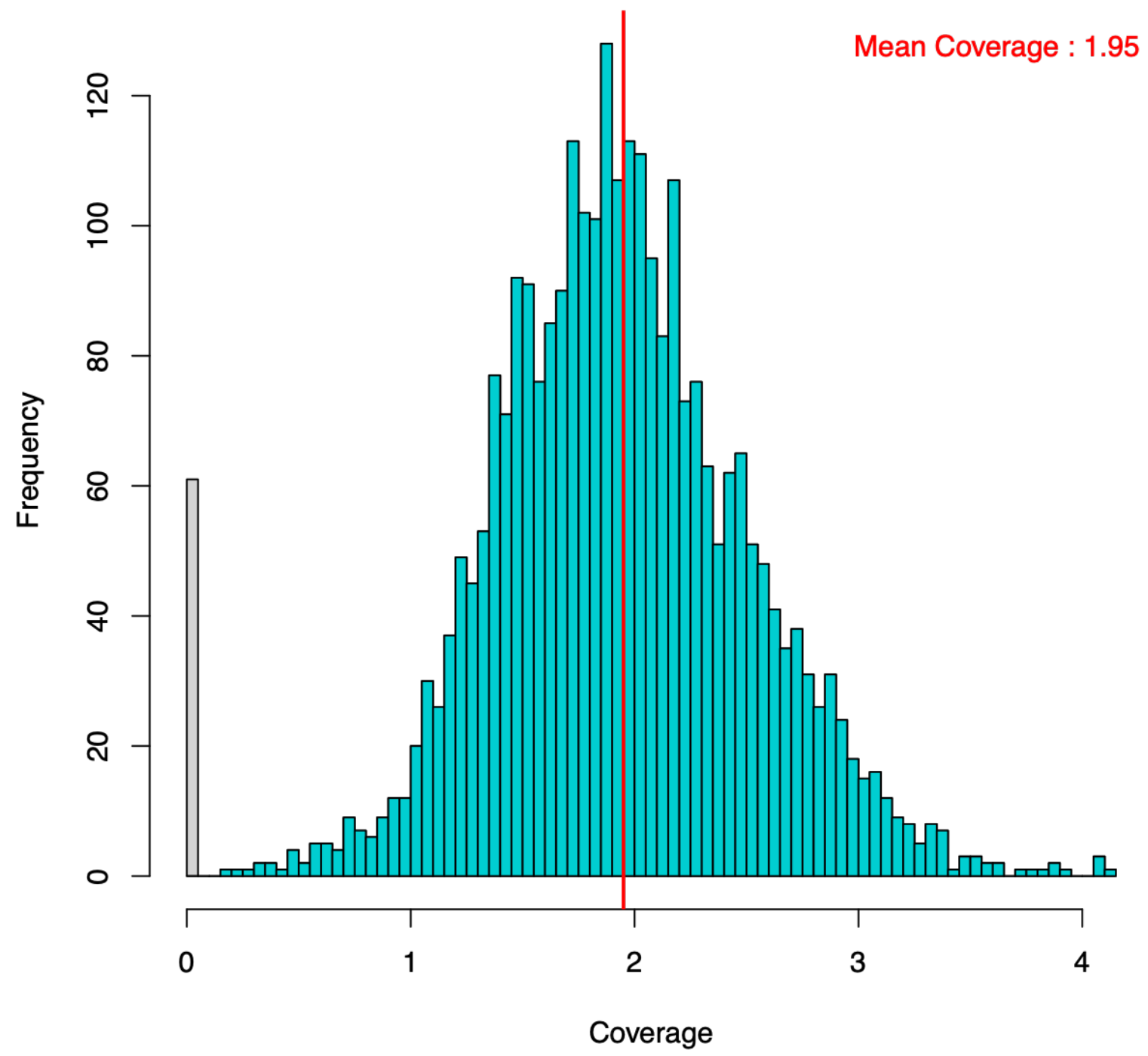

Figure S15 – Depth of coverage of the 2778 owls sequenced at low coverage. The grey at 0 represent the coverage of the control wells used during sequencing (see M&M section for details). Turquoise distribution represents the coverage of the owls. Red vertical line depicts the mean value of the distribution at 1.95X. Minimum coverage: 0.2X. Maximum Coverage: 4.15X.

<u>Heritability of the coloration and the Spottiness</u>

Table S9 - Summary of the model estimating the heritability of the coloration. The model included StageBin and SexWGS as fixed effects and additive genetic effects modelled using the genomic relatedness matrix (GRM). Values are posterior means with 95% credible intervals (CI). Estimates were obtained from four Markov chain Monte Carlo chains (12,000 post-warmup samples).

Family: gaussian
Links: mu = identity; sigma = identity
Formula: MeanColor ~ 1 + as.factor(StageBin) + as.factor(SexWGS) + (1 | gr(ID, cov = grm))
Data: TabSummary (Number of observations: 3083)
Draws: 4 chains, each with iter = 5000; warmup = 2000; thin = 1; total post-warmup draws = 12000

Group-Level Effects: ~ID (Number of levels: 3083)

	Estimate	Est.Error	l-95% CI	u-95% CI	Rhat	Bulk_ESS	Tail_ESS
sd(Intercept)	1.81	0.06	1.70	1.93	1.01	784	1743

Population-Level Effects:

	Estimate	Est.Error	l-95% CI	u-95% CI	Rhat	Bulk_ESS	Tail_ESS
Intercept	-5.79	0.10	-5.98	-5.60	1.00	5037	6394
as.factorStageBin2	0.73	0.04	0.64	0.81	1.00	9996	9728
as.factorSexWGS2	1.24	0.12	1.00	1.48	1.00	4793	6197

Family Specific Parameters:

	Estimate	Est.Error	l-95% CI	u-95% CI	Rhat	Bulk_ESS	Tail_ESS
sigma	0.86	0.03	0.79	0.92	1.00	709	1389

Table S10 – Summary of the model estimating the heritability of the spottiness. The model included Stage and Sex as fixed effects and additive genetic effects modelled using the genomic relatedness matrix (GRM). Values are posterior means with 95% credible intervals (CI). Estimates were obtained from four Markov chain Monte Carlo chains (12,000 post-warmup samples).

Family: gaussian
Links: mu = identity; sigma = identity
Formula: SurfaceBlackSpotsCm ~ 1 + as.factor(Stage) + as.factor(Sex) + (1 | gr(ID, cov = grm))
Draws: 4 chains, each with iter = 5000; warmup = 2000; thin = 1; total post-warmup draws = 12000

Group-Level Effects:
~ID (Number of levels: 2480)

	Estimate	Est.Error	l-95% CI	u-95% CI	Rhat	Bulk_ESS	Tail_ESS
sd(Intercept)	1.97	0.07	1.82	2.10	1.01	848	2175

Population-Level Effects:

	Estimate	Est.Error	l-95% CI	u-95% CI	Rhat	Bulk_ESS	Tail_ESS
Intercept	1.24	0.11	1.02	1.46	1.00	3612	5603
as.factorStageBin2	0.47	0.06	0.36	0.58	1.00	7582	8424
as.factorSexWGS2	1.03	0.14	0.76	1.29	1.00	3678	5746

Family Specific Parameters:

	Estimate	Est.Error	l-95% CI	u-95% CI	Rhat	Bulk_ESS	Tail_ESS
sigma	0.99	0.04	0.91	1.06	1.01	759	1937

Genome Wide Association Study

GWAS – Plumage coloration

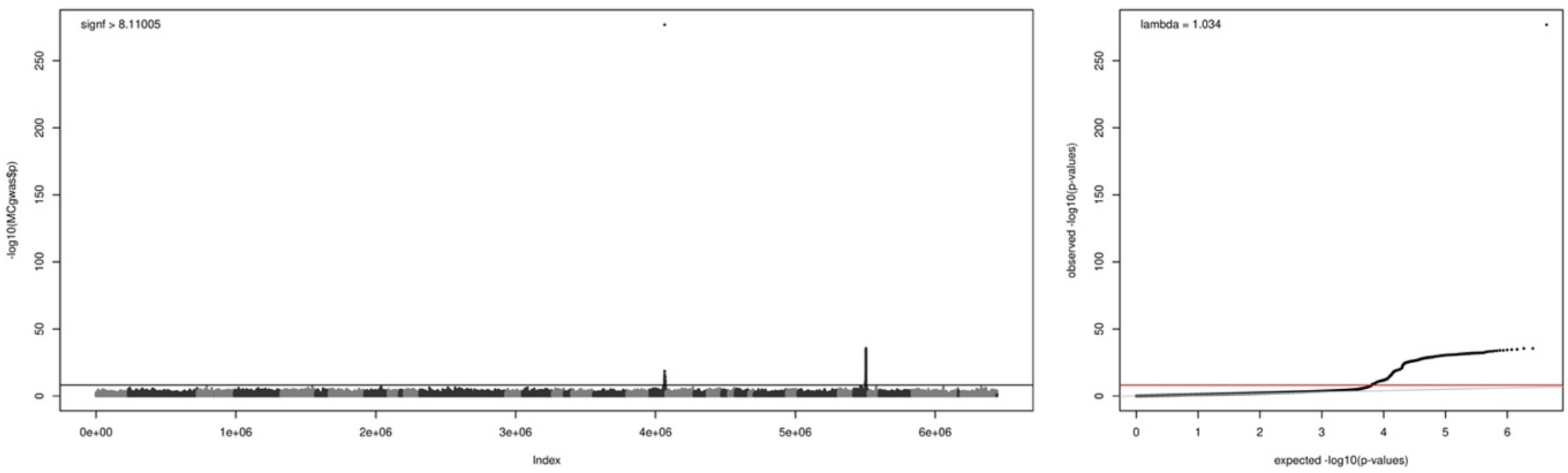

Figure S16 – Genome Wide Association (GWA) study between the genotypes and the **plumage coloration of the entire ventral part**. Left panel: Association scores (-log_{10}[p-values]) of each SNPs with the trait. Alternated colors depict the successive super-scaffolds (SC_) in the genome. Horizontal line presents the significance threshold according to Bonferroni. Right panel: QQ-plot of the GWAS presented on the left panel. Solid grey line represents the 1:1 line.

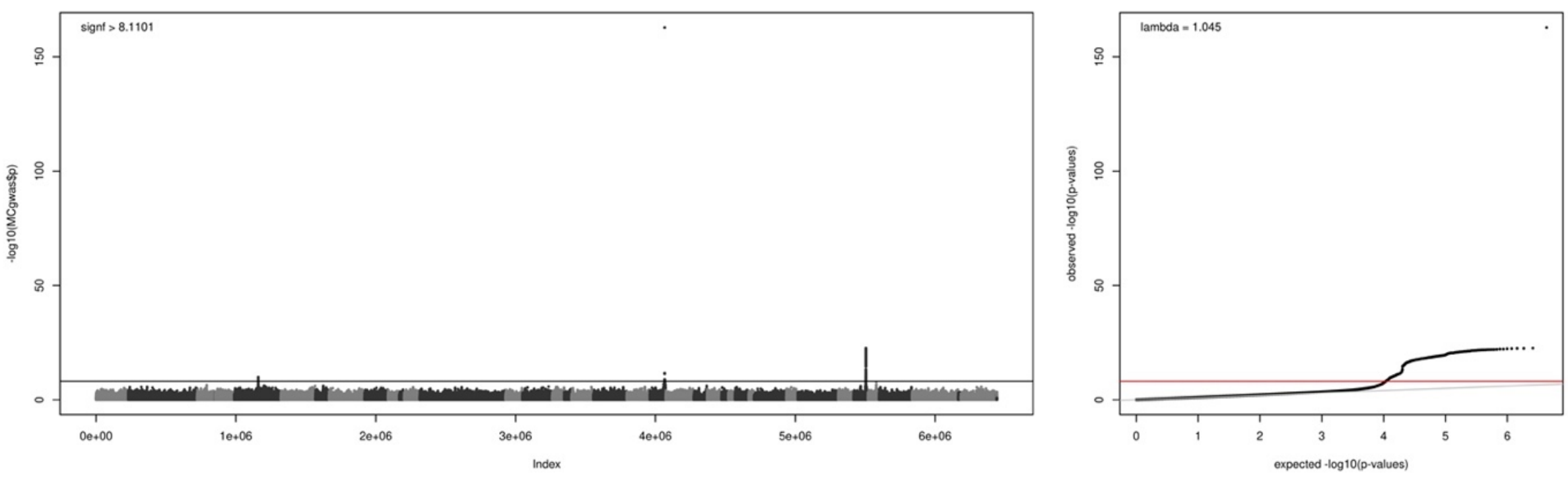

Figure S17 – Genome Wide Association (GWA) study between the genotypes and the **plumage coloration of the breast**. Left panel: Association scores (-log_{10}[p-values]) of each SNPs with the trait. Alternated colors depict the successive super-scaffolds (SC_) in the genome. Horizontal line presents the significance threshold according to Bonferroni. Right panel: QQ-plot of the GWAS presented on the left panel. Solid grey line represents the 1:1 line.

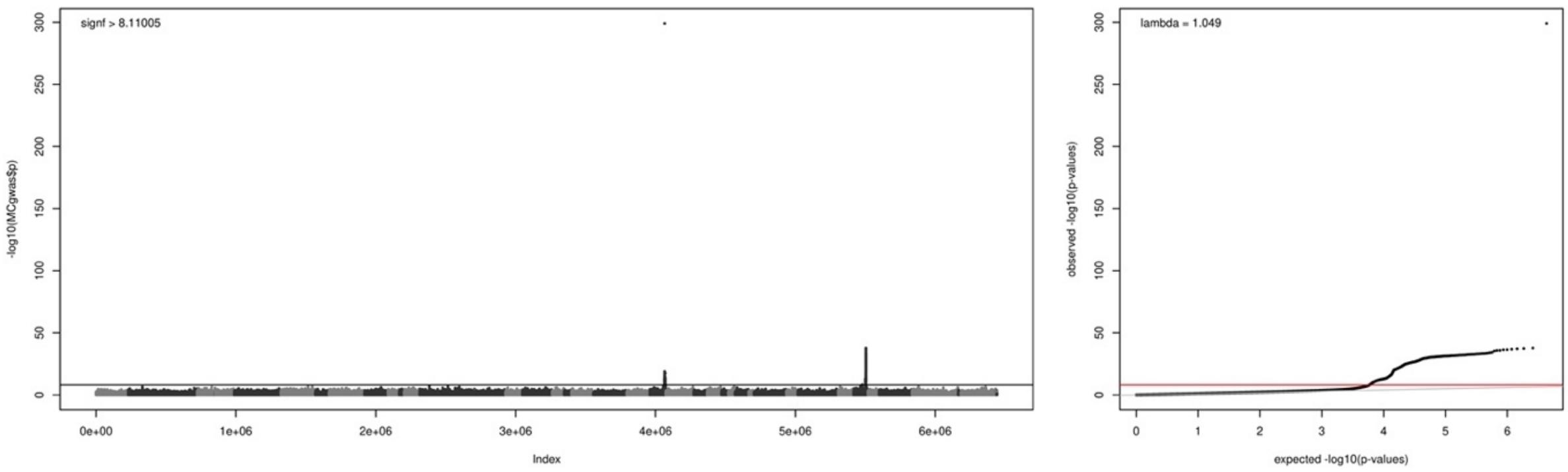

Figure S18 – Genome Wide Association (GWA) study between the genotypes and the **plumage coloration of the belly**. Left panel: Association scores (-$\log_{10}$[p-values]) of each SNPs with the trait. Alternated colors depict the successive super-scaffolds (SC_) in the genome. Horizontal line presents the significance threshold according to Bonferroni. Right panel: QQ-plot of the GWAS presented on the left panel. Solid grey line represents the 1:1 line.

GWAS – ventral spottiness

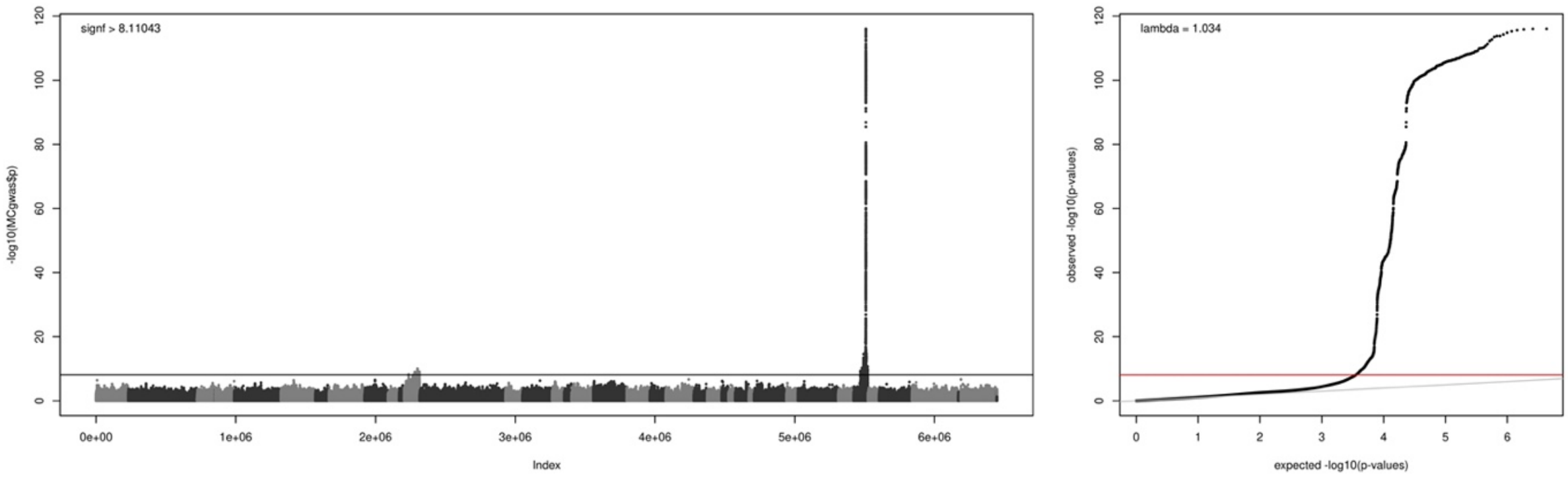

Figure S19 – Genome Wide Association (GWA) study between the genotypes and the **ventral spottiness**. Left panel: Association scores (-$\log_{10}$[p-values]) of each SNPs with the trait. Alternated colors depict the successive super-scaffolds (SC_) in the genome. Horizontal line presents the significance threshold according to Bonferroni. Right panel: QQ-plot of the GWAS presented on the left panel. Solid grey line represents the 1:1 line.

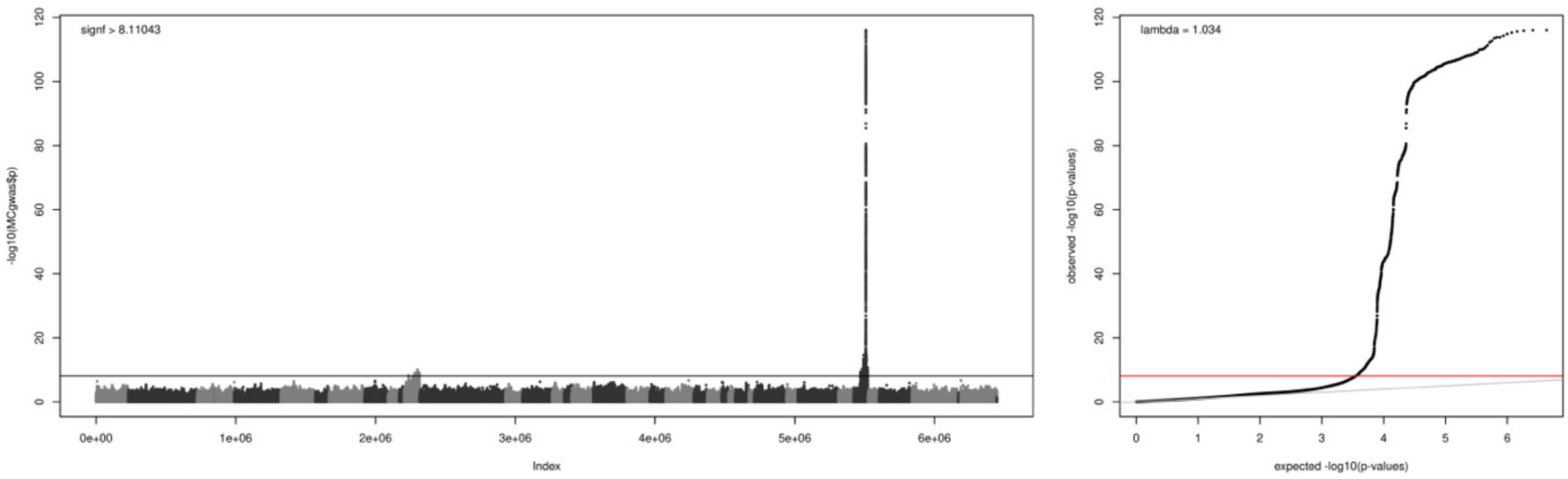

Figure S20 – Genome Wide Association (GWA) study between the genotypes and the mean **diameter of the spots**. Left panel: Association scores (-$\log_{10}$[p-values]) of each SNPs with the trait. Alternated colors depict the successive super-scaffolds (SC_) in the genome. Horizontal line presents the significance threshold according to Bonferroni. Right panel: QQ-plot of the GWAS presented on the left panel. Solid grey line represents the 1:1 line.

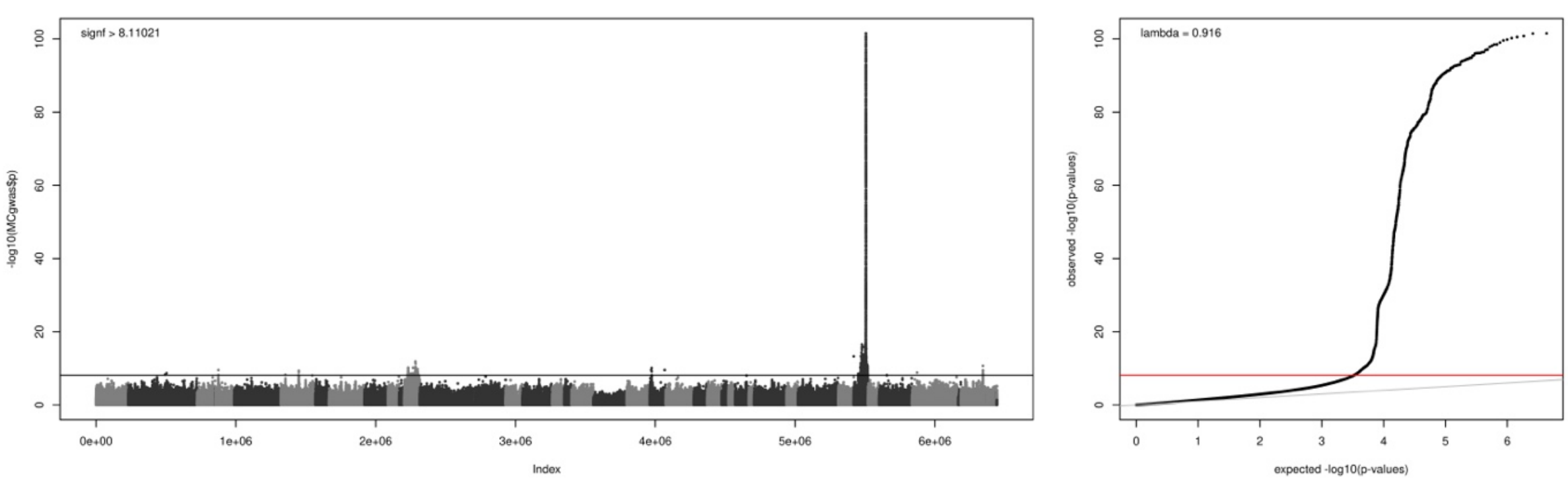

Figure S21 – Genome Wide Association (GWA) study between the genotypes and the **number of spots**. Left panel: Association scores (-$\log_{10}$[p-values]) of each SNPs with the trait. Alternated colors depict the successive super-scaffolds (SC_) in the genome. Horizontal line presents the significance threshold according to Bonferroni. Right panel: QQ-plot of the GWAS presented on the left panel. Solid grey line represents the 1:1 line.

Differential gene expression links genotype to phenotype

Genotype, gene expression and phenotype

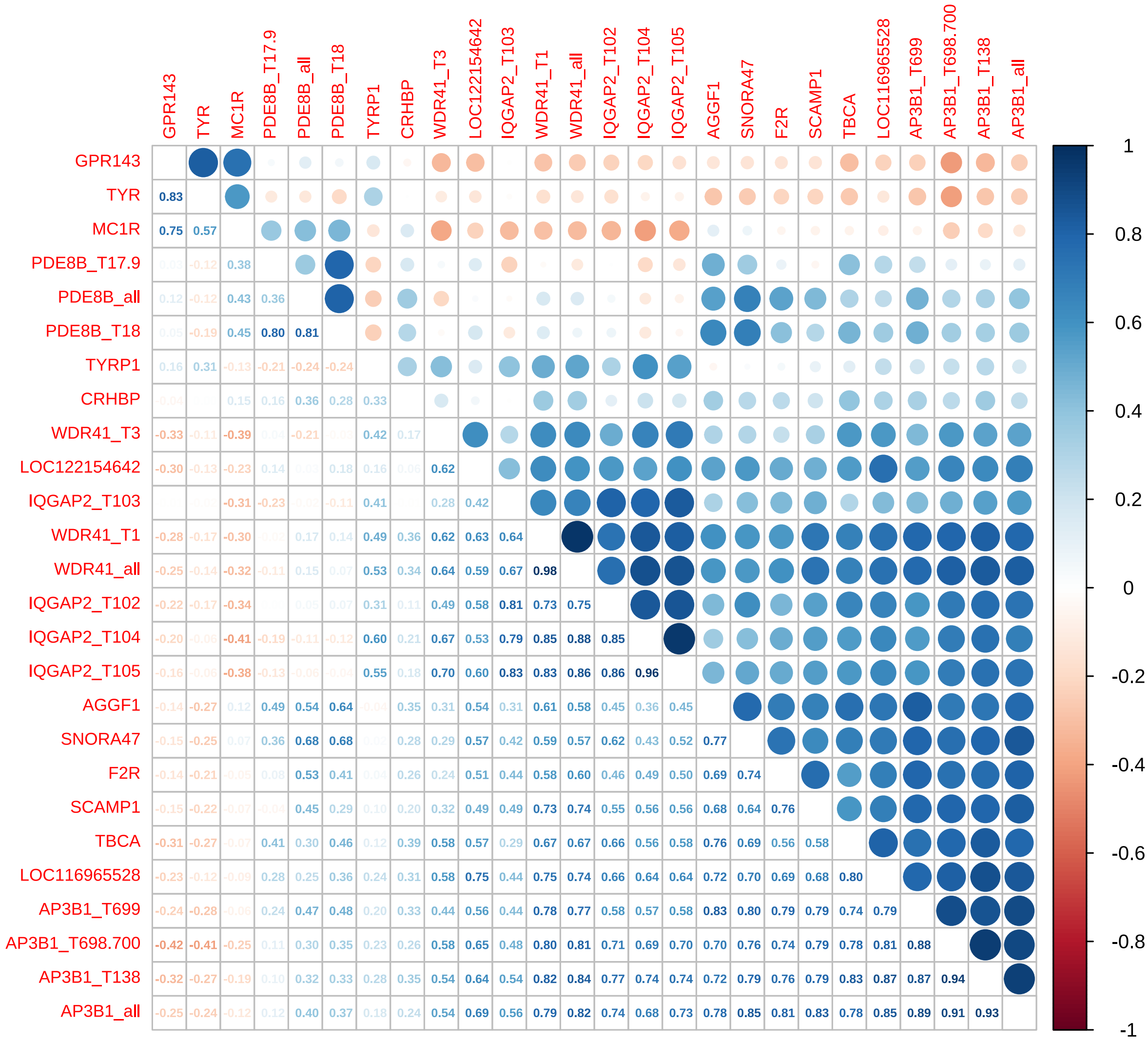

Figure S22 - Pearson's correlation between the relative normalised sqrt-root expression of all the transcripts of the tested genes.

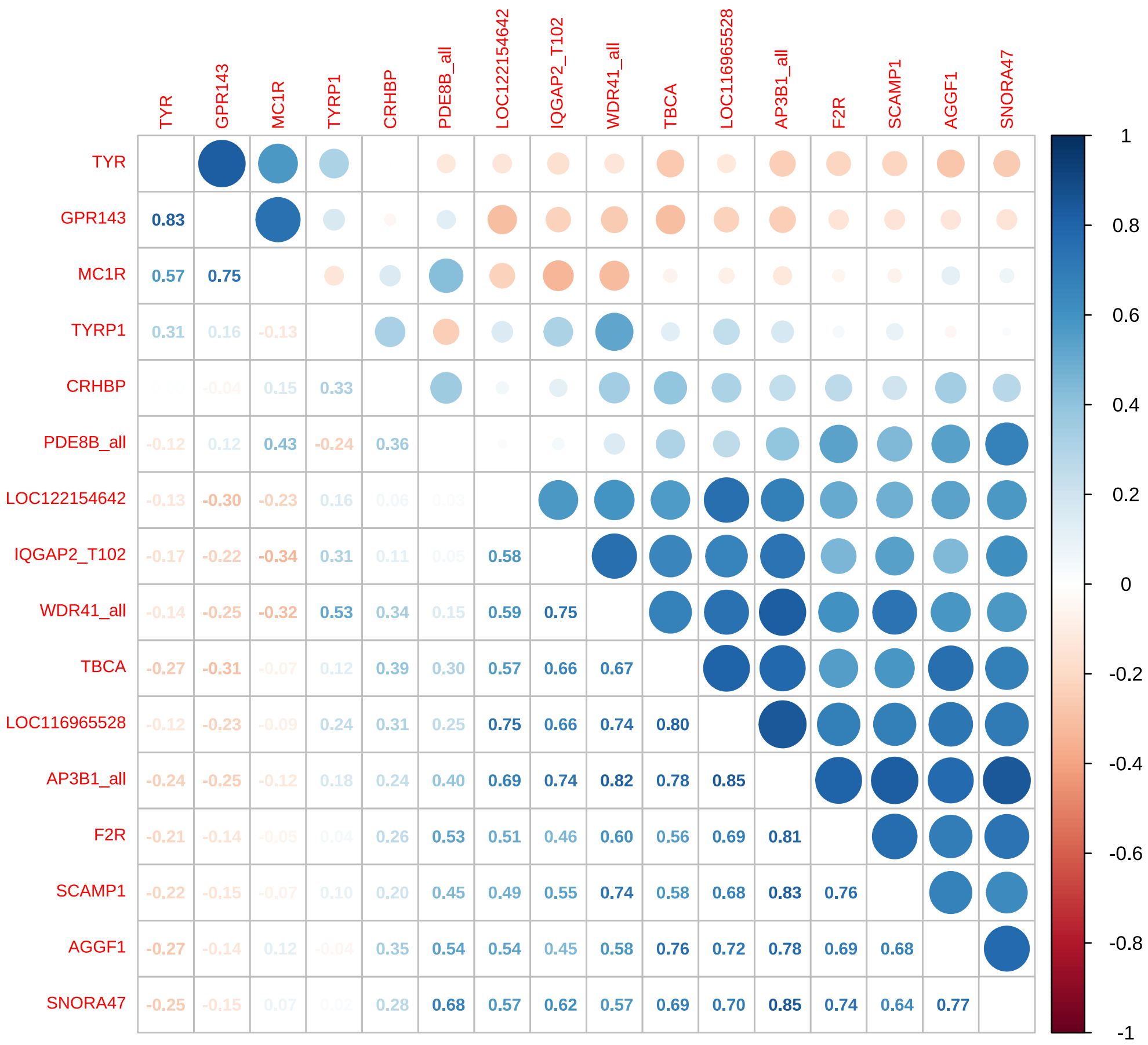

Figure S23 - Pearson's correlation between the relative gene expression for the primers-probe pairs represented either all transcripts of a gene or the most expressed transcript for IQGAP2.

Table S11 - Table comparing models explaining the expression without and with the genotype ((formula: "sqrt(Gene) ~ 1, random = ~1 | nestbox") and (formula: "sqrt(Gene) ~ as.factor(GenoZ), random = ~1 | nestbox")) for males (left) and females (right). Significant values after Bonferroni correction are highlighted in bold. BF stands for Bonferroni-corrected.

Gene	*p-value_Males*	*p-value_Males_BF*	*p-value_Females*	*p-value_Females_BF*
AGGF1	0.6059	1	0.1919	1
AP3B1_all	0.1874	1	0.2386	1
CRHBP	0.4339	1	0.5641	1
F2R	0.8578	1	0.1296	1
IQGAP2_T102	0.2778	1	0.251	1
LOC116965528	0.0882	1	0.4919	1
LOC122154642	0.0424	0.5088	0.2591	1
PDE8B_all	**0.0018**	**0.0216**	0.7921	1
SCAMP1	0.2601	1	0.6906	1
SNORA47	0.2833	1	0.2973	1
TBCA	0.2472	1	0.8725	1
WDR41_all	0.0783	0.9396	**0.0025**	**0.03**

Table S12 – Summary of the model linking the genotype and PDE8B expression in males.

	sqrt(PDE8B_all)		
Predictors	*Estimates*	*CI*	*p*
(Intercept)	1.06	0.92 – 1.20	**<0.001**
GenoZ [CT]	-0.34	-0.59 – -0.08	**0.032**
GenoZ [TT]	-0.28	-0.46 – -0.10	**0.009**
**Random Effects**			
σ^2	0.02		
$\tau_{00\ nestbox}$	0.00		
$N_{nestbox}$	15		
Observations	19		
Marginal R^2 / Conditional R^2	0.499 / NA		

Table S13 – Summary of the model linking the genotype and PDE8B expression in females.

	sqrt(PDE8B_all)		
Predictors	*Estimates*	*CI*	*p*
(Intercept)	0.56	0.39 – 0.73	**<0.001**
GenoZ [T]	-0.02	-0.23 – 0.18	0.813
**Random Effects**			
σ^2	0.02		
$\tau_{00\ nestbox}$	0.00		
$N_{nestbox}$	12		
Observations	13		
Marginal R^2 / Conditional R^2	0.006 / NA		

Table S14 – Summary of the model linking the genotype and WDR41 expression in males.

	sqrt(WDR41_all)		
Predictors	*Estimates*	*CI*	*p*
(Intercept)	0.93	0.79 – 1.07	**<0.001**
GenoZ [CT]	0.03	-0.23 – 0.29	0.753
GenoZ [TT]	-0.13	-0.31 – 0.05	0.174
**Random Effects**			
σ^2	0.02		
$\tau_{00\ nestbox}$	0.00		
$N_{nestbox}$	15		
Observations	19		
Marginal R^2 / Conditional R^2	0.245 / NA		

Table S15 – Summary of the model linking the genotype and WDR41 expression in females.

	sqrt(WDR41_all)		
Predictors	*Estimates*	*CI*	*p*
(Intercept)	0.71	0.66 – 0.76	**<0.001**
GenoZ [T]	-0.10	-0.16 – -0.04	**0.007**
**Random Effects**			
σ^2	0.00		
$\tau_{00\ nestbox}$	0.00		
$N_{nestbox}$	12		
Observations	13		
Marginal R^2 / Conditional R^2	0.525 / NA		

Table S16 – Summary of the model for linking PDE8B expression to spottiness.

	**SurfaceBlackSpotsCm**		
Predictors	*Estimates*	*CI*	*p*
(Intercept)	178.84	79.53 – 278.15	**0.002**
PDE8B all [sqrt]	-136.09	-256.42 – -15.77	**0.037**
Sex [2]	51.91	-0.87 – 104.70	0.064
**Random Effects**			
σ^2	1866.17		
$\tau_{00\ nestbox}$	2991.91		
ICC	0.62		
N nestbox	20		
Observations	32		
Marginal R^2 / Conditional R^2	0.342 / 0.747		

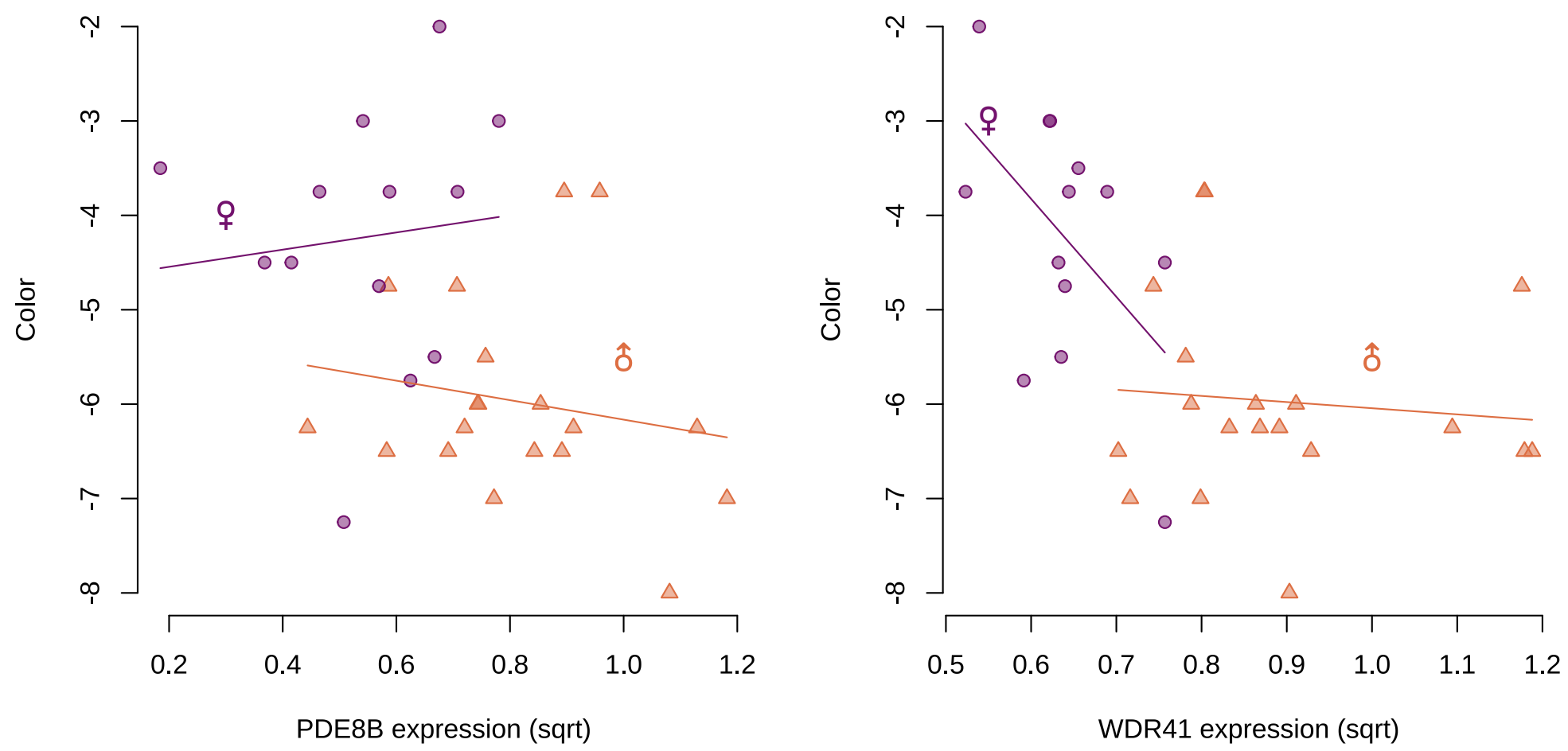

Figure S24 – Relation between PDE8B expression (left) and WDR41 (right) expression and the color of the individuals. Triangles (for males (♂), n = 28) and circles (for females (♀), n = 17) denote the 45 individuals with quantified gene expression. Lines represent the linear modelling of the relation within each sex.

Sexual dimorphism and sex specific genetic architecture

Genetic architecture of coloration

Table S17 – Summary of the model best model for the genetic architecture of the coloration in males.

Family: gaussian
Links: mu = identity; sigma = identity
Formula: MeanColor ~ 1 + as.factor(StageBin) + as.factor(GenoMC1R) + as.factor(GenoZcol) + as.factor(GenoCorin) + (1 | gr(ID, cov = grm))
Data: TabSummary[TabSummary$SexWGS == 1,] (Number of observations: 1480)
Draws: 4 chains, each with iter = 5000; warmup = 2000; thin = 1; total post-warmup draws = 12000

Group-Level Effects:
~ID (Number of levels: 1480)

	Estimate	Est.Error	l-95% CI	u-95% CI	Rhat	Bulk_ESS	Tail_ESS
sd(Intercept)	1.03	0.07	0.90	1.16	1.00	1074	2364

Population-Level Effects:

	Estimate	Est.Error	l-95% CI	u-95% CI	Rhat	Bulk_ESS	Tail_ESS
**Intercept**	**-7.34**	**0.11**	**-7.55**	**-7.13**	**1.00**	**6001**	**7850**
**as.factorStageBin2**	**0.67**	**0.05**	**0.57**	**0.77**	**1.00**	**9863**	**9330**
**as.factorGenoMC1R1**	**2.49**	**0.06**	**2.37**	**2.61**	**1.00**	**8210**	**8834**
**as.factorGenoMC1R2**	**3.40**	**0.15**	**3.10**	**3.69**	**1.00**	**8106**	**8927**
**as.factorGenoZcol1**	**0.34**	**0.07**	**0.21**	**0.47**	**1.00**	**6032**	**8199**
**as.factorGenoZcol2**	**0.93**	**0.09**	**0.76**	**1.11**	**1.00**	**5485**	**7036**
**as.factorGenoCorin1**	**0.28**	**0.07**	**0.13**	**0.42**	**1.00**	**6234**	**8319**
**as.factorGenoCorin2**	**0.37**	**0.09**	**0.19**	**0.54**	**1.00**	**6078**	**8086**

Family Specific Parameters:

	Estimate	Est.Error	l-95% CI	u-95% CI	Rhat	Bulk_ESS	Tail_ESS
sigma	0.73	0.03	0.67	0.80	1.00	1103	2037

Table S18 – Summary of the model best model for the genetic architecture of the coloration in females.

Family: gaussian
Links: mu = identity; sigma = identity
Formula: MeanColor ~ 1 + as.factor(StageBin) + as.factor(GenoMC1R) + as.factor(GenoZcol) + as.factor(GenoCorin) + as.factor(GenoMC1R * GenoZcol) + (1 | gr(ID, cov = grm))
Data: TabSummary[TabSummary$SexWGS == 2,] (Number of observations: 1603)
Draws: 4 chains, each with iter = 5000; warmup = 2000; thin = 1; total post-warmup draws = 12000

Group-Level Effects: ~ID (Number of levels: 1603)

	Estimate	Est.Error	l-95% CI	u-95% CI	Rhat	Bulk_ESS	Tail_ESS
sd(Intercept)	0.97	0.07	0.83	1.10	1.00	918	1832

Population-Level Effects:

	Estimate	Est.Error	l-95% CI	u-95% CI	Rhat	Bulk_ESS	Tail_ESS
Intercept	-5.84	0.08	-5.99	-5.69	1.00	7596	8701
as.factorStageBin2	0.71	0.05	0.61	0.81	1.00	11827	9407
as.factorGenoMC1R1	2.19	0.08	2.02	2.36	1.00	7040	8272
as.factorGenoMC1R2	2.93	0.23	2.47	3.39	1.00	7879	8678
as.factorGenoZcol1	0.88	0.07	0.75	1.01	1.00	8314	9244
as.factorGenoCorin1	0.18	0.07	0.04	0.31	1.00	7578	9057
as.factorGenoCorin2	0.32	0.08	0.16	0.49	1.00	6757	8399
as.factorGenoMC1RMUGenoZcol1	-0.31	0.11	-0.53	-0.10	1.00	7335	8280
as.factorGenoMC1RMUGenoZcol2	-0.79	0.30	-1.37	-0.20	1.00	8228	8810

Family Specific Parameters:

	Estimate	Est.Error	l-95% CI	u-95% CI	Rhat	Bulk_ESS	Tail_ESS
sigma	0.74	0.03	0.67	0.81	1.00	922	1892

Genetic architecture of spottiness

Table S19 – Summary of the model best model for the genetic architecture of the spottiness in males

Family: hurdle_gamma
Links: mu = log; shape = identity; hu = logit
Formula:
SurfaceBlackSpotsCm ~ 1 + as.factor(StageBin) + as.factor(GenoZspot) + as.factor(GenoMC1R) + (1 | gr(ID, cov = grm))
hu ~ 1 + as.factor(StageBin) + as.factor(GenoZspot) + as.factor(GenoMC1R) + (1 | gr(ID, cov = grm))
Data: TabSummary[TabSummary$SexWGS == 1,] (Number of observations: 1194)
Draws: 4 chains, each with iter = 5000; warmup = 2000; thin = 1; total post-warmup draws = 12000

Group-Level Effects: ~ID (Number of levels: 1194)

	Estimate	Est.Error	l-95% CI	u-95% CI	Rhat	Bulk_ESS	Tail_ESS
sd(Intercept)	0.61	0.07	0.47	0.76	1.00	1733	3754
sd(hu_Intercept)	3.04	0.99	1.48	5.35	1.01	994	1910

Population-Level Effects:

	Estimate	Est.Error	l-95% CI	u-95% CI	Rhat	Bulk_ESS	Tail_ESS
Intercept	-1.08	0.10	-1.29	-0.89	1.00	4028	6458
**hu_Intercept**	**-1.45**	**0.54**	**-2.66**	**-0.56**	**1.00**	**3075**	**4056**
**as.factorStageBin2**	**0.31**	**0.06**	**0.20**	**0.42**	**1.00**	**12172**	**8532**
**as.factorGenoZspot1**	**0.85**	**0.08**	**0.69**	**1.01**	**1.00**	**7154**	**8050**
**as.factorGenoZspot2**	**1.82**	**0.10**	**1.63**	**2.02**	**1.00**	**5138**	**7606**
**as.factorGenoMC1R1**	**0.57**	**0.07**	**0.44**	**0.70**	**1.00**	**7127**	**8797**
**as.factorGenoMC1R2**	**0.38**	**0.15**	**0.09**	**0.68**	**1.00**	**9962**	**9324**
hu_as.factorStageBin2	0.74	0.43	-0.03	1.69	1.00	5143	4451
**hu_as.factorGenoZspot1**	**-3.66**	**0.78**	**-5.54**	**-2.46**	**1.01**	**1390**	**2181**
**hu_as.factorGenoZspot2**	**-6.79**	**1.57**	**-10.46**	**-4.36**	**1.00**	**2258**	**3492**
**hu_as.factorGenoMC1R1**	**-3.47**	**0.82**	**-5.34**	**-2.13**	**1.01**	**2375**	**3090**
**hu_as.factorGenoMC1R2**	**-29.98**	**26.28**	**-101.31**	**-3.48**	**1.00**	**5123**	**4569**

Family Specific Parameters:

	Estimate	Est.Error	l-95% CI	u-95% CI	Rhat	Bulk_ESS	Tail_ESS
shape	1.67	0.10	1.49	1.88	1.00	2369	5242

Table S20 – Summary of the model best model for the genetic architecture of spottiness in females

Family: hurdle_gamma
Links: mu = log; shape = identity; hu = logit
Formula:
SurfaceBlackSpotsCm ~ 1 + as.factor(StageBin) + GenoZspot + GenoMC1R + (1 | gr(ID, cov = grm))
hu ~ 1 + as.factor(StageBin) + GenoZspot + GenoMC1R + (1 | gr(ID, cov = grm))
Data: TabSummary[TabSummary$SexWGS == 2,] (Number of observations: 1286)
Draws: 4 chains, each with iter = 10000; warmup = 5000; thin = 1; total post-warmup draws = 20000

Group-Level Effects: ~ID (Number of levels: 1286)

	Estimate	Est.Error	l-95% CI	u-95% CI	Rhat	Bulk_ESS	Tail_ESS
sd(Intercept)	0.47	0.05	0.37	0.56	1.00	2806	6109
sd(hu_Intercept)	1.22	0.95	0.05	3.54	1.00	2778	2353

Population-Level Effects:

	Estimate	Est.Error	l-95% CI	u-95% CI	Rhat	Bulk_ESS	Tail_ESS
Intercept	0.18	0.04	0.10	0.27	1.00	9816	14007
**hu_Intercept**	**-4.98**	**0.97**	**-7.35**	**-3.59**	**1.00**	**4250**	**2535**
**as.factorStageBin2**	**0.31**	**0.04**	**0.23**	**0.38**	**1.00**	**25605**	**14287**
**GenoZspot**	**0.85**	**0.04**	**0.77**	**0.94**	**1.00**	**14683**	**15165**
GenoMC1R	-0.08	0.04	-0.15	0.00	1.00	20307	14499
hu_as.factorStageBin2	1.01	0.71	-0.26	2.51	1.00	15558	8570
**hu_GenoZspot**	**-2.59**	**1.03**	**-4.99**	**-0.92**	**1.00**	**17052**	**9081**
**hu_GenoMC1R**	**-0.89**	**0.79**	**-2.66**	**0.46**	**1.00**	**21779**	**11906**

Family Specific Parameters:

	Estimate	Est.Error	l-95% CI	u-95% CI	Rhat	Bulk_ESS	Tail_ESS
shape	2.86	0.17	2.55	3.21	1.00	3629	7564

Characterizing evolutionary process driving allelic and phenotypic changes

Observed allelic frequency changes

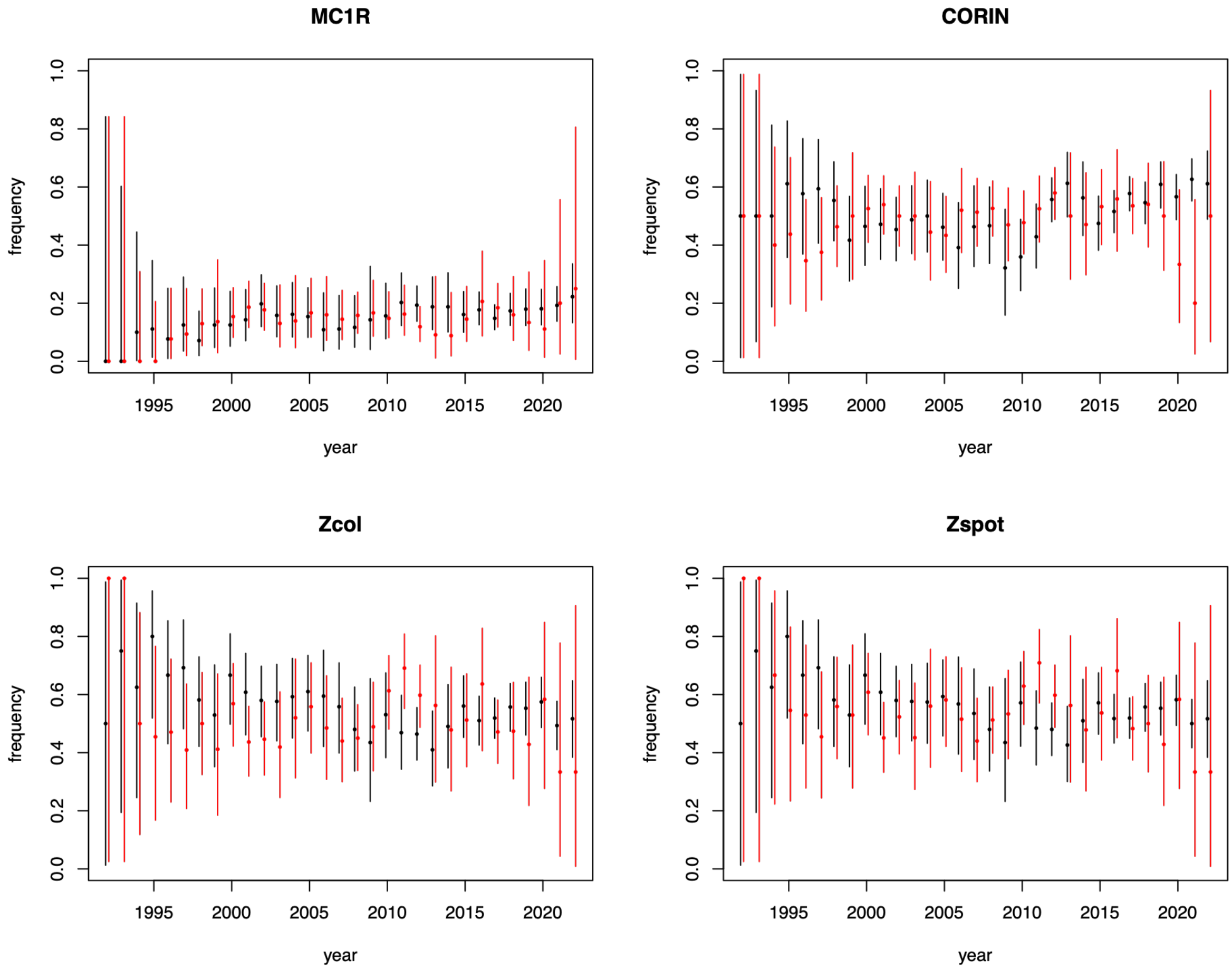

Figure S25 – Evolution of the allelic frequencies in residents (black) and migrants (red) across the years for the major effect loci for the coloration (namely MC1R, CORIN and Zcol) and Spottiness (namely Zspot). Diamond depicts the estimate while vertical bars represent the confidence interval.

Table S21 – Summary of the model of allelic frequency change as a function of year for MC1R

```
Call:
lm(formula = FullFreqMC1R[Years > 1995] ~ Years[Years > 1995])

Residuals:
     Min        1Q    Median        3Q       Max
-0.041945 -0.012034 -0.001173  0.010482  0.051237

Coefficients:
                     Estimate Std. Error t value Pr(>|t|)
(Intercept)        -5.4395163  1.0171266  -5.348 1.52e-05 ***
Years[Years > 1995]  0.0027848  0.0005063   5.500 1.03e-05 ***
---
Signif. codes:  0 ‘***’ 0.001 ‘**’ 0.01 ‘*’ 0.05 ‘.’ 0.1 ‘ ’ 1

Residual standard error: 0.02049 on 25 degrees of freedom
Multiple R-squared:  0.5475,	Adjusted R-squared:  0.5295
F-statistic: 30.25 on 1 and 25 DF,  p-value: 1.027e-05
```

Table S22 – Summary of the model of allelic frequency change as a function of year for Z

```
Call:
lm(formula = FullFreqZ[Years > 1995] ~ Years[Years > 1995])

Residuals:
     Min        1Q    Median        3Q       Max
-0.079542 -0.027526 -0.005824  0.026571  0.070911

Coefficients:
                     Estimate Std. Error t value Pr(>|t|)
(Intercept)          4.9215914  1.9888035   2.475   0.0205 *
Years[Years > 1995] -0.0021796  0.0009899  -2.202   0.0371 *
---
Signif. codes:  0 ‘***’ 0.001 ‘**’ 0.01 ‘*’ 0.05 ‘.’ 0.1 ‘ ’ 1

Residual standard error: 0.04007 on 25 degrees of freedom
Multiple R-squared:  0.1624,	Adjusted R-squared:  0.1289
F-statistic: 4.848 on 1 and 25 DF,  p-value: 0.03713
```

Evolutionary forces driving the frequency changes

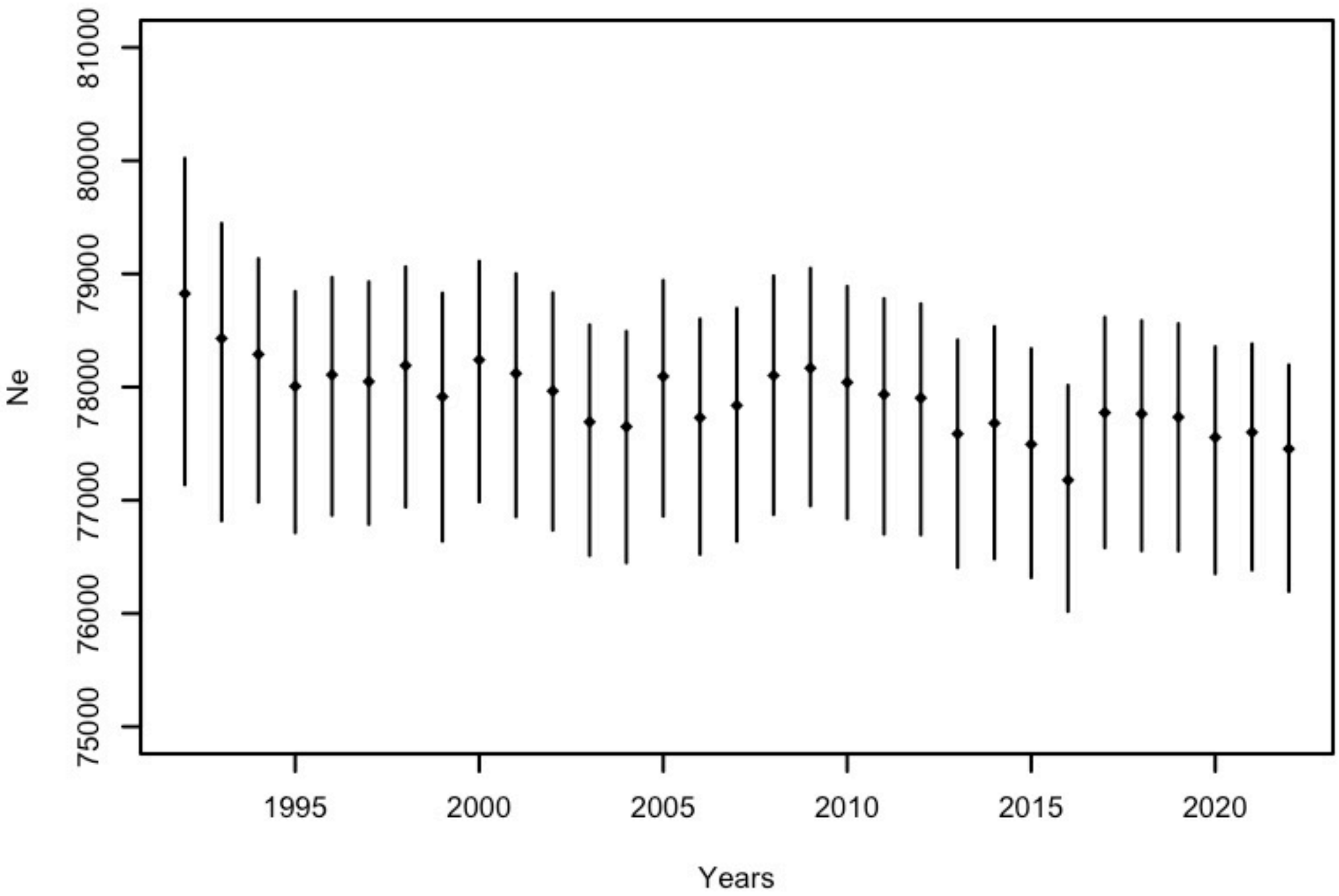

Figure S26 – Effective population size (*Ne*) of the Swiss population across the study period. For each year, *Ne* was estimated using the adults alive that year. dot depicts the estimate while vertical bars represent the confidence interval across 100 bootstraps.

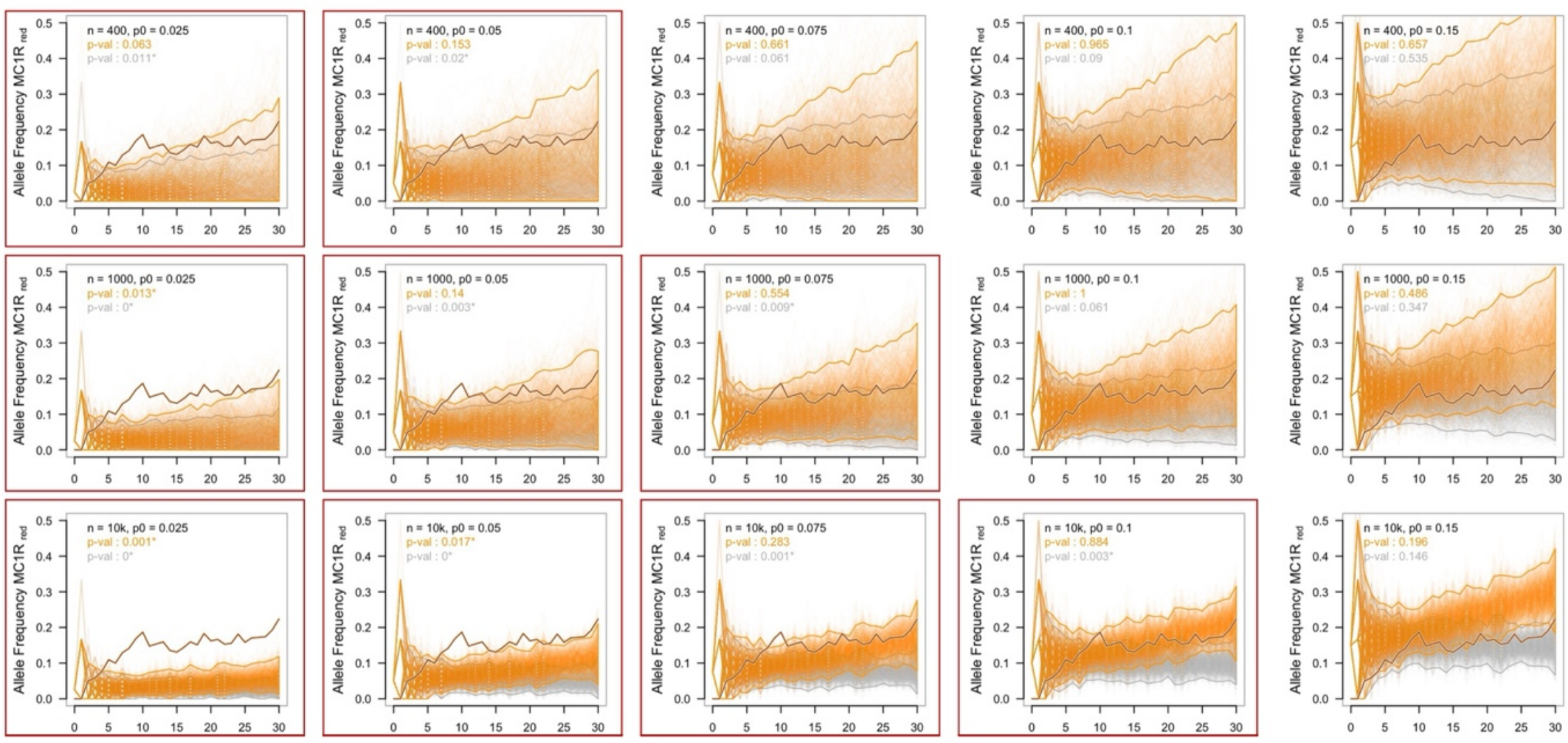

Figure S27 – Results from forward simulations of allele frequency change at the MC1R locus under different combinations of population size (400, 1,000, and 10,000 individuals; rows) and initial allele frequency (0.025, 0.05, 0.075, 0.10, and 0.15; columns). Each line represents a single simulation replicate (grey for neutral genetic drift, orange for simulations including selection), and the solid line indicates the 95% confidence interval across replicates. The red line shows the observed allele

frequency trajectory over the study period for the MC1R red allele. P-values shown in the top left indicate the probability of observing the empirical data under each scenario (orange for the selection scenario, grey for the neutral scenario). Red boxes highlight combinations of population size and selection coefficient for which neutrality is rejected.

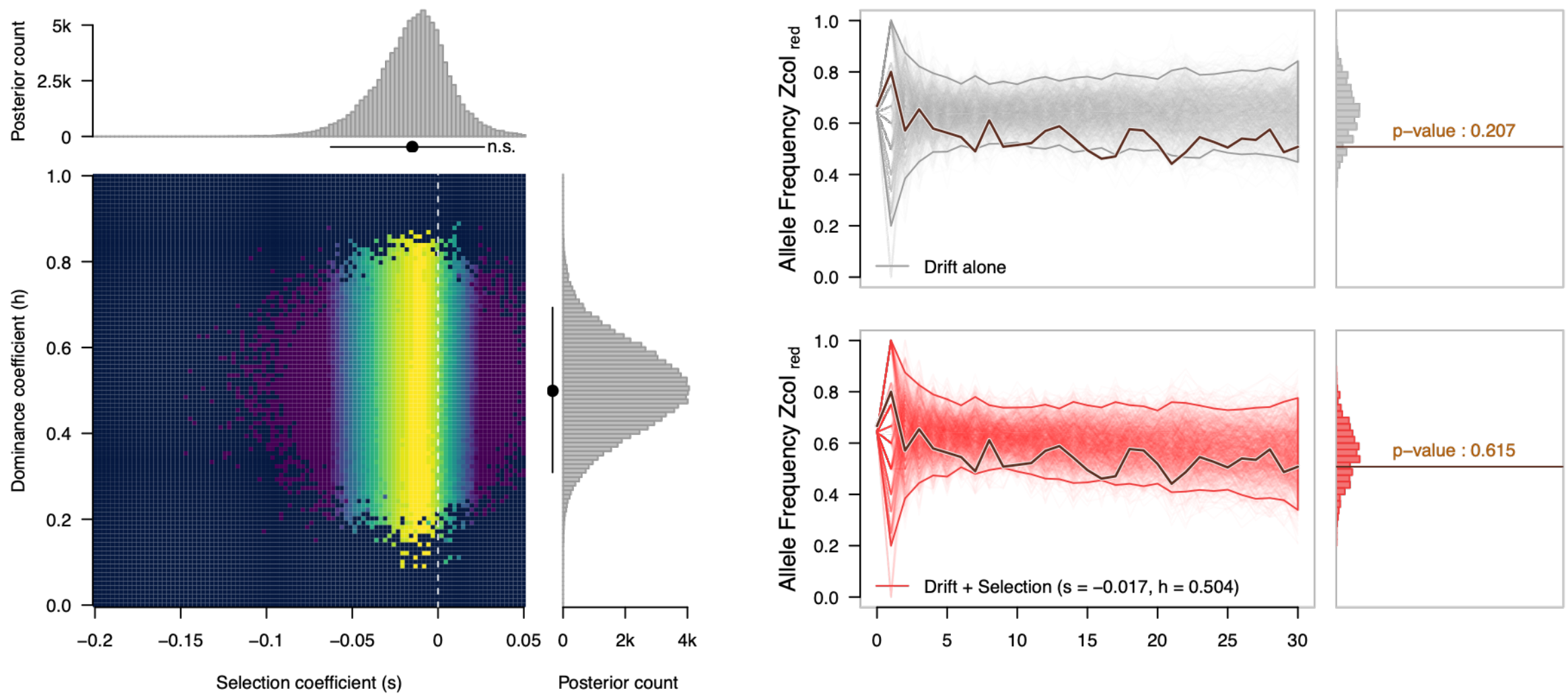

Figure S28 – Likelihood-based inference of selection parameters and simulation-based validation for the Z color locus. (left) Heatmaps show the log-likelihood surface across a grid of selection coefficients (s) and dominance coefficients (h), with warmer colors indicating higher likelihood of the observed allele frequency trajectory for a given parameter combination. Overlaid bar plots represent the posterior distributions of s and h, summarizing parameter uncertainty; points below the histograms mark posterior means, and bars indicate 95% confidence intervals. Significance was assessed by whether the confidence interval overlaps zero. (right) Results from forward simulations of allele frequency change under two evolutionary scenarios: genetic drift alone (top) and drift combined with selection (bottom). Each line represents a single simulation replicate (grey for neutral drift, orange or brown for simulations including selection), and the solid line shows the 95% confidence interval across replicates. Dark brown line indicate the observed allele frequency trajectories over the study period for the Z $_{red}$ allele. Histograms on the right show the distribution of final allele frequencies across replicates, and p-values indicate the probability of observing the empirical data under each scenario.

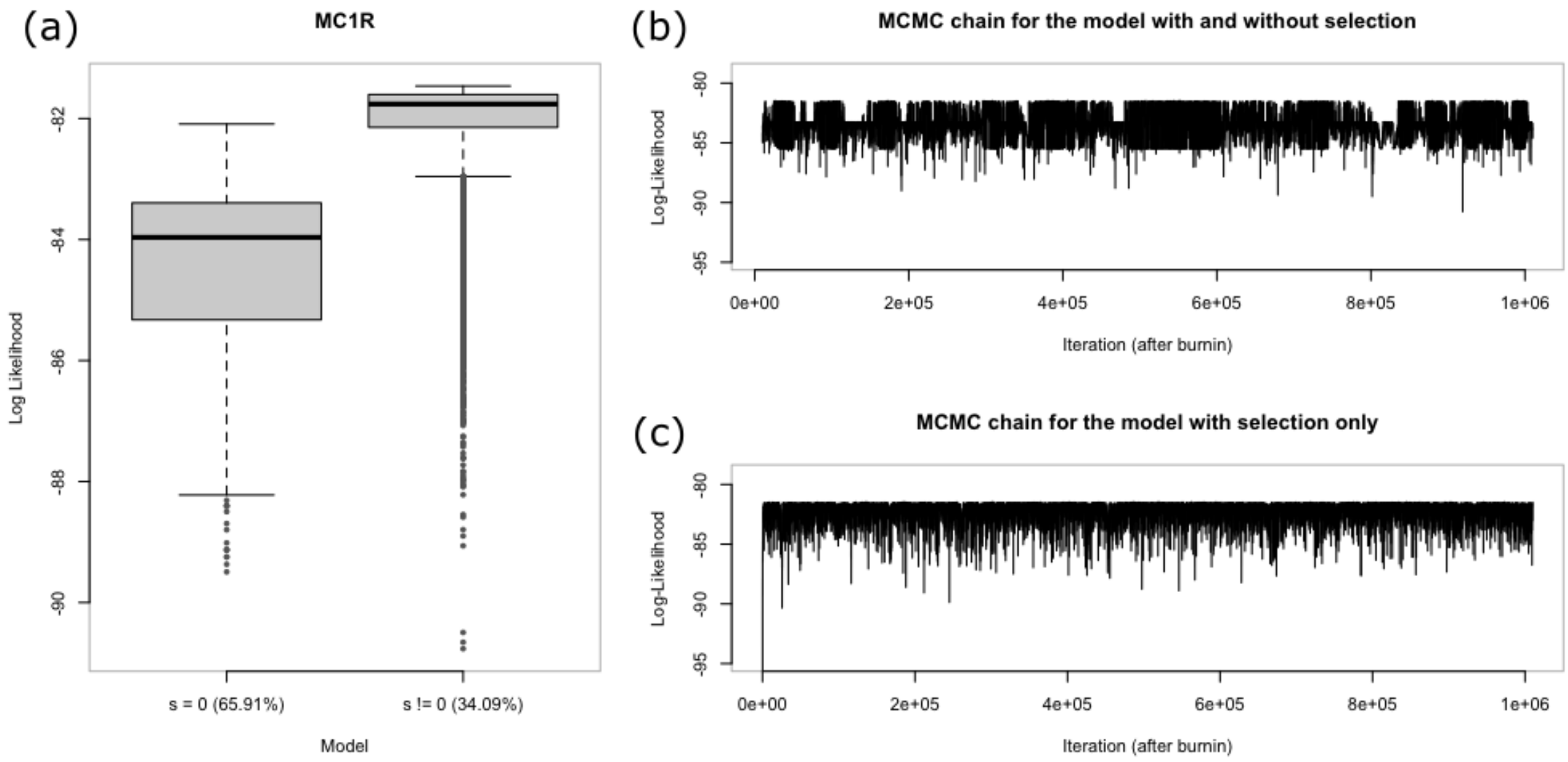

Figure S29 – Results of the approxWF framework for the $MC1R_{red}$ allele. (a) Boxplot showing the distribution of log-likelihood values for two models at the MC1R locus: selection (s!=0) and no selection (s = 0). Values in brackets indicate the proportion of time the MCMC chain spent in each model. (b) Log-likelihood trace along the MCMC chain for the model allowing jumps between selection and no-selection states at the MC1R locus, inferred using approxWF. (c) Log-likelihood trace along the MCMC chain for the model restricted to the selection state at the MC1R locus, inferred using approxWF.

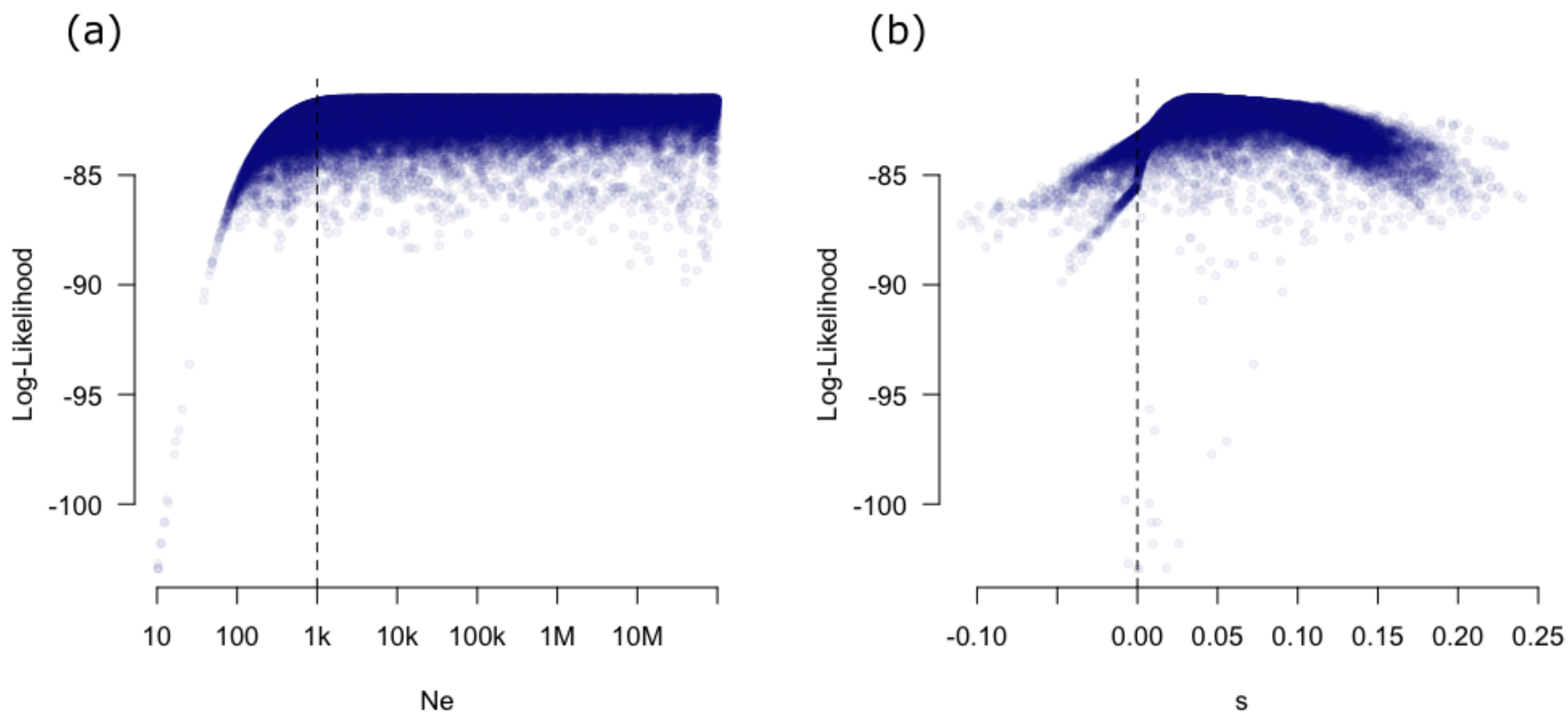

Figure S30 – Log-likelihood values across approxWF iterations as a function of (a) *Ne* and (b) *s* values for the MC1R locus.

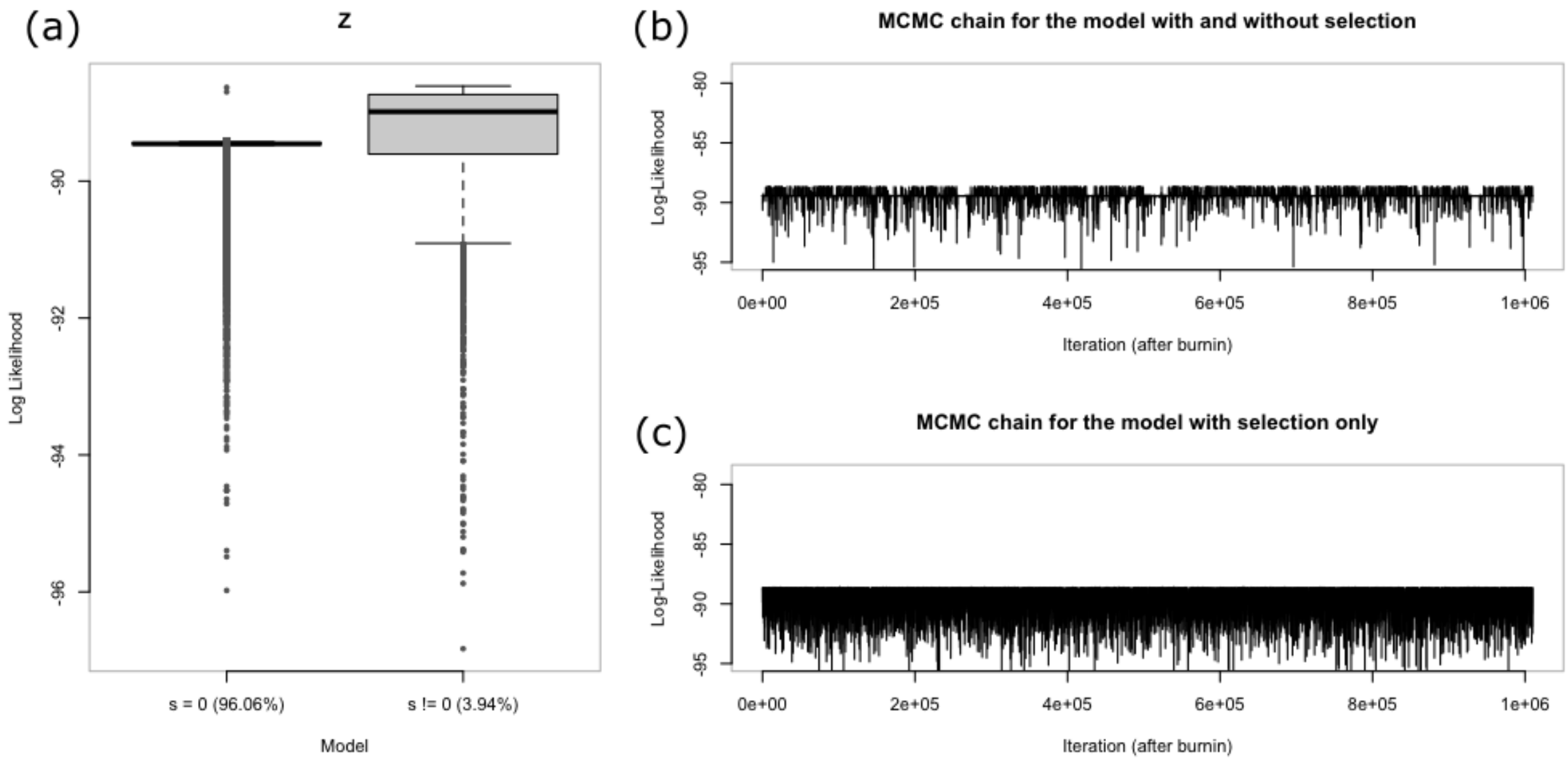

Figure S31 – Results of the approxWF framework for $Z_{spotted}$ allele. (a) Boxplot showing the distribution of log-likelihood values for two models at the Z locus: selection (s!=0) and no selection (s = 0). Values in brackets indicate the proportion of time the MCMC chain spent in each model. (b) Log-likelihood trace along the MCMC chain for the model allowing jumps between selection and no-selection states at the Z locus, inferred using approxWF. (c) Log-likelihood trace along the MCMC chain for the model restricted to the selection state at the MC1R locus, inferred using approxWF.

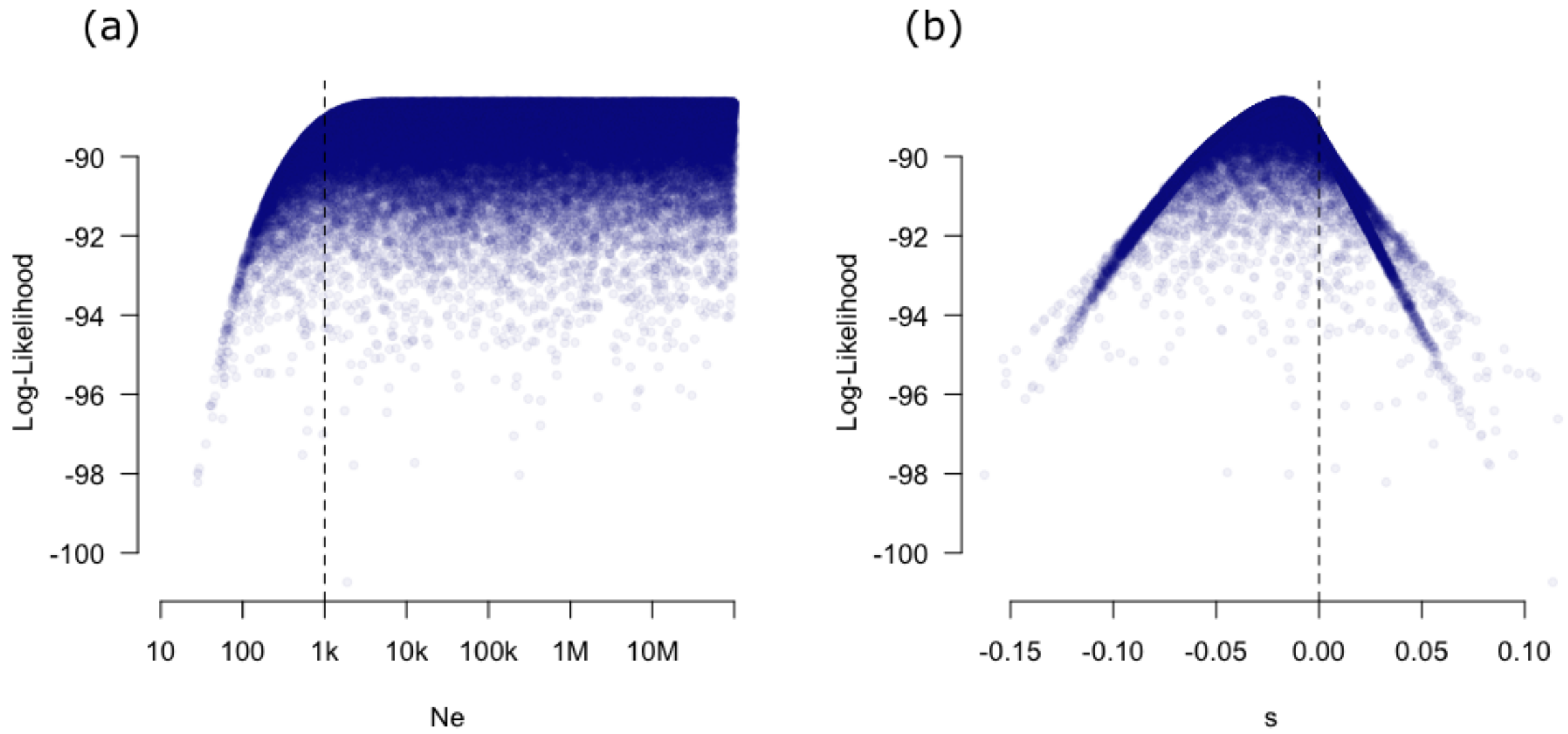

Figure S32 – Log-likelihood values across approxWF iterations as a function of (a) *Ne* and (b) *s* values for the Z spot locus.

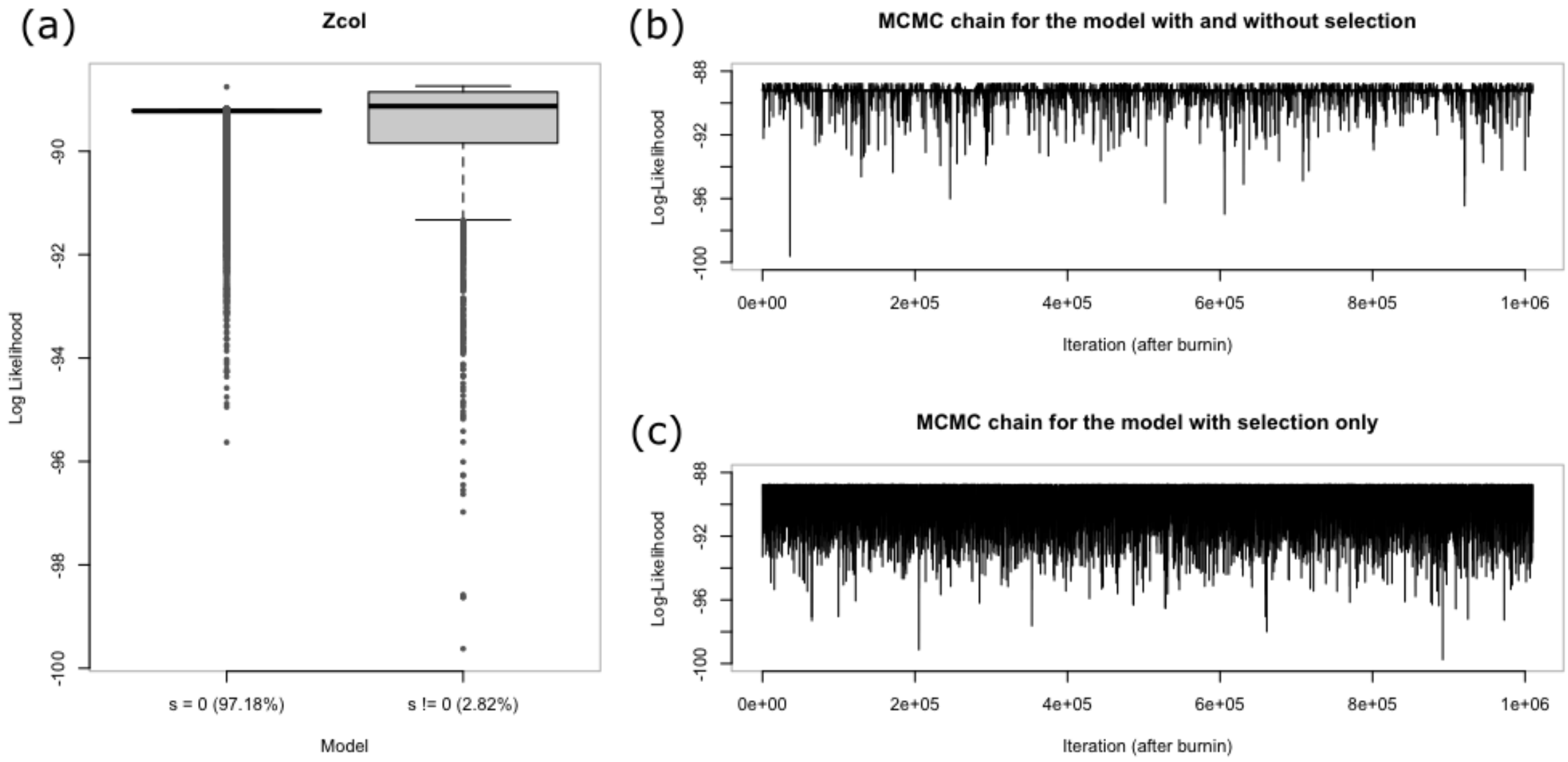

Figure S33 – Results of the approxWF framework for the Z_{red} allele. (a) Boxplot showing the distribution of log-likelihood values for two models at the MC1R locus: selection (s!=0) and no selection (s = 0). Values in brackets indicate the proportion of time the MCMC chain spent in each model. (b) Log-likelihood trace along the MCMC chain for the model allowing jumps between selection and no-selection states at the Z locus, inferred using approxWF. (c) Log-likelihood trace along the MCMC chain for the model restricted to the selection state at the Z locus, inferred using approxWF.

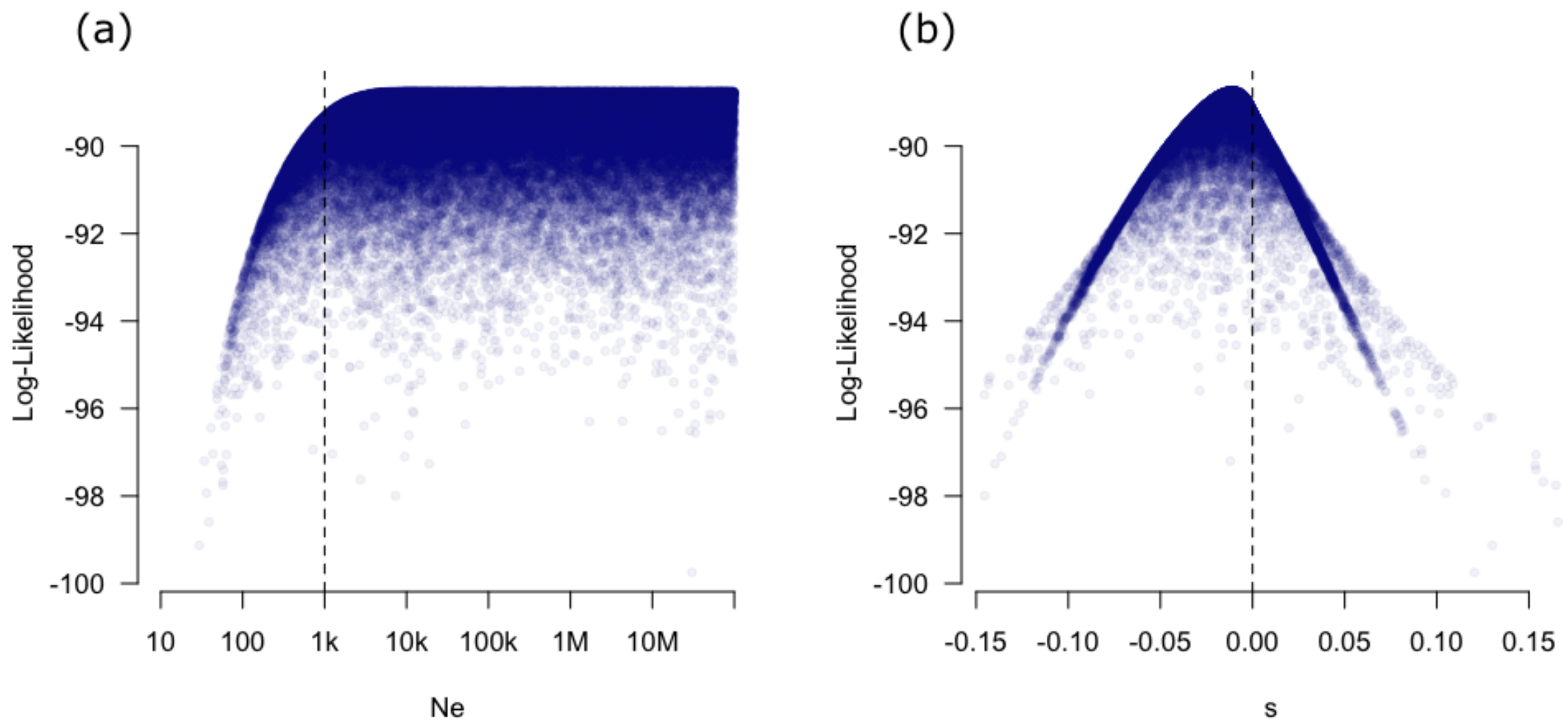

Figure S34 – Log-likelihood values across approxWF iterations as a function of (a) *Ne* and (b) *s* values for the Z color locus.

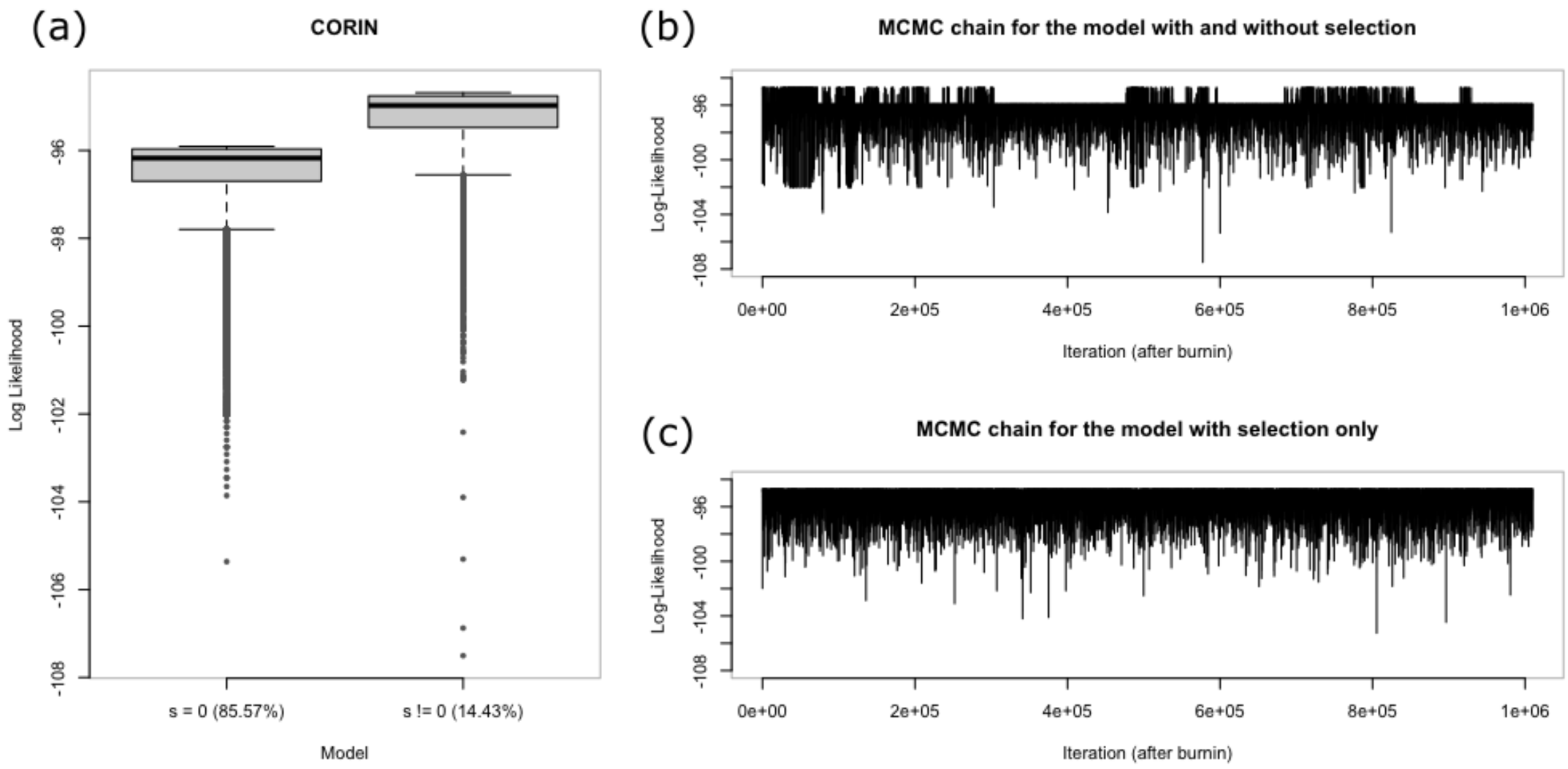

Figure S35 – Results of the approxWF framework for $CORIN_{red}$ allele. (a) Boxplot showing the distribution of log-likelihood values for two models at the MC1R locus: selection (s!=0) and no selection (s = 0). Values in brackets indicate the proportion of time the MCMC chain spent in each model. (b) Log-likelihood trace along the MCMC chain for the model allowing jumps between selection and no-selection states at the CORIN locus, inferred using approxWF. (c) Log-likelihood trace along the MCMC chain for the model restricted to the selection state at the CORIN locus, inferred using approxWF.

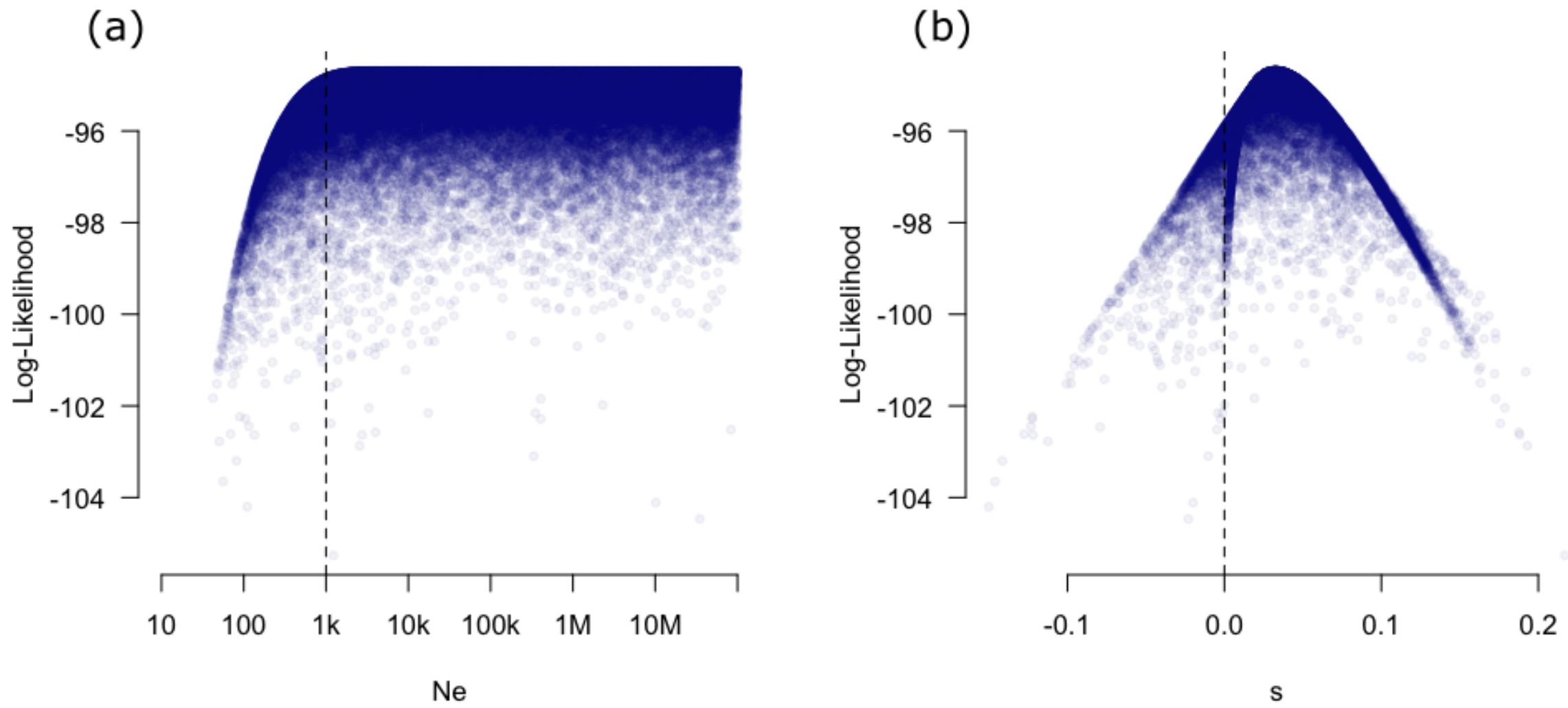

Figure S36 – Log-likelihood values across approxWF iterations as a function of (a) *Ne* and (b) *s* values for the CORIN locus.

Supplementary Note 1: Acknowledgments for Sample Collection

We are deeply grateful to all the individuals who generously contributed their time and effort to collecting samples over the past 30 years. This work would not have been possible without their dedication and support. The following list includes the names of those who assisted; however, it may not be entirely complete. We sincerely apologize for any unintentional omissions and extend our heartfelt thanks to everyone who involved during field work and sample collection.

alphabetically sorted list:
Albanese Margherita, Allemann Roxane, Amrein Martin, Antoniazza Silvain, Ançay Laurie, Arévalo Olympe, Audusseau Hélène, Bastardot Marc, Battesti Marine, Becciu Paolo, Bincteux Manon, Bissegger Silvan, Braaker Sonja, Bragoni Maeva, Burri Patricia, Béziers Paul, Büchi Uli, Bühler Roman, Calvani Morgane, Campon Carla, Chausson Alexandre, Chevalier Florian, Chèvre Maxime, Clément Laura, Cordes Jana, Dahuron Marine, De Gianni Lara, Deillon Clara, Demaizière Zoé, Desjardins Chloé, Dessonet Justin, Dijoux Lorine, Dolivo Vassilissa, Dreiss Amélie, Ducouret Pauline, Ducret Valérie, Dugas Mélanie, Déturche Marie, Edmé Anaïs, Ehinger Jonathan, Fioravanti Céline, Fischer Simon, Flint Raphaelle, Florent Gaëlle, Forestier Auréline, Fougère Maëlle, Frey Caroline, Freyburger Maxime, Gaillard Maryline, Gaime Florence, San-Jose Garcia Luis, Gaudy Ladina, Gelle Nathan, Gellé Nathan, Gerber Aline, Gervasio Giulia, Graf Valentin, Grangier-Bijou Anna, Gremion Jérémy, Grognuz Vincent, Guilbaut Emy, Gunter Jodok, Gémard Charlène, Heintz Anne-Caroline, Henry Isabelle, Hernandez Lucile, Hertach Matthias, Huulas Lisa, Häfliger Manuela, Ioset Noemie, Jamet Jessica, Jeanneret Yann, Judes Clarisse, Juillerat Juliette, Junker Kilian, Karsegard Charlotte, Kilchoer Noémie, Koller Andreia, Konrad Maureen, Kotur Mia, König Pascal, Küttel Stefan, Laurent Thomas, Laurent Aurélie, Le Grand Luc, Lejeune Ilou, Letuppe Thomas, Luisier Célestin, Machado Ana Paula, Marcadet Alexis, Massa Carolina, Mathis Felicia, Milliet Estelle, Morgenthaler Annick, Moulin Nicolas, Nicole Sidonie, Noll Mélissa, Nuber Maria, Oberli Frédéric, Oliveira-Xavier Aymeric, Orelli Fabien, Pedraita Serena, Pellegrini Ester, Perroud Giulia, Pilati Marco, Pini Jonathan, Pittet Jeanne, Plancherel Céline, Poncet Lisa, Questiaux Anastasia, Quetel Tiphaine, Ramseier Deborah, Rau Rebecca, Reichhart Birgit, Repnik Leona, Rieser Andreas, Robadey Aurélien, Roidt Myriam, Roland Alexandre, Romano Andrea, Rossier Virginie, Roulet Adrien, Ruppli Charlène, Rusca Arianna, Rüttimann Silvan, Sager Ramon, Sahli Christophe, Salin Kathleen, Schalcher Kim, Schmid Baptiste, Scriba Madeleine, Sironi Nicolas, Sonnay Caroline, Spori Carole, Stier Kim, Ston Daniel, Streit Jonas, Séchaud Robin, Théro Héloïse, Unger Marc, Van den Brink Valentijn, Vasseur Julien, Vermunt Aurélie, Villain Nicolas, Wassef Jérôme, Wolf Franziska, Zurkinden Damien, Zurkinden Steve.
