# Supplementary material for: Genomic bases of short-term evolution in wild barn owls revealed by long-term monitoring and population-scale sequencing
Source: Nat Commun. 2026 Oct 12;17:11545. doi: 10.1038/s41467-026-78632-4 (PMC13748587; doi:10.1038/s41467-026-78632-4)
Supplement: Supplementary file 2 [file 41467_2026_78632_MOESM2_ESM.pdf]

<u>Description Of Additional Supplementary Files</u>

Supplementary Data 1 – Choice of the best model explaining the evolution of the color (see Supplementary tables). Significance was assessed by likelihood-ratio tests comparing nested linear mixed-effects models. All tests were two-sided.

Supplementary Data 2 – Choice of the best model explaining the evolution of the spottiness (see Supplementary tables). Significance was assessed by likelihood-ratio tests comparing nested linear mixed-effects models. All tests were two-sided.

Supplementary Data 3 – Selection of the best model explaining color variation, excluding the first five years of the study period to avoid potential bias due to low sample size (from 1998 - see Supplementary Tables). Significance was assessed by likelihood-ratio tests comparing nested linear mixed-effects models. All tests were two-sided.

Supplementary Data 4 – Choice of the best model explaining the evolution of the spottiness, excluding the first five years of the study period to avoid potential bias due to low sample size (from 1998 - see Supplementary tables). Significance was assessed by likelihood-ratio tests comparing nested linear mixed-effects models. All tests were two-sided.

Supplementary Data 5 – List of SNPs significant in the Genome Wide Association (GWA) study between the genotypes and the **plumage coloration of the entire ventral part.**

Supplementary Data 6 – List of SNPs significant in the Genome Wide Association (GWA) study between the genotypes and the **plumage coloration of the breast.**

Supplementary Data 7 – List of SNPs significant in the Genome Wide Association (GWA) study between the genotypes and the **plumage coloration of the belly.** Statistical significance was assessed using a Bonferroni-corrected significance threshold to account for multiple testing.

Supplementary Data 8 – List of SNPs significant in the Genome Wide Association (GWA) study between the genotypes and the **ventral spottiness.** Statistical significance was assessed using a Bonferroni-corrected significance threshold to account for multiple testing.

Supplementary Data 9 – List of SNPs significant in the Genome Wide Association (GWA) study between the genotypes and the mean **diameter of the spots.** Statistical significance was assessed using a Bonferroni-corrected significance threshold to account for multiple testing.

Supplementary Data 10 – List of SNPs significant in the Genome Wide Association (GWA) study between the genotypes and the **number of spots.** Statistical significance was assessed using a Bonferroni-corrected significance threshold to account for multiple testing.

Supplementary Data 11 – List of genes harboring at least one SNP significantly associated with the traits in one or more of the GWAS analyses presented in this study. Genes were retained if a significant SNP was located within the gene or within a 5 kb flanking region. Each column corresponds to a specific GWAS analysis and indicates whether the gene contains at least one significantly associated SNP.

Supplementary Data 12 - Sequences and efficiency of the probe and primer pairs used to quantify the gene present on the Z chromosome.

Supplementary Data 13 – Selection of the best model explaining the color in males.

Supplementary Data 14 – Selection of the best model explaining the color in females.

Supplementary Data 15 – Selection of the best model explaining the spottiness in males.

Supplementary Data 16 – Selection of the best model explaining the spottiness in females.
