# Supplementary material for: Genomic bases of short-term evolution in wild barn owls revealed by long-term monitoring and population-scale sequencing
Source: Nat Commun. 2026 Oct 12;17:11545. doi: 10.1038/s41467-026-78632-4 (PMC13748587; doi:10.1038/s41467-026-78632-4)
Supplement: Supplementary file 3 [file 41467_2026_78632_MOESM4_ESM.pdf]

nature portfolio

Corresponding author(s): Tristan Cumer

Last updated by author(s): Sep 3, 2026

Reporting Summary

Nature Portfolio wishes to improve the reproducibility of the work that we publish. This form provides structure for consistency and transparency in reporting. For further information on Nature Portfolio policies, see our Editorial Policies and the Editorial Policy Checklist.

Statistics

For all statistical analyses, confirm that the following items are present in the figure legend, table legend, main text, or Methods section.

n/a	Confirmed	
☐	☒	The exact sample size (*n*) for each experimental group/condition, given as a discrete number and unit of measurement
☐	☒	A statement on whether measurements were taken from distinct samples or whether the same sample was measured repeatedly
☐	☒	The statistical test(s) used AND whether they are one- or two-sided<br>*Only common tests should be described solely by name; describe more complex techniques in the Methods section.*
☐	☒	A description of all covariates tested
☐	☒	A description of any assumptions or corrections, such as tests of normality and adjustment for multiple comparisons
☐	☒	A full description of the statistical parameters including central tendency (e.g. means) or other basic estimates (e.g. regression coefficient) AND variation (e.g. standard deviation) or associated estimates of uncertainty (e.g. confidence intervals)
☐	☒	For null hypothesis testing, the test statistic (e.g. *F*, *t*, *r*) with confidence intervals, effect sizes, degrees of freedom and *P* value noted<br>*Give P values as exact values whenever suitable.*
☐	☒	For Bayesian analysis, information on the choice of priors and Markov chain Monte Carlo settings
☐	☒	For hierarchical and complex designs, identification of the appropriate level for tests and full reporting of outcomes
☐	☒	Estimates of effect sizes (e.g. Cohen's *d*, Pearson's *r*), indicating how they were calculated

Our web collection on statistics for biologists contains articles on many of the points above.

Software and code

Policy information about availability of computer code

Data collection

NA

Data analysis

Trimmomatic v0.39 and v.0.36
BWA-MEM v0.7.17 and v.0.7.15
GATK v4.2.6
WhatsHap v1.4
SHAPEIT v4.1.2
Qualimap v2.2.1
BCFTools v1.19
GLIMPSE v1.1.1

approxWF (comit 85793eb)

R (v4.2.2)
lme4 package (v1.1-37)
hierfstat package (v0.5-11)
brms package (version 2.34 and v2.0.22)
gaston package (v1.62)
RefSeeker package (v1.0.4)
nlme package (v3.1-168)

Code used to prepare the genomic data can be found at: https://github.com/cumtr/Cumer_et_al_EvolutionColor_WGS and archived on Zenodo under DOI:
10.5281/zenodo.22278101 (https://zenodo.org/records/22278101).

Code used for downstream analyses can be found at: https://github.com/cumtr/Cumer_et_al_EvolutionColor_Analyses and archived on Zenodo under DOI: 10.5281/zenodo.22278594 (https://zenodo.org/records/22278594).

For manuscripts utilizing custom algorithms or software that are central to the research but not yet described in published literature, software must be made available to editors and reviewers. We strongly encourage code deposition in a community repository (e.g. GitHub). See the Nature Portfolio guidelines for submitting code & software for further information.

Data

Policy information about availability of data

All manuscripts must include a data availability statement. This statement should provide the following information, where applicable:

- Accession codes, unique identifiers, or web links for publicly available datasets
- A description of any restrictions on data availability
- For clinical datasets or third party data, please ensure that the statement adheres to our policy

The data underlying this article are available in the GenBank Sequence Read Archive Database at https://www.ncbi.nlm.nih.gov/sra. High coverage sequences used for the reference panel can be accessed within BioProject PRJNA700797, PRJNA727915, PRJNA727977, PRJNA774943, PRJNA925445 and PRJNA1172395. Low coverage sequences can be accessed within BioProject PRJNA1391678.

Research involving human participants, their data, or biological material

Policy information about studies with human participants or human data. See also policy information about sex, gender (identity/presentation), and sexual orientation and race, ethnicity and racism.

Reporting on sex and gender	NA
Reporting on race, ethnicity, or other socially relevant groupings	NA
Population characteristics	NA
Recruitment	NA
Ethics oversight	NA

Note that full information on the approval of the study protocol must also be provided in the manuscript.

Field-specific reporting

Please select the one below that is the best fit for your research. If you are not sure, read the appropriate sections before making your selection.

☐ Life sciences ☐ Behavioural & social sciences ☒ Ecological, evolutionary & environmental sciences

For a reference copy of the document with all sections, see nature.com/documents/nr-reporting-summary-flat.pdf

Ecological, evolutionary & environmental sciences study design

All studies must disclose on these points even when the disclosure is negative.

Study description	We analyzed long-term phenotypic data to characterize temporal changes in two fitness-related traits: ventral coloration and spottiness. Using whole-genome sequencing, we identified the genetic basis of these traits. Gene expression analyses highlighted candidate genes associated with trait variation. Finally, we integrated temporal genomic data to test whether genetic drift alone explains observed allele frequency changes at key loci or whether selection has acted on these traits.
Research sample	This study is based on long-term monitoring of a wild barn owl (Tyto alba) population in western Switzerland, conducted continuously since 1986 and spanning more than 30 years. These owls primarily breed in nest boxes installed on farms. During each breeding season, nest boxes were inspected at four-week intervals to assess occupancy. When an active nest was detected, females were captured during incubation, while males were typically caught later during nestling provisioning. All nestlings and any previously unmarked adults were fitted with uniquely numbered metal rings for individual identification.<br>At each capture event, two melanin-based plumage traits—coloration and spottiness—were assessed on both the breast and belly of each individual. Blood samples were collected from the brachial vein of each bird and initially stored in liquid nitrogen before being transferred to -80°C freezers for long-term preservation.
Sampling strategy	For phenotypic data, we used all available information collected throughout the study period. For whole-genome sequencing,

Sampling strategy	individuals were selected to maximize the availability of phenotypic and fitness-related data among those with stored blood samples. For gene expression analyses, feathers were sampled from nestlings at the developmental stage when feathers begin to exhibit black spots on their apical tips and the characteristic white-to-reddish pheomelanin coloration.
Data collection	Phenotypic measurements were recorded in the field by multiple observers over the course of the long-term study. Blood samples were collected from the brachial vein of each bird and initially stored in liquid nitrogen before being transferred to -80°C freezers for long-term preservation.
Timing and spatial scale	The population of barn owls (Tyto alba) in western Switzerland (46°49′ N, 06°56′ E) has been continuously monitored since 1986.
Data exclusions	No data were excluded from the analysis.
Reproducibility	All phenotypic data used in this study have been deposited on Zenodo (DOI XXXX). All genomic data have been deposited in the Sequence Read Archive (SRA).
Randomization	For sequencing, samples were randomly distributed across plates, with two negative controls included per plate. No plate effects or contamination were detected.
Blinding	Blinding was not feasible in this study because it involved the capture and monitoring of wild animals rather than a controlled experimental design. Phenotypic measurements and sample collection were performed during routine field monitoring, where blinding is impractical.

Did the study involve field work? ☒ Yes ☐ No

Field work, collection and transport

Field conditions	Field study extending over 30 years in Western Switzerland
Location	western Switzerland (46°49′ N, 06°56′ E)
Access & import/export	NA
Disturbance	All animal handling procedures were conducted in accordance with international guidelines for animal welfare and the Swiss Animal Welfare Act. The study protocols were reviewed and approved by the cantonal veterinary authorities of Vaud and Fribourg, Switzerland. Animal experimentation permits were issued by the Veterinary Offices of the Cantons of Vaud and Fribourg under permit numbers 1736 (2004), 1508 (2007), 2177 (2010), 2109 (2012), 2844 (2015), 2934 (2016), 3462 (2020), 3571 (2020 and renewed in 2023), 3213 (2021), and 3884 (2025). Authorizations for work involving protected wildlife, including owl ringing, were issued by the Swiss Federal Office for the Environment (FOEN).

Reporting for specific materials, systems and methods

We require information from authors about some types of materials, experimental systems and methods used in many studies. Here, indicate whether each material, system or method listed is relevant to your study. If you are not sure if a list item applies to your research, read the appropriate section before selecting a response.

Materials & experimental systems

n/a	Involved in the study	
☒	☐	Antibodies
☒	☐	Eukaryotic cell lines
☒	☐	Palaeontology and archaeology
☐	☒	Animals and other organisms
☒	☐	Clinical data
☒	☐	Dual use research of concern
☒	☐	Plants

Methods

n/a	Involved in the study	
☒	☐	ChIP-seq
☒	☐	Flow cytometry
☒	☐	MRI-based neuroimaging

Animals and other research organisms

Policy information about studies involving animals; ARRIVE guidelines recommended for reporting animal research, and Sex and Gender in Research

Laboratory animals	NA
Wild animals	This study focuses on the European barn owl (Tyto alba). The population in western Switzerland (46°49′ N, 06°56′ E) has been continuously monitored since 1986. Nest boxes, typically located around barns in the study area, were inspected at four-week

	intervals to assess occupancy. When an active nest was detected, females were captured during incubation, while males were typically caught later during nestling provisioning. All nestlings and any previously unmarked adults were fitted with uniquely numbered metal rings for individual identification.
Reporting on sex	Sex of the individuals was infered using molecular markers.
Field-collected samples	Blood samples were collected from the brachial vein of each bird and initially stored in liquid nitrogen before being transferred to -80°C freezers for long-term preservation.
Ethics oversight	All animal handling procedures were conducted in accordance with international guidelines for animal welfare and the Swiss Animal Welfare Act. The study protocols were reviewed and approved by the cantonal veterinary authorities of Vaud and Fribourg, Switzerland. Animal experimentation permits were issued by the Veterinary Offices of the Cantons of Vaud and Fribourg under permit numbers 956 (1996), 1146 (1998), 1736 (2004), 1508 (2007), 2177 (2010), 2109 (2012), 2844 (2015), 2934 (2016), 3462 (2020), 3571 (2020 and renewed in 2023), 3213 (2021), and 3884 (2025). Authorizations for work involving protected wildlife, including owl ringing, were issued by the Swiss Federal Office for the Environment (FOEN).

Note that full information on the approval of the study protocol must also be provided in the manuscript.

Plants

Seed stocks	*Report on the source of all seed stocks or other plant material used. If applicable, state the seed stock centre and catalogue number. If plant specimens were collected from the field, describe the collection location, date and sampling procedures.*
Novel plant genotypes	*Describe the methods by which all novel plant genotypes were produced. This includes those generated by transgenic approaches, gene editing, chemical/radiation-based mutagenesis and hybridization. For transgenic lines, describe the transformation method, the number of independent lines analyzed and the generation upon which experiments were performed. For gene-edited lines, describe the editor used, the endogenous sequence targeted for editing, the targeting guide RNA sequence (if applicable) and how the editor was applied.*
Authentication	*Describe any authentication procedures for each seed stock used or novel genotype generated. Describe any experiments used to assess the effect of a mutation and, where applicable, how potential secondary effects (e.g. second site T-DNA insertions, mosiacism, off-target gene editing) were examined.*
