# Supplementary material for: Genomic bases of short-term evolution in wild barn owls revealed by long-term monitoring and population-scale sequencing
Source: Nat Commun. 2026 Oct 12;17:11545. doi: 10.1038/s41467-026-78632-4 (PMC13748587; doi:10.1038/s41467-026-78632-4)
Supplement: Supplementary file 4 [file 41467_2026_78632_MOESM5_ESM.pdf]

nature portfolio

Peer Review File

Genomic bases of short-term evolution in wild barn owls revealed by long-term monitoring and population-scale sequencing

Corresponding Author: Dr Tristan Cumer

This file contains all reviewer reports in order by version, followed by all author rebuttals in order by version.

Version 0:

Reviewer comments:

Reviewer #1

(Remarks to the Author)
This is a compelling study that leverages three decades of phenotypic monitoring and population-scale whole-genome sequencing of over 3,000 barn owls to investigate the genomic basis of rapid phenotypic evolution in two melanin-based plumage traits (coloration and spottiness). The authors demonstrate that both traits have undergone directional phenotypic change over the 30-year study period and identify an oligogenic architecture involving few loci of major effect: MC1R on chromosome 11, a region on the Z chromosome, and CORIN on chromosome 4 (the latter detected only in the breast-specific GWAS). Gene expression analyses in developing feathers provide suggestive, though preliminary, evidence linking Z-linked genotypes to spottiness via PDE8B expression in males, though no significant expression-phenotype link was found for coloration. Forward simulations and probabilistic modeling indicate that selection, rather than drift alone, can explain the allele frequency shift at MC1R, while changes at the Z-linked locus remain consistent with neutral expectations.

I reviewed this paper collaboratively with my postdoc, and we really enjoyed reading and discussing the study. Overall, this study addresses foundational questions in evolutionary biology, asking how phenotypic change maps onto genomic change in real time, and does so with a rare, long-term dataset that few research groups have the capacity to generate. The combination of a long-term monitoring program, population-wide sequencing of thousands of individuals, phenotypic data spanning three decades, and integration of GWAS, gene expression, and evolutionary simulations represents a substantial contribution to the field. The manuscript is generally well-written, and the analyses are thorough and robust. The methods are detailed and generally well-described. The genomic pipeline, from reference panel construction and phasing through low-coverage imputation and validation, is rigorous. We appreciated that the authors validated their imputation approach using independent samples and documented their quality control steps thoroughly.

We have several broad comments and suggestions for the authors to consider below.

COMMENTS
Results & Discussion

Gene expression: The gene expression analyses provide a step toward connecting the GWAS-identified Z-linked region to a molecular mechanism, but the results remain preliminary, which the authors acknowledge themselves. Of the 12 candidate genes tested, only two showed significant genotype-dependent expression (PDE8B in males, WDR41 in females), and only one of these (PDE8B) was significantly associated with spottiness in males, but not coloration.
However, the genotype distribution for MC1R was highly unbalanced (38 homozygous white, 6 heterozygous, 1 homozygous red), which precluded the analysis of MC1R expression effects entirely. This limits interpretation given that MC1R is the locus with the strongest evidence for selection. It would strengthen the manuscript if the authors elevated their discussion of these limitations, particularly regarding the inability to account for MC1R genotype variation in the expression models and the single significant expression-phenotype link. This may help in framing the gene expression section more proportionally to the strength of the evidence it provides. As it stands, the narrative gives this component somewhat more interpretive weight than the data currently support.

Simulation parameters: The authors estimate Ne at ~226,000, which they attribute to high historical effective population size

and contemporary connectivity across European barn owl populations. However, they use the census population size of N = 400 for the forward drift simulations, which is described as a conservative estimate consistent with the lowest census sizes during the study period. We appreciate the conservative framing, but given the large difference between these two values, it would be helpful if the authors could more explicitly discuss why census N is the more appropriate parameter for the drift simulations and how sensitive the results are to this choice. Using a smaller population size produces wider neutral envelopes, which would make drift easier to reject. As a reader, it would be beneficial to understand whether the selection inference at MC1R holds under alternative population size assumptions closer to Ne.

Analytical roadmap: We found it somewhat difficult to follow the reasoning for which loci were chosen for the simulation analyses. For instance, why was MC1R prioritized for the selection-versus-drift analysis over the Z-linked locus, given that the Z-linked region is associated with both traits and shows the larger effect on spottiness? We recognize that both loci are ultimately analyzed, but the narrative structure could benefit from a clearer explanation of the decision-making process guiding which loci were modeled and why.

Along these lines, the manuscript presents a series of increasingly focused analyses, but the subset of loci carried forward into each stage differs without always making the rationale explicit. For instance, CORIN is included in the coloration genetic architecture models but not in the allele frequency or simulation analyses, and the Z-linked region is represented by different top SNPs across analyses (all in high LD, but the positions differ). While each individual analysis appears well-executed, we think the manuscript would benefit from a brief roadmap clarifying which loci are carried into each analytical stage and why. As a reader, we felt this would help track the decision-making logic without having to reconstruct it across sections.

Phenotypic scoring methodology: The authors employ an eight-point visual color chip scale for scoring plumage coloration. While this approach has been used in prior studies, there is now a substantial body of work employing spectrophotometry and digital imaging for quantifying melanin-based plumage variation in birds. Have the authors compared or validated this visual scoring method against more objective quantitative approaches? If validation studies exist for this scoring system, citing them would help readers assess the precision and repeatability of the phenotypic measurements underpinning the study.

Environmental contributions to the phenotypic trend: The phenotypic trend models in supplement use year of birth as a predictor without environmental covariates, and year is inherently confounded with any directional environmental change over the study period. The allele frequency analyses provide convincing independent evidence that the phenotypic trends have a genetic basis, and melanin-based plumage traits are generally considered to have low environmental plasticity. Nonetheless, a brief acknowledgment that the phenotypic models alone cannot fully disentangle genetic from environmental contributions to the temporal trend would add useful nuance, particularly for readers less familiar with the heritability of melanin-based traits.

Reviewer #2

(Remarks to the Author)
I co-reviewed this manuscript with one of the reviewers who provided the listed reports. This is part of the Nature Communications initiative to facilitate training in peer review and to provide appropriate recognition for Early Career Researchers who co-review manuscripts.

Reviewer #3

(Remarks to the Author)
In the article “Genomic bases of short-term evolution in the wild revealed by long-term monitoring and population-scale sequencing,” the authors leverage a 30-year dataset of barn owls to connect genotype with phenotype and study their changes over time. This is a truly enormous dataset that provides a unique opportunity to study plumage color in birds! I have a few major concerns that I detail below that will help clarify a number of points in the manuscript, though I suspect these will not change the primary results of the paper. Overall, this was an exciting read and I believe there will be a lot of interest in the results from the ornithology, evolutionary biology, and color evolution readership!

MAJOR COMMENTS:

(1) One of my primary concerns is around the framing of the paper and how this matches with the actual results that are included. The abstract and introduction set up the focus of the paper to include fitness and climate change (e.g., L42-44), but these felt very underdeveloped in the included results. Much of the argument the authors have built rests on previous connections between fitness and plumage color/spottiness (L88-90), but there aren’t enough details included here to understand how/why that relationship might exist. What is the directionality of the relationship? (Just based on the results included here, I assume darker/spottier is better…?) What is the suspected mechanism underlying plumage’s role? And perhaps most importantly, why do you expect this is changing over time? Based on the abstract I was expecting there would be an analysis connecting observed phenotypic changes to some kind of environmental variable and a direct assessment of individual survival/reproduction based on phenotype to show how these have changed over time. It seems like information on offspring survival exists, is it possible to integrate that directly into this manuscript? Further exploring this would help support the latter simulation results on the role of selection versus drift.

(2) I had a few concerns about the dataset that I feel need to be addressed. The largest concern is about the difference in

sample size of the first year of the data (1992). As shown in Figure S4 and S5, the sample size of each of the sexes is quite small in this year, which seems to heavily influence the distribution of observed phenotypes (such that the means are pretty different from the subsequent years). I think this isn't necessarily a huge problem, but I do wonder if this is influencing things like the simulation analyses included in Figure 5. The MC1R simulations have an observed allele frequency that starts nearly at 0! How influential is this initial year in the identified changes over time? The allele frequencies shown in Figure S23 seem pretty different for those early years (for both MC1R and Z), so I think it will be really important to confirm that the lower sample size is not creating an outsized effect on the overall observed patterns across different analyses.

(3) Finally, I believe more details on the classification of color are needed here. There is very little detail included on the determination of the pheomelanin coloration. What does it actually mean to be -8 versus -1 on your color chips? What color chips are you actually using? Additionally, there are a number of questions about the developmental patterns of the plumage color and spotting that should be addressed. How does plumage color in nestlings compare to adults? Is it actually reasonable to lump both nestlings and adults into the same dataset for the analyses you include in this manuscript? Is including related family groups within the dataset biasing your results? Does the plumage color change as adults age? Especially as "stage [fledgling]" pops up as a significant predictor in many of the models included in the supplement and Figure S7 suggests there are significant differences between adults and juveniles, it seems important to address these issues directly.

MINOR COMMENTS:

It could be helpful to include an in-text table that lists the identified genes and which trait they are associated with.

L63-70. This introduction here feels a bit out of date… many studies have now studied phenotypes in wild populations, particularly for bird coloration.

L72-83. Additionally, I felt that many of these citations were not well matched with the introduction here. They felt both overly broad and then also oddly specific in different places. There is a TON of literature on the genomics of bird coloration that was overlooked and I think would greatly strengthen the intro.

L96. I was a bit confused about why explaining only 80% of the variation in this traits was used as a motivator for the study here. This feels like a HUGE amount of variation when compared to most studies of wild organisms, so this didn't feel particularly exciting as a motivator.

L127-128. Given this population is open to immigrants and the trend holds with them as well, what is the expected selection pressure? It seems like it would need to be something that is working on a continental or global scale?

L194. Typo in "our work results refine…"

L195-197. What trait was this gene related to in rosy finches? Does it seem to be doing the same thing as in barn owls?

L231. Was this also in birds?

L249-264. The discussion of CORIN was quite disjunct here. It was only mentioned once briefly before this and then is pretty underdeveloped here. Particularly as this gene has been found related to coloration in other birds, it seems like it might be helpful to go into a bit more detail. (e.g., Wood et al. 2023 Current Biology, Bourgeois et al. 2016 J Heredity)

L249. Missing parenthesis

L263-264. This statement feels a bit overblown…

L323-324. But you're now talking about range-wide selection, no?

L325. It could be helpful to indicate where the mutation rate estimate is coming from. (I know you include it in the methods, but I would just add it here too.)

L357. Missing a word here: "further supporting the [role] of selection"

L360. Missing a word here: "between the selection and drift models [was] modest"

L409. Equation shows a square here—likely an error post-submitting

L413. Missing words here: "The life stage at which [an] individual was phenotype[d]"

L582. Missing a word here: "lead to artificially [elevated?] accuracy due to"

L593. Hanging sentence here. What was excluded?

L712. Typo: "Quiagen" should be "Qiagen"

L919. Typo: “Montel Carlo” should be “Monte Carlo”

L933. What does “a substantial portion of MCMC iterations” mean here?

FIGURE COMMENTS:

Figure 1B: This scatterplot was very difficult to interpret! Especially in comparison to the same trends shown in Figure S2. It would be helpful to change the opacity of the points and/or leverage point size to clearly display the overlap of points, add in a trendline to make the relationship clearer, and do away with the different point shapes (which I am inferring relate to sex?). The size of the figure makes it difficult to even differentiate shapes here, and I suggest leaving the separate plots to Figure S2.

Figure 2: As there is a lot of whitespace here, it would be helpful to include some labelling within this figure. Perhaps you could add gene names to A and B, and indicate which GWAS is for color vs spots? It would also be helpful to add some more labelling details to C, D, and E. It could be worth highlighting which genes are the ones of interest in the middle gray bars, so it’s clear where they are located in association to the GWAS peak. Finally, the LD plots are slightly offset from the axis of the GWAS peak, and it would be helpful to make sure those match up one-to-one.

Figure 3: The font in this figure is particularly small, and it would similarly benefit from additional labelling within the figure. Especially as the different panels move from phenotype to genotype and back again as you go from top to bottom.

Figure 5: It would be helpful to add “MC1R” and “Z” as labels to the top and bottom panels here. Additionally, the message shown in A/D will be clearer if the s=0 line is marked on the heatmap, as that will very visually show that the MC1R analysis results in s>0. Finally, are the dark brown lines shown in B/C/E/F the observed allele frequency (as compared to the simulation shown in lightly lines)? If so, it would be helpful to include that info in the legend, and potentially change the color / thickness of the line so that it’s really distinguishable from the lines indicating the outside edges of the simulations.

Version 1:

Reviewer comments:

Reviewer #1

(Remarks to the Author)
This is our second review of the paper entitled “Genomic bases of short-term evolution in the wild revealed by long-term monitoring and population-scale sequencing”. I again reviewed this version with my postdoc.

We appreciate the authors' thorough and thoughtful responses to the reviewer comments. The revisions have substantially strengthened the manuscript, and we believe the authors have satisfactorily addressed all of our previous concerns. We have no additional substantive comments and feel this manuscript will be of broad interest.

Reviewer #2

(Remarks to the Author)
I co-reviewed this manuscript with one of the reviewers who provided the listed reports. This is part of the Nature Communications initiative to facilitate training in peer review and to provide appropriate recognition for Early Career Researchers who co-review manuscripts.

Reviewer #3

(Remarks to the Author)
I appreciate the care taken when responding to reviewer comments and believe the manuscript is greatly improved. It's a very thorough study and will be an exciting addition to the literature!

Reviewer #4

(Remarks to the Author)
I co-reviewed this manuscript with one of the reviewers who provided the listed reports. This is part of the Nature Communications initiative to facilitate training in peer review and to provide appropriate recognition for Early Career Researchers who co-review manuscripts.

Open Access This Peer Review File is licensed under a Creative Commons Attribution 4.0 International License, which permits use, sharing, adaptation, distribution and reproduction in any medium or format, as long as you give appropriate credit to the original author(s) and the source, provide a link to the Creative Commons license, and indicate if changes were made.
In cases where reviewers are anonymous, credit should be given to 'Anonymous Referee' and the source.
The images or other third party material in this Peer Review File are included in the article's Creative Commons license, unless indicated otherwise in a credit line to the material. If material is not included in the article's Creative Commons license and your intended use is not permitted by statutory regulation or exceeds the permitted use, you will need to obtain permission directly from the copyright holder.
To view a copy of this license, visit https://creativecommons.org/licenses/by/4.0/

Reviewer #1 (Remarks to the Author):

This is a compelling study that leverages three decades of phenotypic monitoring and population-scale whole-genome sequencing of over 3,000 barn owls to investigate the genomic basis of rapid phenotypic evolution in two melanin-based plumage traits (coloration and spottiness). The authors demonstrate that both traits have undergone directional phenotypic change over the 30-year study period and identify an oligogenic architecture involving few loci of major effect: MC1R on chromosome 11, a region on the Z chromosome, and CORIN on chromosome 4 (the latter detected only in the breast-specific GWAS). Gene expression analyses in developing feathers provide suggestive, though preliminary, evidence linking Z-linked genotypes to spottiness via PDE8B expression in males, though no significant expression-phenotype link was found for coloration. Forward simulations and probabilistic modeling indicate that selection, rather than drift alone, can explain the allele frequency shift at MC1R, while changes at the Z-linked locus remain consistent with neutral expectations.

I reviewed this paper collaboratively with my postdoc, and we really enjoyed reading and discussing the study. Overall, this study addresses foundational questions in evolutionary biology, asking how phenotypic change maps onto genomic change in real time, and does so with a rare, long-term dataset that few research groups have the capacity to generate. The combination of a long-term monitoring program, population-wide sequencing of thousands of individuals, phenotypic data spanning three decades, and integration of GWAS, gene expression, and evolutionary simulations represents a substantial contribution to the field. The manuscript is generally well-written, and the analyses are thorough and robust. The methods are detailed and generally well-described. The genomic pipeline, from reference panel construction and phasing through low-coverage imputation and validation, is rigorous. We appreciated that the authors validated their imputation approach using independent samples and documented their quality control steps thoroughly.

We have several broad comments and suggestions for the authors to consider below.

[R] We thank you for your positive feedback on our manuscripts and constructive comments. Please find below a detailed answer for each of the comments and suggestions you raised.

COMMENTS
Results & Discussion

Gene expression: The gene expression analyses provide a step toward connecting the GWAS-identified Z-linked region to a molecular mechanism, but the results remain preliminary, which the authors acknowledge themselves. Of the 12 candidate genes tested, only two showed significant genotype-dependent expression (PDE8B in males, WDR41 in females), and only one of these (PDE8B) was significantly associated with spottiness in males, but not coloration.
However, the genotype distribution for MC1R was highly unbalanced (38 homozygous white, 6 heterozygous, 1 homozygous red), which precluded the analysis of MC1R expression effects entirely. This limits interpretation given that MC1R is the locus with the strongest evidence for selection. It would strengthen the manuscript if the authors elevated their discussion of these limitations, particularly regarding the inability to account for MC1R genotype variation in the expression models and the single significant expression-phenotype link. This may help in framing the gene expression section more proportionally to the strength of the evidence it provides. As it stands, the narrative gives this component somewhat more interpretive weight than the data currently support.

[R] We thank the reviewer for this thoughtful and constructive comment. We agree that the gene expression analyses provide an initial step toward linking the GWAS- identified Z- linked region to a potential molecular mechanism, but that the evidence remains preliminary and should be interpreted cautiously.

As noted by the reviewer, only two of the twelve candidate genes tested showed significant genotype- dependent expression (*PDE8B* in males and *WDR41* in females), and only one of these (PDE8B) was significantly associated with a phenotypic trait (spottiness in males). In addition, the highly unbalanced genotype distribution at the *MC1R* locus in the expression dataset prevented a meaningful assessment of MC1R expression effects, despite *MC1R* showing the strongest evidence for selection in the population‑level analyses. This is an important limitation of the gene expression component.

To address these points, we have revised the manuscript to acknowledge more explicitly these limitations and to better align the interpretation of the gene expression results with the strength of the underlying evidence. Specifically, we have tempered the language in the Results and Discussion to emphasize that the expression analyses identify candidate genes and suggest possible regulatory links, rather than providing definitive mechanistic explanations. We now explicitly highlight the inability to assess *MC1R* expression effects and clarify that the single significant expression-phenotype association should be viewed as hypothesis‑generating.

These revisions ensure that the gene expression component is presented as supportive and exploratory and more accurately reflect its contribution relative to the genomic and evolutionary analyses.

We have now integrated these modifications line 248:

"To explore potential molecular correlates of the GWAS- identified Z- linked region, we conducted exploratory targeted gene expression analyses."

And line 275:
"Only a small subset of tested genes showed genotype-dependent expression, and only one gene (*PDE8B*) was associated with a phenotypic trait, specifically spottiness in males. Moreover, the strongly unbalanced genotype distribution at the *MC1R* locus in the expression dataset precluded an assessment of *MC1R* expression effects, despite *MC1R* showing the strongest signal of selection at the population level."

Simulation parameters: The authors estimate Ne at ~226,000, which they attribute to high historical effective population size and contemporary connectivity across European barn owl populations. However, they use the census population size of N = 400 for the forward drift simulations, which is described as a conservative estimate consistent with the lowest census sizes during the study period. We appreciate the conservative framing, but given the large difference between these two values, it would be helpful if the authors could more explicitly discuss why census N is the more appropriate parameter for the drift simulations and how sensitive the results are to this choice. Using a smaller population size produces wider neutral envelopes, which would make drift easier to reject. As a reader, it would be beneficial to understand whether the selection inference at MC1R holds under alternative population size assumptions closer to Ne.

[R] We agree that using a smaller population size produces a wider neutral envelope, hence making drift harder (not easier) to reject. Our choice to parameterize the forward simulations using census population size (N = 400) was motivated by the specific aim of these simulations, which is to evaluate whether the observed short‑term allele frequency changes over approximately three decades could plausibly arise from stochastic drift in the locally breeding population. Over such contemporary timescales, drift is more appropriately governed by the number of breeding individuals contributing alleles each year, rather than by the long‑term effective population size inferred from historical genome‑wide diversity and continental connectivity.

Importantly, we do not rely on this conservative choice alone. To directly address the reviewer's concern, we re- ran the forward simulations across a range of population sizes, including N = 400 (the census size), N = 1,000 (consistent with values inferred from ApproxWF) and N = 10,000 (approaching the order of magnitude of the Ne estimated from nucleotide diversity while remaining computationally tractable). These additional simulations were performed in combination with varying initial allele frequencies, reflecting uncertainty in early sampling.

As expected, increasing population size narrows the envelopes for both neutral and selection- inclusive scenarios. However, across all tested population sizes and initial allele frequencies, the qualitative results remain unchanged: the observed allele frequency trajectory at the MC1R locus is unlikely under drift alone and is consistently better explained by models incorporating selection. These results confirm that our inference of selection at MC1R is robust to alternative population size assumptions, including values substantially closer to the estimated long- term Ne.

We have now clarified the rationale for using census population size in the main text and added the results of these sensitivity analyses to the Supplementary Information (Fig. S27).

See line 401:
"Inference of selection at MC1R was robust to population size assumptions, as simulations performed with larger population sizes (N = 1,000 and N = 10,000) yielded qualitatively similar conclusions, with the observed trajectory remaining unlikely under drift alone (Fig. S27)."

And line 995:
"This choice reflects the short- term, contemporary timescale of the analysis, for which stochastic allele frequency change is primarily driven by the number of breeding individuals contributing alleles each year, rather than by long- term effective population size inferred from historical genome- wide diversity and large- scale connectivity. To test if the inference of selection at MC1R was robust to population size assumptions, we also performed simulations with larger population sizes (N = 1,000 and N = 10,000)."

Analytical roadmap: We found it somewhat difficult to follow the reasoning for which loci were chosen for the simulation analyses. For instance, why was MC1R prioritized for the selection-versus-drift analysis over the Z-linked locus, given that the Z-linked region is associated with both traits and shows the larger effect on spottiness? We recognize that both loci are ultimately analyzed, but the narrative structure could benefit from a clearer explanation of the decision-making process guiding which loci were modeled and why.
Along these lines, the manuscript presents a series of increasingly focused analyses, but the subset of loci carried forward into each stage differs without always making the rationale explicit. For instance, CORIN is included in the coloration genetic architecture models but not in the allele frequency or simulation analyses, and the Z-linked region is represented by different top SNPs across analyses (all in high LD, but the positions differ). While each individual analysis appears well-executed, we think the manuscript would benefit from a brief roadmap clarifying which loci

are carried into each analytical stage and why. As a reader, we felt this would help track the decision-making logic without having to reconstruct it across sections.

[R] We agree that the logic linking successive analytical steps was not always made sufficiently explicit in the previous version of the manuscript.

Our analytical workflow proceeds from locus discovery to increasingly focused evolutionary analyses, but we recognize that the criteria used to carry specific loci forward into each stage were not clearly articulated. To address this, we have revised the manuscript to clarify which loci are considered at each analytical step and the rationale for their inclusion or exclusion.

Specifically, the CORIN locus was included in the genetic architecture models for plumage coloration because it was identified as a significant locus in the region‑specific GWAS and contributes to explaining phenotypic variance, but it was not originally carried forward into the allele‑frequency and simulation analyses because its effect size is smaller and its signal was limited to a subset of coloration traits. In response to the reviewer's comment, we have now extended the allele‑frequency and simulation analyses to include CORIN, and these additional results are presented in the main text (figure 5).
Similarly, the Z- linked region is represented by different SNPs across analyses. All of these SNPs are in very high linkage disequilibrium and we assumed they would capture the same underlying genetic signal. We have now confirmed that with the selection analysis conducted on both SNPs on the Z locus (the top SNP associated with the color, Fig. 5, and the top SNP associated with the spottiness Fig. S28).

For in text modifications see line 360:
"To investigate the evolutionary forces underlying allele frequency changes at the loci associated with color and spottiness, we leveraged the temporal resolution of our 30-year dataset to track allele dynamics over time (see Fig. 5 for *MC1R*, *CORIN* and the Z locus associated with spottiness; and Fig. S25 for the Z locus associated with the color)."

The SNP used for genotyping and gene expression analyses differs from the top GWAS SNP for technical reasons related to probe design; we now make this distinction explicit in the Methods and Results sections.

See line 767 (method).
"To validate associations found on the Z chromosome, we performed an allelic discrimination assay targeting a SNP located on Super-Scaffold 42. The two top SNPs identified in the GWAS for plumage coloration and spottiness could not be directly genotyped due to probe design constraints. We therefore selected a nearby SNP (at position 29,811,381) for which probe design was feasible. This SNP showed …"
And line 260 (results / discussion)
"We first designed an assay to genotype the individuals at the Z locus (see method section for details) and confirmed that genotypes at the genotyping SNP were significantly associated with both coloration and spottiness in these individuals (Fig. 3b, 3c)."

Together, these revisions ensure that all loci identified as contributing to phenotypic variation are consistently tracked throughout the manuscript, and that the decision- making logic underlying each analytical step is transparent to the reader.

Phenotypic scoring methodology: The authors employ an eight-point visual color chip scale for scoring plumage coloration. While this approach has been used in prior studies, there is now a substantial body of work employing spectrophotometry and digital imaging for quantifying melanin-based plumage variation in birds. Have the authors compared or validated this visual scoring method against more objective quantitative approaches? If validation studies exist for this scoring system, citing them would help readers assess the precision and repeatability of the phenotypic measurements underpinning the study.

[R] We agree that advances in spectrophotometry and digital imaging now provide powerful quantitative tools for characterizing melanin□based coloration, and that clarity regarding the validation of visual scoring methods is essential for assessing the robustness of phenotypic analyses.

The eight- point visual color chip scale used here is a long- established method in barn owl research and has been extensively validated in previous studies. Repeatability analyses based on repeated scoring of the same individuals within a season have shown very high consistency, with an average repeatability of 0.90 across melanin□based plumage traits (Roulin, 1999, 2004). This high repeatability indicates that the method provides precise and reliable phenotypic measurements.

Importantly, visual color scores have also been directly validated against spectrophotometric measurements of pheomelanin- based coloration. In a large subset of individuals for which feather samples were available, brown chroma (R600-700/R300-700), measured using reflectance spectrometry, was highly correlated with visual color scores (Pearson's $r = -0.78$, $P < 0.0001$, $N = 1107$) (Dreiss & Roulin, 2010). This strong correlation demonstrates

that the visual scoring system captures the same underlying variation in melanin- based coloration as more objective quantitative approaches.

To address the reviewer's suggestion and improve transparency, we have now expanded the Methods section to explicitly describe the rationale, repeatability, and validation of the visual scoring system. In addition, we include supplementary material providing representative images of each color category (See figure S1). Together, these additions clarify the precision and biological relevance of the phenotypic measurements underpinning our analyses.

For in text modifications see line 487:
"The degree of reddish pheomelanic coloration was scored using an eight-color chip scale ranging from −8 (white) to −1 (dark reddish). See Fig. S1 for example photographs of barn owls being score using the chip scale. This method has been shown to be highly repeatable, with repeatability estimates averaging 0.90 when individuals are scored twice within the same season 62. Visual color scores have also been validated against spectrophotometric measurements of pheomelanin-based coloration. In a large subset of individuals, visual scores were strongly correlated with brown chroma (R600-700/R300-700) measured using reflectance spectrometry (Pearson's $r = -0.78$, $P < 0.0001$, $N = 1107$) 45, demonstrating that this scoring system reliably captures biologically meaningful variation in plumage coloration."

Environmental contributions to the phenotypic trend: The phenotypic trend models in supplement use year of birth as a predictor without environmental covariates, and year is inherently confounded with any directional environmental change over the study period. The allele frequency analyses provide convincing independent evidence that the phenotypic trends have a genetic basis, and melanin-based plumage traits are generally considered to have low environmental plasticity. Nonetheless, a brief acknowledgment that the phenotypic models alone cannot fully disentangle genetic from environmental contributions to the temporal trend would add useful nuance, particularly for readers less familiar with the heritability of melanin-based traits.

[R] We agree that modeling phenotypic change as a function of year of birth alone does not allow a full disentanglement of genetic and environmental contributions, as year is inherently confounded with any directional environmental change over the study period. Our intent in presenting these phenotypic models was to document the existence and magnitude of long‑term temporal trends and inferring their underlying causes remains to be addressed in the future.
To clarify this point and add nuance, we have revised this section of the manuscript to explicitly acknowledge this limitation. See line 175:
"Because years are confounded with any directional environmental change over time, these phenotypic models do not, on their own, allow a full disentanglement of genetic and environmental contributions to the observed trend."

Reviewer #2 (Remarks to the Author):

I co-reviewed this manuscript with one of the reviewers who provided the listed reports. This is part of the Nature Communications initiative to facilitate training in peer review and to provide appropriate recognition for Early Career Researchers who co-review manuscripts.

[R] We thank the co- reviewer for their positive feedbacks. The points raised are addressed in our responses to Reviewer 1.

Reviewer #3 (Remarks to the Author):

In the article “Genomic bases of short-term evolution in the wild revealed by long-term monitoring and population-scale sequencing,” the authors leverage a 30-year dataset of barn owls to connect genotype with phenotype and study their changes over time. This is a truly enormous dataset that provides a unique opportunity to study plumage color in birds! I have a few major concerns that I detail below that will help clarify a number of points in the manuscript, though I suspect these will not change the primary results of the paper. Overall, this was an exciting read and I believe there will be a lot of interest in the results from the ornithology, evolutionary biology, and color evolution readership!

[R] We thank you for your feedback and constructive comments on our manuscripts. Please find below a detailed answer for each of the questions you raised.

MAJOR COMMENTS:

(1) One of my primary concerns is around the framing of the paper and how this matches with the actual results that are included. The abstract and introduction set up the focus of the paper to include fitness and climate change (e.g., L42-44), but these felt very underdeveloped in the included results. Much of the argument the authors have built rests on previous connections between fitness and plumage color/spottiness (L88-90), but there aren’t enough details included here to understand how/why that relationship might exist. What is the directionality of the relationship? (Just based on the results included here, I assume darker/spottier is better…?) What is the suspected mechanism underlying plumage’s role? And perhaps most importantly, why do you expect this is changing over time? Based on the abstract I was expecting there would be an analysis connecting observed phenotypic changes to some kind of environmental variable and a direct assessment of individual survival/reproduction based on phenotype to show how these have changed over time. It seems like information on offspring survival exists, is it possible to integrate that directly into this manuscript? Further exploring this would help support the latter simulation results on the role of selection versus drift.

[R] We agree that the initial framing of the manuscript did not sufficiently align with the scope of the analyses presented, and we appreciate the opportunity to clarify this point.

The main objective of this study is to document and evaluate long- term temporal changes in plumage coloration and spottiness, and to assess whether the observed patterns are more consistent with stochastic processes (e.g. drift) or directional processes (e.g. selection), rather than to directly quantify fitness consequences or identify specific environmental drivers. We recognize that the original abstract and introduction, by emphasizing fitness and climate change, may have suggested a stronger inferential link than the analyses can directly support.

Our expectation that plumage traits might evolve over time was motivated by a substantial body of previous work linking coloration and spottiness to multiple fitness components. Because different color and spottiness patterns have been associated with different advantages (e.g. survival, reproduction, or condition), we did not have a strong a priori prediction regarding the direction of phenotypic change. Rather, we aimed to test whether change occurred at all, and whether its magnitude and structure were compatible with selection versus drift.

We fully agree that directly linking phenotypic change to environmental variables and individual fitness would be an important next step. While some information on offspring survival exists in our system, these data do not allow for reliable estimates of individual fitness. Nest checks are conducted approximately 40 days after hatching, which means that some of the oldest offspring may already have fledged or died, and subsequent recaptures are incomplete due to the open nature of the population. Additionally, we expect strong interannual variation in demographic conditions, further complicating the estimation of meaningful fitness-phenotype relationships. For these reasons, integrating these proxies into the present manuscript would risk introducing substantial bias and potentially misleading inference.

Importantly, our revision now makes these limitations explicit. We have modified the abstract and introduction to clarify that the study focuses on temporal phenotypic change and the relative roles of drift and selection, rather than on direct estimates of fitness or explicit environmental drivers. The latter remain open and important questions, which we highlight as priorities for future research.

We hope that these changes better align the framing of the manuscript with the data and analyses presented, and we thank the reviewer for helping us improve the clarity and transparency of the study.

See the revised abstract, as well as modification along the text lines 57:

"requires understanding how genetic variation translates into phenotypic variation and how evolutionary processes such as drift and selection shape these traits over time"

line 136:
"To characterize the genetic architecture underlying this trend, and to assess whether the observed temporal allele frequency changes are more consistent with directional selection or stochastic processes"

And line 953:
"Allele frequency changes and inference of evolutionary processes"

(2) I had a few concerns about the dataset that I feel need to be addressed. The largest concern is about the difference in sample size of the first year of the data (1992). As shown in Figure S4 and S5, the sample size of each of the sexes is quite small in this year, which seems to heavily influence the distribution of observed phenotypes (such that the means are pretty different from the subsequent years). I think this isn't necessarily a huge problem, but I do wonder if this is influencing things like the simulation analyses included in Figure 5. The MC1R simulations have an observed allele frequency that starts nearly at 0! How influential is this initial year in the identified changes over time? The allele frequencies shown in Figure S23 seem pretty different for those early years (for both MC1R and Z), so I think it will be really important to confirm that the lower sample size is not creating an outsized effect on the overall observed patterns across different analyses.

[R] We agree that the small number of individuals sampled in the earliest years of the study (notably 1992) warrants careful consideration, particularly with respect to allele frequency estimation and downstream temporal analyses.

With respect to the phenotypic analyses, the temporal trends in coloration and spottiness are inferred using linear mixed- effects models that leverage information across the full time series and account for year- to- year variation. Because these models are driven by long- term trends rather than single years, they are not unduly influenced by the earliest years with limited sample sizes. To confirm this explicitly, we have re- run the phenotypic models restricting the dataset to the last 25 years of monitoring, during which sample sizes are consistently large and representative of the population. These analyses yield qualitatively similar estimates of temporal change for both traits. We now report these results in the main text (lines 171-173).

We also fully agree that low sample sizes in the early years can introduce uncertainty in allele frequency estimates. We therefore took several steps to ensure that this uncertainty does not bias our inferences in our first version of the manuscript. First, rather than relying on a single year to define initial allele frequencies, we estimated starting frequencies by pooling data across the earliest years of the study, thereby reducing sensitivity to stochastic variation associated with very small samples. Second, all forward simulations explicitly incorporate year- specific sampling by subsampling simulated individuals to match the empirical number of genotyped adults alive in each year. As a result, confidence intervals around simulated trajectories are wider in early years and progressively narrow as empirical sample sizes increase, mirroring the uncertainty present in the real data.

Finally, to directly assess the influence of the assumed initial allele frequency on our inference of selection at the MC1R locus, we re- ran the simulations across a range of plausible initial frequencies (0.025-0.15), spanning the uncertainty observed in the early empirical estimates. Across this range, the qualitative conclusions remain unchanged: the observed allele frequency trajectory is consistently unlikely under drift alone and is best explained by models incorporating selection. We now include these sensitivity analyses in the Results and Supplementary Information.

See lines 401-404:
"Inference of selection at MC1R was robust to population size assumptions, as simulations performed with larger population sizes (N = 1,000 and N = 10,000) yielded qualitatively similar conclusions, with the observed trajectory remaining unlikely under drift alone (Fig. S27)."

Taken together, these additional analyses confirm that although uncertainty is highest in the earliest years of the study, as expected given sample size limitations, this uncertainty is accounted for and does not drive the main conclusions regarding phenotypic trends or the inference of evolutionary processes.

(3) Finally, I believe more details on the classification of color are needed here. There is very little detail included on the determination of the pheomelanin coloration. What does it actually mean to be -8 versus -1 on your color chips? What color chips are you actually using?

[R] We thank the reviewer for requesting additional clarification regarding the classification of pheomelanin- based coloration. Plumage coloration was assessed using a standardized eight- point visual color chip scale capturing continuous variation in ventral pheomelanin coloration, ranging from −8 (very light, nearly white plumage with minimal reddish coloration) to −1 (dark, strongly rufous plumage). Intermediate scores represent gradual increases in reddish- brown coloration rather than discrete categories.

The color chips are standardized reference chips routinely used in our long- term barn owl studies and allow consistent visual matching of ventral plumage by trained observers. This system has been widely applied across decades of monitoring, facilitating direct comparison among studies.

To improve transparency, and in line with the suggestions of Reviewers 1 and 2, we have expanded the Methods section to explicitly define the color scale and scoring procedure, as well as to expand on the validation of this field- based assessment using spectrophotometry (see line 487-495). In addition, we provide a new supplementary figure (Fig. S1) showing a field acquisition of the two phenotypes (color and spottiness), allowing readers to better understand how the phenotypes are measured.

"The degree of reddish pheomelanic coloration was scored using an eight-color chip scale ranging from −8 (white) to −1 (dark reddish). See Fig. S1 for example photographs of barn owls being score using the chip scale. This method has been shown to be highly repeatable, with repeatability estimates averaging 0.90 when individuals are scored twice within the same season 62. Visual color scores have also been validated against spectrophotometric measurements of pheomelanin-based coloration. In a large subset of individuals, visual scores were strongly correlated with brown chroma (R600-700/R300-700) measured using reflectance spectrometry (Pearson's $r = -0.78$, $P < 0.0001$, $N = 1107$) 45, demonstrating that this scoring system reliably captures biologically meaningful variation in plumage coloration."

Additionally, there are a number of questions about the developmental patterns of the plumage color and spotting that should be addressed. How does plumage color in nestlings compare to adults? Is it actually reasonable to lump both nestlings and adults into the same dataset for the analyses you include in this manuscript? Is including related family groups within the dataset biasing your results? Does the plumage color change as adults age? Especially as "stage [fledgling]" pops up as a significant predictor in many of the models included in the supplement and Figure S7 suggests there are significant differences between adults and juveniles, it seems important to address these issues directly.

[R] We thank the reviewer for raising these important points regarding developmental patterns in plumage coloration and spottiness, and for the opportunity to clarify how age and relatedness were accounted for in our analyses.

In barn owls, melanin- based plumage coloration and spottiness are known to change slightly during early life, with most age- related change occurring within the first year, after which coloration and spotiness remains largely stable in adulthood. This pattern has been previously documented (Dreiss & Roulin, 2010). To account for this known ontogenetic variation, we explicitly included developmental stage at phenotyping (fledgling versus adult) as a fixed effect in all phenotypic and genetic models.

This modeling choice allows us to combine data from nestlings and adults while statistically controlling for age- related differences, rather than assuming that coloration is identical across life stages. As you pointed out, developmental stage explains a significant portion of variation in coloration in Fig. S7, which is consistent with previous work, but does not alter the inferred temporal trends.
Finally, we note that although plumage coloration changes slightly early in life, there is no evidence for progressive age- related change across adulthood. We have now expanded the Methods section to clarify these points and added the relevant reference to explicitly justify the inclusion of developmental stage as a covariate.

Regarding potential bias from family structure, relatedness among individuals is explicitly accounted for in our analyses. In the quantitative genetic models, relatedness is incorporated through the genomic relationship matrix, while GWAS and downstream analyses use mixed- model frameworks that control for kinship. These approaches minimize bias arising from the inclusion of related individuals.

See changes line 519:
"The best supported model for both traits included the following fixed effects: year of birth (continuous) to test for long-term evolutionary trends; sex (categorical) to account for sexual dimorphism in plumage traits 62,65, Fig. S7); and phenotypic stage (categorial: fledging or adult) to control for age-related changes in melanin expression and for age- related variation in coloration known to change slightly during early life but remains largely stable after the first year 45,46, Fig. S8)."

Line 162:
"This trend was consistent across both males and females showing increased melanism over time (Fig. S5-S6, Tables S5-S8 for sex-specific models), despite the species' weak sexual dimorphism (Fig. S7) and accounting for the slight coloration difference between fledglings and adults (Fig. S8)."

As well as line 929:
“We also included the developmental stage at phenotyping (fledglings versus adults) as a fixed factor, to control for age-related variation in coloration”

MINOR COMMENTS:

It could be helpful to include an in-text table that lists the identified genes and which trait they are associated with.

[R] We have added Table S21 in the Supplementary Material, which summarizes all genes harboring at least one SNP significantly associated with the traits across the GWAS analyses, and indicates for each gene the corresponding trait(s).

L63-70. This introduction here feels a bit out of date… many studies have now studied phenotypes in wild populations, particularly for bird coloration.

[R] We agree that the original wording sounded somewhat outdated. We note that the purpose of this paragraph is not only to highlight challenges in identifying the genetic basis of traits, but more broadly to emphasize the difficulty of linking phenotypic change over time to its underlying evolutionary and genomic mechanisms in natural populations.
We have therefore revised this paragraph to tone down the wording to better reflect the current state of the field and align it with this broader aim. In particular, we replaced “remains a major challenge” with a more balanced formulation (“remains challenging”, line 64), which acknowledges both recent advances and the remaining difficulties. This revised wording better captures that perspective.

L72-83. Additionally, I felt that many of these citations were not well matched with the introduction here. They felt both overly broad and then also oddly specific in different places. There is a TON of literature on the genomics of bird coloration that was overlooked and I think would greatly strengthen the intro.

[R] We have revised the paragraph to better incorporate key advances in this field, including recent reviews on avian coloration genetics and genomic architecture (e.g., Orteu & Jiggins 2020; Price-Waldman & Stoddard 2021). We also restructured the paragraph to more clearly emphasize birds as a model system and to highlight the current state of the field, while maintaining our focus on the relative lack of studies on continuously varying coloration traits. see section lines 71 to 86 for the new version of the paragraph.

L96. I was a bit confused about why explaining only 80% of the variation in this traits was used as a motivator for the study here. This feels like a HUGE amount of variation when compared to most studies of wild organisms, so this didn’t feel particularly exciting as a motivator.

[R] We agree that explaining ~80% of the variation represents a substantial achievement for a trait in a wild population, and that our original wording did not adequately reflect this.
We have therefore revised the paragraph to explicitly acknowledge the largely oligogenic architecture of coloration, while clarifying that the remaining unexplained variation likely reflects additional loci or interactions. More importantly, we rephrased the motivation to emphasize the key outstanding questions, namely the genetic basis of spottiness, the extent of shared genetic architecture between traits, and the role of sex-specific effects, rather than focusing solely on the proportion of variance explained.
see line 126 for the new version of the paragraph.
“[…] Together, these loci explain a large proportion of the variation in coloration, highlighting a relatively simple genetic architecture for this trait. However, the remaining variation suggests additional genetic factors or interactions that remain to be characterized. In parallel, the genetic basis of spottiness has yet to be investigated. […] ”

L127-128. Given this population is open to immigrants and the trend holds with them as well, what is the expected selection pressure? It seems like it would need to be something that is working on a continental or global scale?

[R] Because the population is open and the temporal trend is observed in both residents and immigrants, our results suggest that the underlying selective pressures are unlikely to be restricted to the focal population. Instead, they are more consistent with selection acting over a broader spatial scale, potentially at the regional or continental level, in line with the high connectivity and low genetic differentiation reported across European barn owl populations.
However, given that our study focuses on a single focal population, we cannot directly quantify the geographic scale of selection, and distinguishing between regional versus continental (or larger-scale) selective regimes would require comparable temporal data from other populations (but see (Romano et al., 2024) for evidences of changes in color at more global scales).

To emphasize this idea, we have now modified the main text line 167:
“This pattern further suggests that the observed evolutionary trend is not restricted to the focal population, but likely occurs over a broader spatial scale.”

L194. Typo in “our work results refine…”

[R] Fixed

L195-197. What trait was this gene related to in rosy finches? Does it seem to be doing the same thing as in barn owls?

[R] the trait in rosy finches was the coloration of the “crown patch”, when contrasting the normal with a darker morph of the patch. We have now add this information in the main text.

L231. Was this also in birds?

[R] Unfortunately we couldn’t find any study of the effect of *PDE5* (or mention) in birds and all references are human related. This is now stated in the main text.

L249-264. The discussion of CORIN was quite disjunct here. It was only mentioned once briefly before this and then is pretty underdeveloped here. Particularly as this gene has been found related to coloration in other birds, it seems like it might be helpful to go into a bit more detail. (e.g., Wood et al. 2023 Current Biology, Bourgeois et al. 2016 J Heredity)

[R] We agree that the discussion of CORIN was underdeveloped in the previous version of the manuscript. While CORIN was identified as a significant locus contributing to coloration, we did not sufficiently place this result in the context of existing work on pigmentation in birds.
We have now expanded this section to better contextualize CORIN by explicitly discussing its known role in coloration in other avian systems and by citing relevant studies. These additions clarify how our findings extend previous work and highlight CORIN as a recurrent contributor to avian pigmentation variation, while maintaining appropriate emphasis given its smaller effect size relative to MC1R and the Z- linked locus.

See line 314 for in-text modification:
“The significant effect of CORIN, however, represents a novel result in this population, but is consistent with an emerging role of CORIN in avian coloration more broadly. CORIN has been implicated in pigmentation variation in several bird species, including associations with melanin- based coloration and patterning, and may act as a modifier locus influencing pigmentation pathways).”

L249. Missing parenthesis

[R] Fixed

L263-264. This statement feels a bit overblown…

[R] We have revised this sentence to replace “proven” with more appropriate language reflecting empirical support rather than definitive proof.
line 325:
“This epistatic interaction has been previously hypothesized [46] and is here supported by genomic data.”

L323-324. But you’re now talking about range-wide selection, no?

[R] We thank the reviewer for pointing out this potential ambiguity in scale. Our intent here was to refer to evolutionary dynamics at the level of the locally breeding Swiss population, rather than to range- wide selection. Although long- term effective population size reflects historical connectivity across Europe, we think that short- term allele- frequency change over the study period is governed by the census size of the local breeding population. We have revised the text to clarify this distinction and to explicitly state the spatial scale at which drift and selection are evaluated.
See line 382:
“To assess whether the observed allele frequency changes could be attributed to selection rather than genetic drift, we considered the relationship between allele effects on fitness and population size. For natural selection to act efficiently, the variant must influence fitness, and the effective population size (Ne) must be large enough for selection to overcome stochastic fluctuations due to drift. In small local populations, drift can dominate, causing allele frequencies to change regardless of selective advantage. Although long- term effective population size reflects historical connectivity at a broader geographic scale, census estimates for the locally breeding Swiss barn owl population remained relatively stable over the study period, ranging from 200 to 1,000 breeding pairs.”

L325. It could be helpful to indicate where the mutation rate estimate is coming from. (I know you include it in the methods, but I would just add it here too.)

[R] Since our first version of the manuscript, we now have an estimate of the mutation rate for the European barn owl (estimated in the same population). We have thus revised our estimates of Ne accordingly and explain the origin of the mutation rate in the main text as well as in the material and method section.

See line 391:
“We adopted the recently estimated mutation of the barn owl (Mu = 5.6*10^-9) [57].”

And 977:
“We adopted the recently estimated mutation rate for the barn owl (*tyto alba*) (Mu = 5.6 *10-9) [57].”

L357. Missing a word here: “further supporting the [role] of selection”

[R] Fixed

L360. Missing a word here: “between the selection and drift models [was] modest”

[R] Fixed

L409. Equation shows a square here—likely an error post-submitting

[R] We thank the reviewer for pointing this out. We carefully checked the resubmitted manuscript and ensured that the equation is correctly displayed and corrected any formatting issues.

L413. Missing words here: “The life stage at which [an] individual was phenotype[d]”

[R] Fixed

L582. Missing a word here: “lead to artificially [elevated?] accuracy due to”

[R] Fixed

L593. Hanging sentence here. What was excluded?

[R] The sentence got mixed-up during the reviewing process, this is now fixed.
see line 685:
“To ensure fair evaluation, we excluded 11 individuals from the validation set that had intermediate sequencing depth in the high-coverage dataset (mean ~ 11x), as their coverage could bias the accuracy assessment (see[34] for details).”

L712. Typo: “Quiagen” should be “Qiagen”

[R] Fixed

L919. Typo: “Montel Carlo” should be “Monte Carlo”

[R] Fixed

L933. What does “a substantial portion of MCMC iterations” mean here?

[R] In our analyses, loci were evaluated using three independent criteria, and we agree that the third criterion required clarification. In the approxWF reversible- jump framework, transitions between neutral and selection models are proposed at a fixed baseline rate (default 10%). We have now revised the Methods section to explicitly state that the proportion of iterations spent in the selection model is interpreted relative to this baseline, with values close to 10% indicating little support for selection and higher values indicating increasing relative support. This clarification better reflects how this criterion was used in combination with likelihood comparisons and credible intervals.

See line 1037:
“ We then fitted a selection-only model and assessed whether the 95% credible interval of the estimated selection coefficient excluded zero. A locus was considered under directional selection if it met the following three criteria: (i) the selection model had a higher log-likelihood than the drift-only model; (ii) the 95% credible interval did not include zero; and (iii) the MCMC chain spent a greater proportion of iterations in the selection model relative to the baseline proposal rate. In the approxWF reversible-jump framework, transitions between neutral and selection models are proposed at a fixed rate (default 10%), and deviations from this baseline are interpreted as preferential sampling of the model including selection. ”

FIGURE COMMENTS:

Figure 1B: This scatterplot was very difficult to interpret! Especially in comparison to the same trends shown in Figure S2. It would be helpful to change the opacity of the points and/or leverage point size to clearly display the overlap of points, add in a trendline to make the relationship clearer, and do away with the different point shapes (which I am inferring relate to sex?). The size of the figure makes it difficult to even differentiate shapes here, and I suggest leaving the separate plots to Figure S2.

[R] We agree that the original version of Figure 1B was difficult to interpret. The different point shapes indeed corresponded to sex, but this was not clearly explained and did not add substantial information beyond what is already presented in Figure S2.
We have therefore simplified the plot by using a single point shape and increasing transparency to better represent point density and overlap. In addition, we added a fitted linear regression line to more clearly highlight the relationship between coloration and spottiness.

Figure 2: As there is a lot of whitespace here, it would be helpful to include some labelling within this figure. Perhaps you could add gene names to A and B, and indicate which GWAS is for color vs spots? It would also be helpful to add some more labelling details to C, D, and E. It could be worth highlighting which genes are the ones of interest in the middle gray bars, so it's clear where they are located in association to the GWAS peak. Finally, the LD plots are slightly offset from the axis of the GWAS peak, and it would be helpful to make sure those match up one-to-one.

[R] We have revised the figure accordingly to reduce whitespace and make the different analyses easier to interpret. First, we now explicitly label each GWAS panel to indicate whether it corresponds to coloration or spottiness, and we added consistent pictograms that are reused in the zoomed panels (C-E) to facilitate visual correspondence between panels.
We also highlighted the MC1R gene directly in panel C, as this locus is a central focus of the study. For the Z-linked region, we did not highlight individual genes in Figure 2, as the same gene set are presented and discussed in Figure 3.
In addition, we corrected the alignment between the GWAS peaks and the LD plots. The slight offset in the original version was due to the fact that not all SNPs in upper pannel were included in the LD calculation. We now restrict the LD plots to the same set of SNPs shown in the zoomed GWAS panels, ensuring a one‑to‑one alignment between association signals and LD structure.

Figure 3: The font in this figure is particularly small, and it would similarly benefit from additional labelling within the figure. Especially as the different panels move from phenotype to genotype and back again as you go from top to bottom.

[R] We have increased the font size throughout the figure to improve legibility.
Regarding additional labelling, we note that the axes, legends, and panel annotations already explicitly indicate the nature of the data shown at each step. To further clarify the structure of the figure and guide the reader through the transition from genotype to expression and phenotype across panels, we have revised the figure title to more clearly emphasize (i) the zoomed‑in focus on the Z‑linked region initially identified in Figure 2 and (ii) the explicit links between genotype, gene expression, and phenotype. We believe these changes improve clarity while keeping the figure visually readable and avoiding excessive labelling.

Figure 5: It would be helpful to add "MC1R" and "Z" as labels to the top and bottom panels here. Additionally, the message shown in A/D will be clearer if the s=0 line is marked on the heatmap, as that will very visually show that the MC1R analysis results in s>0. Finally, are the dark brown lines shown in B/C/E/F the observed allele frequency (as compared to the simulation shown in lightly lines)? If so, it would be helpful to include that info in the legend, and potentially change the color / thickness of the line so that it's really distinguishable from the lines indicating the outside edges of the simulations.

[R] We have revised Figure 5 accordingly to improve clarity and interpretability.
We now explicitly label each panel with the corresponding locus. We have also added the horizontal line corresponding to s=0in panels A, D, and Gm which makes it easier to visually assess deviations from neutrality and highlights the signals of positive selection.
In addition, observed trajectories are now more clearly distinguished from the simulated distributions by adjusting their color and thickness, and this information has been explicitly described in the figure legend.
Finally, following this revision, we extended the analysis by including the CORIN locus, which is now presented alongside MC1R and the Z-linked locus in Figure 5.
